# 8-Deoxy-Rifamycin Derivatives from *Amycolatopsis mediterranei* S699 *ΔrifT* Strain

**DOI:** 10.3390/biom10091265

**Published:** 2020-09-02

**Authors:** Feng Ye, Yanrong Shi, Shengliang Zhao, Zhiying Li, Haoxin Wang, Chunhua Lu, Yuemao Shen

**Affiliations:** 1 Key Laboratory of Chemical Biology (Ministry of Education), School of Pharmaceutical Sciences, Cheeloo College of Medicine, Shandong University, No. 44 West Wenhua Road, Jinan 250012, China; yefeng1997@mail.sdu.edu.cn (F.Y.); yrshi910212@sdu.edu.cn (Y.S.); 201720261108@mail.sdu.edu.cn (S.Z.); 201936104@mail.sdu.edu.cn (Z.L.); ahua0966@sdu.edu.cn (C.L.); 2 State Key Laboratory of Microbial Technology, Shandong University, Qingdao 266237, China; wanghaoxin@sdu.edu.cn

**Keywords:** rifamycin, *Amycolatopsis mediterranei* S699, proansamycin X, dehydrogenation, *N*-glycoside rifamycin

## Abstract

Proansamycin X, a hypothetical earliest macrocyclic precursor in the biosynthesis of rifamycin, had never been isolated and identified. According to bioinformatics analysis, it was proposed that RifT (a putative NADH-dependent dehydrogenase) may be a candidate target responsible for the dehydrogenation of proansamycin X. In this study, the mutant strain *Amycolatopsis mediterranei* S699 *ΔrifT* was constructed by deleting the *rifT* gene. From this strain, eleven 8-deoxy-rifamycin derivatives (**1**–**11**) and seven known analogues (**12**–**18**) were isolated. Their structures were elucidated by extensive analysis of 1D and 2D NMR spectroscopic data and high-resolution ESI mass spectra. Compound **1** is a novel amide *N*-glycoside of *seco*-rifamycin. Compounds **2** and **3** feature conserved 11,12-*seco*-rifamycin W skeleton. The diverse post-modifications in the polyketide chain led to the production of **4**–**11**. Compounds **2**, **3**, **5**, **6**, **13** and **15** exhibited antibacterial activity against *Staphylococcus aureus* (MIC (minimal inhibitory concentration) values of 10, 20, 20, 20, 40 and 20 μg/mL, respectively). Compounds **14**, **15**, **16**, **17** and **18** showed potent antiproliferative activity against KG1 cells with IC_50_ (half maximal inhibitory concentration) values of 14.91, 44.78, 2.16, 18.67 and 8.07 μM, respectively.

## 1. Introduction

Ansamycins are a type of macrocyclic antibiotics formed by an aromatic moiety bridged at nonadjacent positions by an aliphatic chain [1,2], and exemplified by the antituberculosis rifamycin [3], antitumor maytansine [4,5] and the Hsp90 inhibitor geldanamycin [6]. These macrolactams are constructed by the multidomain modular type I PKSs (polyketide synthases) using 3-amino-5-hydroxybenzoic acid (AHBA) as the starter unit [7], followed by various post-PKS modifications.

Rifamycins were first reported in 1957 from *Amycolatopsis mediterranei* S699 [8,9,10]. They have good antibacterial activity against G^+^ bacteria and some G^−^ bacteria [11]. The semi-synthetic rifamycin derivatives such as rifampicin are clinically used for the treatment of tuberculosis, adhesion and leprosy infection caused by *Staphylococcus* and other G^+^ bacteria [12,13]. In recent years, due to its widespread use, pathogens, especially *Mycobacterium tuberculosis*, have gradually developed resistance to rifampicin [14,15]. In order to increase the structural diversity of rifamycins, the mechanism of rifamycin biosynthesis has been continuously studied [16,17,18].

**Figure 1:**
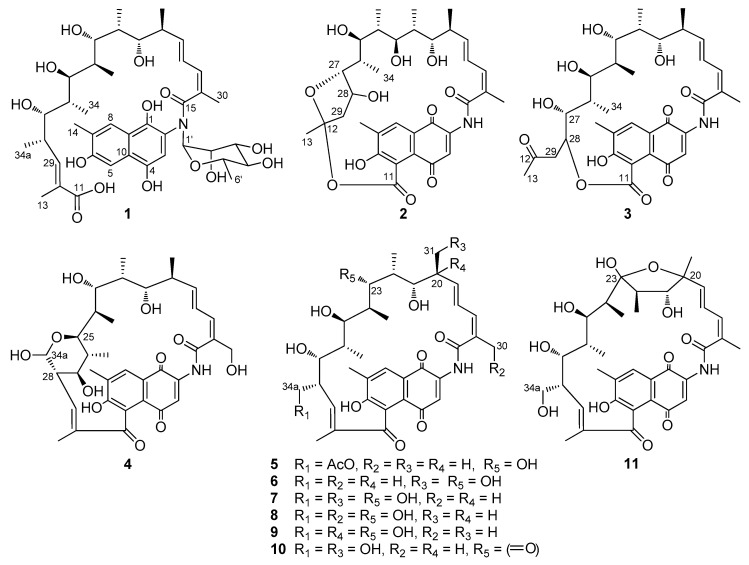
Structures of new compounds **1**–**11**.

The *rifT* gene, located upstream of the PKS genes in the rifamycin biosynthetic gene cluster of *A. mediterranei* S699, was proposed to encode a dehydrogenase [19] on the basis of bioinformatics analysis. In this study, the mutant strain *A. mediterranei* S699 *ΔrifT* was constructed by deleting the *rifT* gene (Supplementary Figures S1–S7). Systematical isolation of the fermentation products of the mutant strain afforded eleven new 8-deoxy-rifamycin derivatives (**1**–**11**) (Figure 1) and seven known analogues (**12**–**18**) (Supplementary Figure S8). Herein, the isolation, structure elucidation and bioactivity of these eighteen compounds are reported.

## 2. Materials and Methods

### 2.1. Strains and Plasmids

*Amycolatopsis mediterranei* S699 strain was a gift from Prof. Linquan Bai at Shanghai Jiaotong University. *A. mediterranei* S699 *ΔrifT* strain was constructed by deleting the *rifT* gene which was predicted to be responsible for the dehydrogenation of putative proansamycin X [20,21,22]. The strain was initially propagated on ISP2 agar medium (4 g/L yeast extract, 10 g/L malt extract, 4 g/L glucose and 20 g/L agar). Then, a single colony of each strain was inoculated in 50 mL of ISP2 medium with 8 g of glass beads (Ø 3 ± 0.3 mm) in a 250 mL baffled flask and cultivated at 28 °C in a shaking incubator at 200 rpm. *Escherichia coli* DH5α strain was used as the general cloning host and grown in Luria-Bertani (LB) medium. Cell stocks were prepared with 20% glycerol and stored at −80 °C. Apramycin was added into media as an antibiotic at a final concentration of 50 μg/mL for all strains in this study.

Suicide vector pOJ260 [23] (containing *aac (3) IV, oriT, rep^PUC^, lacZ*) was used throughout the study for in-frame gene inactivation. The integrating vector pSET152 (containing *aac (3) IV, oriT (RK2), ori (pUC18), int (φC31), attP (φC31), lacZα*) was used for gene expression in the *ΔrifT::rifT* strain.

### 2.2. DNA Manipulation

#### 2.2.1. Gene Knock-Out

The knock-out plasmid for the *rifT* gene was generated as the following steps. The ~2 kb upstream and downstream homologous arms of the target genes were amplified by polymerase chain reaction (PCR) using *A. mediterranei* S699 genomic DNA as a template, respectively. Purified PCR fragments were inserted into the linearized pOJ260 by Gibson assembly [24]. The assembled product was then transformed into 100 μL DH5α-competent cells. Positive clones were identified by restriction enzyme digestion (Supplementary Figure S1) and DNA sequencing. The knock-out plasmid was propagated in DH5α and transformed into *A. mediterranei* S699 competent cells by electroporation, as described by Ding et al. [25]. The apramycin-resistant recombinants resulting from the homologous recombination between the knock-out plasmid and genomic DNA of *A. mediterranei* S699 were selected (Supplementary Figure S2) and transferred to ISP2 agar for several rounds of nonselective growth. Apramycin-sensitive recombinants derived from double-crossover recombination were screened, from which the targeted gene knockout mutant was verified by PCR (Supplementary Figure S3). The specific process is shown in the Supplementary Information (Supplementary Figure S4).

#### 2.2.2. Gene Complementation

The integrating vector pSET152 [23] was used for gene complementation in the *A. mediterranei* S699 *ΔrifT* strain. Synthesized *rifK* promoter fragment was digested with NdeI and XbaI, and inserted into XbaI-pretreated pSET152 vector to yield pSET152-*rifK*p. The *rifT* gene was amplified by PCR using the genomic DNA of *A. mediterranei* S699 as a template. The NdeI/EcoRI *rifT* PCR fragment was inserted into the downstream of the *rifK*p promoter in pSET152. Positive clones were identified by restriction enzyme digestion and DNA sequencing. The resultant plasmid pSET152-*rifK*p-*rifT* was transformed into *A. mediterranei* S699 *ΔrifT*-competent cells by electroporation to obtain the *rifT* gene complementation mutant.

### 2.3. Detection and Analysis of the Metabolites in Mutants

For rifamycins production, *A. mediterranei* S699 mutants were inoculated on ISP2 agar media (100 mL) and cultivated for 7 days at 28 °C. The culture was diced and extracted overnight with EtOAc/MeOH (4:1, *v/v*) at room temperature. The concentrated crude extract was dissolved in 1 mL MeOH, and analyzed by high-pressure liquid chromatography (HPLC; Agilent 1200). Chromatographic conditions were as follows: solvents: (A) water, and (B) CH_3_CN, samples were eluted with a linear gradient from 20% to 35% B in the first 5 min, increased to 55% B at 19 min, to 65% B at 20 min, to 100% B at 23 min, followed by 4 min with 100% B, flow rate was 1 mL/min, and UV detection at 254 nm. In order to specify compound peaks, the concentrated crude extract was analyzed by liquid chromatography–electrospray ionization–high-resolution mass spectrometry (LC-ESI-HRMS; Finnigan). Chromatographic conditions were as follows: solvents: (A) water, and (B) CH_3_CN, samples were eluted with a linear gradient from 30% to 45% B in the first 10 min, increased to 65% B at 15 min, to 90% B at 19 min, followed by 5 min with 100% B, flow rate: 1 mL/min, and UV detection at 254 nm.

### 2.4. General Experimental Procedures

The NMR spectra were recorded on Bruker 400 MHz and/or AVANCE 600 MHz NMR spectrometers with tetramethylsilane (TMS) as an internal standard. HRESIMS analyses were carried out on a LTQ-Orbitrap XL (Thermo Scientific, Waltham, MA, USA). Silica gel GF_254_ for thin-layer chromatography (TLC) was purchased from Qingdao Marine Chemical Ltd. (Qingdao, China). Column chromatography (CC) was performed on reversed-phase (RP) C_18_ silica gel (Merck, Darmstadt, Germany) CC and Sephadex LH-20 (GE Amersham Biosciences, Piscataway, NJ, USA) stationary phases. High-performance liquid chromatography (HPLC) was performed on an Agilent 1200. Semi-preparative HPLC was performed on a Waters 1525 Binary HPLC Pump (Agilent Eclipse XDB-C_18_, 5 μm, 9.4 × 250 mm) and a Waters 996 Photodiode Array Detector. Compounds were visualized under UV light and by Iodine vapor. Optical rotations were measured on an Auton Paar MCP200 Automatic Polarimeter. IR spectra (KBr) were obtained on a Nicolet 6700 FT-IR spectrometer.

### 2.5. Extraction and Isolation

The 15 L culture was diced and extracted overnight with EtOAc/MeOH (4:1, *v*/*v*) at room temperature three times. The crude extract was partitioned between H_2_O and EtOAc (1:1, *v*/*v*) until the H_2_O layer was colorless. The EtOAc extract was partitioned between 95% aqueous MeOH and petroleum ether (PE) to afford the defatted MeOH extract. The MeOH extract was fractionated by medium-pressure liquid chromatography (MPLC) over RP C_18_ silica gel (130 g) eluted with gradient aqueous CH_3_CN (30%, 50%, 70% and 100% CH_3_CN, 500 mL each) to give six fractions (Fr.), A–F.

Fr. B (1.05 g) was purified by MPLC over RP C_18_ silica gel (60 g) eluted with gradient aqueous CH_3_CN (30%, 50%, 70% and 100% CH_3_CN, 200 mL each) to afford Fr. B1 and B2. Fr. B2 (90 mg) was purified by HPLC (4 mL/min; UV 274 nm) eluted with 40% CH_3_CN to afford 1 (*t*_R_ 3.5 min, 17.5 mg), 4 (*t*_R_ 3.5 min, 11.5 mg) and 7 (*t*_R_ 5.9 min, 4.4 mg). Fr.C (0.97 g) was purified by column chromatography (CC) over Sephadex LH-20 eluted with MeOH to afford Fr. C1–C4. Fr. C2 (60 mg) was purified by HPLC (4 mL/min; UV 274 nm) eluted with 40% CH_3_CN to afford 6 (*t*_R_ 4.4 min, 6.6 mg). Fr. C3 (167 mg) was subjected to MPLC over RP C_18_ silica gel (30 g) eluted with gradient aqueous CH_3_CN (30%, 35%, 40%, 45%, 50% and 100% CH_3_CN, 100 mL each) to afford Fr. C3a and C3b. Fr. C3a (80 mg) was purified by HPLC (4 mL/min; UV 274 nm) eluted with 55% CH_3_CN to afford 8 (*t*_R_ 4.5 min, 9.9 mg) and 9 (*t*_R_ 4.5 min, 17 mg). By the same method, compounds **14** (4.1 mg) and **15** (3.6 mg) were obtained from Fr. C3b, and **2** (6.0 mg) and **10** (9.0 mg) were purified from Fr. C3b by HPLC (4 mL/min; UV 274 nm) eluted with 35–55% CH_3_CN. Fr. D, Fr. E1, Fr. E2 and Fr. E3 gave compounds **13** (64 mg), **3** (4.4 mg), **5** (8.6 mg), **12** (20 mg), **16** (4.7 mg), **17** (15.6 mg), **18** (10 mg) and **11** (20 mg), respectively.

Compound **1**: dark brown powder; [α]^20^_*D*_ = +9.8 (*c* 0.13, MeOH); UV (MeOH) *λ*_max_ (logε) 216 (4.43), 262 (4.22), 315 (3.81) nm; IR (KBr) *ν*_max_ 3347, 2969, 2932, 2256, 2128, 1657, 1454, 1232, 1025, 997, 825 cm^−1^; ^1^H NMR data, Table 1; ^13^C NMR data, Table 2; HRESIMS: *m/z* 790.4012 [M + H]^+^ (calculated for C_41_H_60_NO_14_^+^, 790.3969), and 812.3817 [M + Na]^+^ (calculated for C_41_H_59_NO_14_Na^+^, 812.3833).

Compound **2**: yellow powder; [α]^20^_*D*_ = +17.3 (*c* 0.15, MeOH); UV (MeOH) *λ*_max_ (logε) 212 (4.39), 272 (4.32), 311 (4.13) nm; IR (KBr) *ν*_max_ 3366, 2969, 2931, 1655, 1498, 1339, 1123, 1061, 976 cm^−1^; ^1^H NMR data, Table 1; ^13^C NMR data, Table 2; HRESIMS: *m/z* 642.2920 [M + H]^+^ (calculated for C_34_H_44_NO_11_^+^, 642.2870), and 664.2734 [M + Na]^+^ (calculated for C_34_H_43_NO_11_Na^+^, 664.2734).

Compound **3**: yellow powder; [α]^20^_*D*_ = +19.7 (*c* 0.12, MeOH); UV (MeOH) *λ*_max_ (logε) 211 (4.37), 277 (4.33), 307 (4.15) nm; IR (KBr) *ν*_max_ 3362, 2970, 2936, 2878, 2359, 1655, 1500, 1332, 1210, 1109, 979 cm^−1^; ^1^H NMR data, Table 1; ^13^C NMR data, Table 2; HRESIMS: *m/z* 664.2736 [M + Na]^+^ (calculated for C_34_H_43_NO_11_Na^+^, 664.2734).

Compound **4**: tawny powder; [α]^20^_*D*_ = −268.5 (*c* 0.10, MeOH); UV (MeOH) *λ*_max_ (logε) 211 (4.37), 275 (4.32), 310 (4.05) nm; IR (KBr) *ν*_max_ 3362, 2968, 2931, 1657, 1499, 1335, 1201, 1110, 977 cm^−1^; ^1^H NMR data, Table 1; ^13^C NMR data, Table 2; HRESIMS: *m/z* 654.2924 [M + H]^+^ (calculated for C_35_H_44_NO_11_^+^, 654.2870), and 676.2737 [M + Na]^+^ (calculated for C_35_H_43_NO_11_Na^+^, 676.2734).

Compound **5**: brown powder; [α]^20^_*D*_ = +65.7 (*c* 0.12, MeOH); UV (MeOH) *λ*_max_ (logε) 225 (4.29), 271 (4.16), 309 (3.95) nm; IR (KBr) *ν*_max_ 3359, 2968, 2931, 1658, 1494, 1339, 1261, 1110, 975 cm^−1^; ^1^H NMR data, Table 1; ^13^C NMR data, Table 2; HRESIMS: *m/z* 682.3227 [M + H]^+^ (calculated for C_37_H_48_NO_11_^+^, 682.3183), and 704.3047 [M + Na]^+^ (calculated for C_37_H_47_NO_11_Na^+^, 704.3047).

Compound **6**: yellow powder; [α]^20^_*D*_ = +81.5 (*c* 0.10, MeOH); UV (MeOH) *λ*_max_ (logε) 225 (4.36), 275 (4.26), 307 (3.99) nm; IR (KBr) *ν*_max_ 3361, 2969, 2933, 1656, 1495, 1340, 1153, 1062, 977 cm^−1^; ^1^H NMR data, Table 1; ^13^C NMR data, Table 2; HRESIMS: *m/z* 640.3118 [M + H]^+^ (calculated for C_35_H_46_NO_10_^+^, 640.3077), and 662.2936 [M + Na]^+^ (calculated for C_35_H_45_NO_10_Na^+^, 662.2941).

Compound **7**: brown powder; [α]^20^_*D*_ = +124.7 (*c* 0.11, MeOH); UV (MeOH) *λ*_max_ (logε) 210 (4.51), 263 (4.30), 310 (4.13) nm; IR (KBr) *ν*_max_ 3355, 2966, 2936, 1655, 1494, 1339, 1201, 1052, 968 cm^−1^; ^13^C NMR data, Table 2; ^1^H NMR data, Table 3; HRESIMS: *m/z* 656.3066 [M + H]^+^ (calculated for C_35_H_46_NO_11_^+^, 656.3026), and 678.2884 [M + Na]^+^ (calculated for C_35_H_45_NO_11_Na^+^, 678.2890).

Compound **8**: brown powder; [α]^20^_*D*_ = +168.4 (*c* 0.20, MeOH); UV (MeOH) *λ*_max_ (logε) 212 (4.40), 273 (4.31), 313 (4.11) nm; IR (KBr) *ν*_max_ 3361, 2970, 2934, 1657, 1495, 1340, 1223, 1054, 975 cm^−1^; ^13^C NMR data, Table 2; ^1^H NMR data, Table 3; HRESIMS: *m/z* 656.3076 [M + H]^+^ (calculated for C_35_H_46_NO_11_^+^, 656.3026), and 678.2891 [M + Na]^+^ (calculated for C_35_H_45_NO_11_Na^+^, 678.2890).

Compound **9**: yellow powder; [α]^20^_*D*_ = +146.8 (*c* 0.15, MeOH); UV (MeOH) *λ*_max_ (logε) 229 (4.38), 270 (4.26), 308 (4.08) nm; IR (KBr) *ν*_max_ 3370, 2972, 2936, 1656, 1494, 1339, 1221, 1063, 974 cm^−1^; ^13^C NMR data, Table 2; ^1^H NMR data, Table 3; HRESIMS: *m/z* 656.3067 [M + H]^+^ (calculated for C_35_H_46_NO_11_^+^, 656.3026), and 678.2884 [M + Na]^+^ (calculated for C_35_H_45_NO_11_Na^+^, 678.2890).

Compound **10**: brown powder; [α]^20^_*D*_ = +161.1 (*c* 0.20, MeOH); UV (MeOH) *λ*_max_ (logε) 226 (4.43), 310 (4.07) nm; IR (KBr) *ν*_max_ 3359, 2969, 2933, 1656, 1492, 1339, 1219, 1051, 970 cm^−1^; ^1^H NMR data, Table 3; ^13^C NMR data, Table 2; HRESIMS: *m/z* 676.2731 [M + Na]^+^ (calculated for C_35_H_43_NO_11_Na^+^, 676.2734).

Compound **11**: red-brown powder; [α]^20^_*D*_ = −3620.7 (c 0.20, MeOH); UV (MeOH) *λ*_max_ (logε) 226 (4.39), 310 (4.06) nm; IR (KBr) *ν*_max_ 3345, 2973, 2937, 1644, 1492, 1341, 1214, 1071, 971 cm^−1^; ^1^H NMR data, Table 3; ^13^C NMR data, Table 2; HRESIMS: *m/z* 654.2925 [M + H]^+^ (calculated for C_35_H_44_NO_11_^+^, 654.2870), and 676.2737 [M + Na]^+^ (calculated for C_35_H_43_NO_11_Na^+^, 676.2734).

### 2.6. Antimicrobial Assay

Compounds **1**–**18** were assayed for their antimicrobial activity with the paper disc diffusion assay against *Staphylococcus aureus* ATCC 25923, *Mycobacterium smegmatis* mc^2^ 155, *Pseudomonas aeruginosa* PA01 and *Proteusbacillus vulgaris* CPCC 160013 purchased from the China Center of Industrial Culture Collection (Beijing, China) [26]. Kanamycin and Rifampicin were used as positive controls. The tested compounds (40 μg each) were absorbed onto individual paper disks (Ø 6 mm) and placed on the surface of the agar. The assay plates were incubated for 24 h at 37 °C and examined for the presence of inhibitory zones.

The microbroth dilution method [27] was applied to determine the MIC value of active compounds against the growth of *Staphylococcus aureus* ATCC 25923. Kanamycin was used as a positive control. Microorganisms were cultured in LB (tryptone 10 g, yeast extract 5 g, NaCl 10 g, ddH_2_O 1000 mL, pH 7.2) media in 96-well plates at a concentration of 1 × 10^6^ CFU/mL, and the MIC values were obtained after incubating for 12 h at 37 °C with the tested compounds.

### 2.7. Cytotoxicity Assay

The in vitro antiproliferative activity against KG1 cells was measured: the cells were purchased from Cell Bank of the Institute of Biochemistry and Cell Biology, China Academy of Sciences (Shanghai, China), and cultured in RPMI 1640 (Roswell Park Memorial Institute 1640) media with 10% fetal bovine serum (Biological Industries), incubating at 37 °C in a humidified atmosphere containing 5% CO_2_. Cell-grown inhibition was determined using the Cell Counting Kit-8 (CCK-8) (Bimake, Houston, TX, USA) according to the manufacturer’s instructions [28]. Briefly, cells were seeded in 96-well plates at 7 × 10^3^ cells/well and treated with different concentrations of compounds **1**–**18** for the indicated 48 h. Cytosporone B (VP16) was used as the positive control. Then, 10 µL CCK-8 was added to each well and incubated for another 4 h. The absorbance was read at 480 nm by Spark 30086376 (TECAN, Austria). Growth inhibition (%) was calculated at each concentration and the IC_50_ was calculated by software Prism 7 (GraphPad Software, Inc., San Diego, CA, USA).

### 2.8. Anti-Type III Secretion System (T3SS) Assay

*Salmonella enterica* is the major cause of foodborne illness and typhoid fever [29,30], and uses a type III secretion system (T3SS) to translocate the virulence factors into host cells [31]. These virulence factors include specific effector proteins encoded by the *S. enterica* pathogenicity island 1 (SPI-1) [32]. T3SSs are highly conserved among Gram-negative bacteria [33]. Compounds **1**–**18** were assayed for their anti-T3SS activity of *S. enterica* Typhimurium UK-1 χ8956 in vitro, as previously described in our laboratory [34,35,36]. *S. enterica* Typhimurium UK-1 χ8956 was cultured in the LB media (tryptone 10 g, yeast extract 5 g, NaCl 10 g, ddH_2_O 1000 mL, pH 7.2) supplemented with 0.2% L-arabinose at 37 °C in the presence of a solvent control or the tested compounds at the final concentration of 100 μM [37], respectively. Cytosporone B (Csn-B) was used as the positive control [38]. *Salmonella enterica* Typhimurium UK-1 χ8956 was a gift from Roy Curtiss III (School of Life Sciences, Arizona State University) [37].

## 3. Results

**Figure 2:**
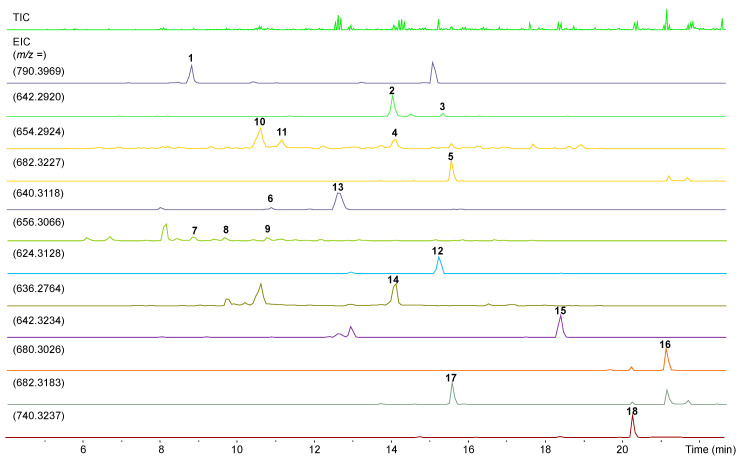
Designation of compound peaks of the *A. mediterranei* S699 *ΔrifT* mutant by LC-ESI-HRMS (liquid chromatography-electrospray ionization-high-resolution mass spectrometry).

The *A. mediterranei* S699 *ΔrifT* mutant shows different morphological characteristics compared with that of the wild-type strain. After analysis of the fermentation products cultivated for 7 days on ISP2 agar media at 28 °C by HPLC and LC-ESI-HRMS (liquid chromatography-electrospray ionization-high-resolution mass spectrometry), the *ΔrifT* mutant exhibited a completely different metabolic profile from that of the wild-type strain (Figure 2). In addition, a *rifT* gene complementation plasmid was constructed (Supplementary Figure S5) and introduced into the *ΔrifT* mutant to get the complementation mutant *ΔrifT::rifT* (Supplementary Figure S6). HPLC analysis indicated that the metabolites of the *ΔrifT::rifT* strain were almost identical to that of the wild-type one (Supplementary Figure S7), which definitely eliminated the polar effect caused by genetic manipulation.

In order to explore the products accumulated by the *A. mediterranei* S699 *ΔrifT* mutant, 15 L fermentation was carried out and the fermented agar cakes were diced and extracted. The extract was subjected to column chromatography over Sephadex LH-20, MPLC over RP C_18_ silica gel, and finally, HPLC, to yield compounds **1**–**18**.

**Figure 3:**
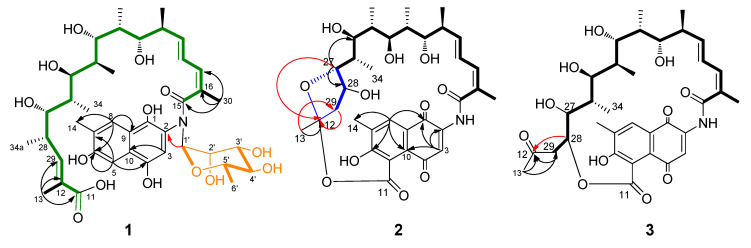
Selected HMBC (^1^H detected heteronuclear multiple bond correlation) (→) and ^1^H-^1^H COSY (correlation spectroscopy) (**—**) correlations of compounds **1**, **2** and **3**.

Compound **1** was determined to have the molecular formula C_41_H_59_NO_14_ on the basis of the quasi molecular ion peaks at HRESIMS *m/z* 790.4012 [M + H]^+^ and 812.3817 [M + Na]^+^ (Supplementary Figure S9). The ^1^H and ^13^C NMR spectroscopic data (Table 1 and Table 2) (Supplementary Figures S10–S14) indicated that **1** had a structural skeleton of rifamycin, but the signals for a deoxyhexapyranose were also clearly observed. The presence of a naphthaquinone chromophore was indicated by the HMBC correlations from H-3 (*δ*_H_ 7.30) to C-9 (*δ*_C_ 137.1) and C-10 (*δ*_C_ 122.6), and from H-5 (*δ*_H_ 7.34) to C-4 (*δ*_C_ 147.7), C-6 (*δ*_C_ 153.4), C-7 (*δ*_C_ 127.4) and C-8 (*δ*_C_ 123.1), as well as from H-8 (*δ*_H_ 7.68) to C-6 (*δ*_C_ 153.4), C-8 (*δ*_C_ 123.1), C-9 (*δ*_C_ 137.1) and C-13 (*δ*_C_ 12.5) (Supplementary Table S1 and Figure 3). The 24-carbon fragment from C-15 to C-11 (*δ*_C_ 169.3) was established on the basis of ^1^H-^1^H COSY correlations along with the HMBC correlations from the Me-13, Me-30, Me-31, Me-32, Me-33, Me-34 and Me-34a to the corresponding carbons (Supplementary Table S1 and green in Figure 3). The presence of deoxyhexapyranose moiety (Figure 3, orange), was determined based on the ^1^H-^1^H COSY correlations of H-1′ (*δ*_H_ 4.82) with H-2′ (*δ*_H_ 4.09), H-5′ (*δ*_H_ 3.96) with H-4′ (*δ*_H_ 3.33) and H-6′ (*δ*_H_ 1.19), along with the HMBC correction from H-1′ to C-2′ (*δ*_C_ 70.8), from H-4′ to C-2′ (*δ*_C_ 70.8) and C-6′ (*δ*_C_ 18.0), and from H-6′ to C-1′ (*δ*_C_ 105.1) and C-2′ (*δ*_C_ 70.8). The deoxyhexapyranose moiety attached to the amide nitrogen of ansamycin was determined based on the HMBC correlations from H-1′ to C-2 (*δ*_C_ 136.9). In order to determine its stereochemistry, the sugar was purified from the spontaneous hydrolysis products of **1** (Supplementary Figures S15 and S16). The ^1^H NMR spectroscopic data of the sugar was completely consistent with that of authentic α-*L*-rhamnose (Supplementary Figure S17). The [α]^20^_*D*_ = +8.3 (*c* 0.13, MeOH) was close to the optical rotation [α]^20^_*D*_ = +7.3~8.0 of *α-L-*rhamnose. The *ansa* chain was suggested to occur as retro-Claisen cleavage between C-5 and C-11 in consideration of the remaining aromatic hydrogen H-5 (*δ*_H_ 7.34) and chemical shift of C-11 (*δ*_C_ 169.3) (Supplementary Table S1 and Figure 3). The stereochemistry of C-20 to C-28 was assumed to be same as that of protorifamycin I [39] on the basis of biosynthetic logic. Thus, compound **1** was determined to be *N*-α-*L*-rhamnosyl proansamycin B-M1, a novel rifamycin amide *N*-rhamnoside, named rifamycinoside C.

The molecular formula of **2** was determined to be C_34_H_43_NO_11_ by the HRESIMS quasi molecular ion peaks at *m/z* 642.2876 [M + H]^+^ and 664.2736 [M + Na]^+^ (Supplementary Figure S18). The NMR data (Supplementary Figures S19–S23) comparison with those of proansamycin B [40] revealed that the structure of **2** was different in the C-11/C-12 cleavage and decarboxylation of C-34a. The ether linkage between C-11 (*δ*_C_ nda) and C-12 (*δ*_C_ 107.5) and the formation of a five-membered ring (blue, Figure 3) between C-12 and C-27 (*δ*_C_ 89.2) was confirmed by decarboxylation and hydroxylation of C-28 (*δ*_C_ 71.2) in **2**, and supported by the HMBC correlations of H-13 (*δ*_H_ 1.44) to C-12 (*δ*_C_ 107.5) and C-29 (*δ*_C_ 46.6), H-27 (δH 3.98) to C-12 and C-28, the ^1^H-^1^H COSY of H-28 (*δ*_H_ 4.39) with H-29 (*δ*_H_ 2.48,1.75) and the degrees of desaturation (Supplementary Table S2 and Figure 3). Thus, compound **2** was determined to be 11,12-*seco*-28-desmethyl-28-hydroxyprotorifamycin I 12,27-epoxy-11-carboxy-12-ester.

The molecular formula of **3** was determined to be C_34_H_43_NO_11_ on the basis of HRESIMS ions at *m/z* 642.2920 [M + H]^+^ and 664.2734 [M + Na]^+^ (Supplementary Figure S24). The NMR spectra of **3** (Supplementary Figures S25–S29) were similar to those of **2**, except that the *ansa* chain was suggested to connect to C-5 of the naphthoquinoid via an ester bond between C-11 and C-28 on the basis of the HMBC correlations from H-28 (*δ*_H_ 3.96) with C-12 (*δ*_C_ 211.0), from H-29 (*δ*_H_ 2.76, 2.65) with C-12 and C-28 (*δ*_C_ 81.5) as well as the remaining degrees of unsaturation and the molecular formula (Supplementary Table S3 and Figure 3). Based on the NMR data comparison with those of rifamycinoside A [41], **3** was most likely the aglycone moiety of rifamycinoside A, both of them occurred at decarboxylation of C-34a and C-11/12 cleavage of *ansa* chain. Thus, compound **3** was determined to be 11,12-*seco*-28-desmethyl-28-hydroxyprotorifamycin I 11-carboxy-28-ester.

Compound **4** was confirmed to have the molecular formula of C_35_H_43_NO_11_ on the basis of the HRESIMS ion peaks at *m/z* 654.2924 [M + H]^+^ and 676.2737 [M + Na]^+^ (Supplementary Figure S30). Detailed analysis of NMR spectroscopic data of **4** (Supplementary Table S4) demonstrated that the structure of **4** was similar to protorifamycin I, except that hydroxylation of Me-30 and Me-34a oxidized to an aldehyde group and formation of hemiacetal with a C-25 hydroxyl group, which was supported by ^1^H NMR of H-30 (*δ*_H_ 4.46, 4.24), H-34a (*δ*_H_ 5.12) and HMBC correlation of H-34a and C-25 (*δ*_C_ 73.7) (Table 1 and Table 3) (Supplementary Figures S31–S35). Thus, **4** was determined as a new rifamycin hemiacetal derivative, named 30-hydroxy-protorifamycin I-hemiacetal.

The molecular formula of compound **5** was elucidated as C_37_H_47_NO_11_ (Supplementary Figure S36). A close NMR comparison with that of protorifamycin I [39] revealed that the evident difference was one or more acetyl signals coupling with H-34a, indicating the acetylation of H-34a in **5**, which was confirmed by the HMBC correlations of H-34a (*δ*_H_ 4.01, 4.00) with C-35 (*δ*_C_ 173.0) and H-36 (*δ*_H_ 2.03) with C-35 (Supplementary Table S5 and Figures S37–S41). Thus, **5** was elucidated as 34a-acetyl-protorifamycin I.

Compound **6** was confirmed to have the molecular formula of C_35_H_45_NO_10_ on the basis of the HRESIMS at *m/z* 640.3118 [M + H]^+^ and 662.2936 [M + Na]^+^ (Supplementary Figure S42), the same as that of protorifamycin I. The down-field chemical shifts of C-31 (*δ*_H_ 3.52, 3.53, *δ*_C_ 63.9) and up-field chemical shifts of C-34a (*δ*_H_ 1.06, *δ*_C_ 20.0) revealed the hydroxylation of C-31 (Table 1 and Table 2) (Supplementary Table S6 and Supplementary Figures S43–S47). Thus, the structure of compound **6** was determined and named as 31-hydroxyproansamycin B.

Compounds **7**, **8** and **9** were determined to have the same molecular formula of C_35_H_45_NO_11_ on the basis of HRESIMS data (Supplementary Figures S48–S50), revealing one more oxygen atom than that of protorifamycin I. NMR comparison determined **7** to be 31-hydroxyprotorifamycin I supported by the chemical shifts of C-31 (*δ*_H_ 3.54, *δ*_C_ 63.5) (Supplementary Table S7 and Supplementary Figures S51–S55), **8** to be 30-hydroxyprotorifamycin I with chemical shifts of C-30 (*δ*_H_ 4.23, 4.36, *δ*_C_ 65.4) (Supplementary Table S8 and Supplementary Figures S56–S60) and **9** to be 20-hydroxyprotorifamycin I with chemical shifts of C-30 (*δ*_C_ 76.9) (Supplementary Table S9 and Supplementary Figures S61–S65).

Compounds **10** and **11** had the same molecular formula of C_35_H_43_NO_11_, as indicated by their HRESIMS data (Supplementary Figures S66 and S67). Compound **10** was determined to be 31-hydroxy-23-protorifamycin I-acetal on the basis of chemical shifts of C-31 (*δ*_H_ 4.37, 4.23, *δ*_C_ 65.1) and C-23 (*δ*_C_ 211.3) (Table 2 and Table 3) (Supplementary Table S10 and Supplementary Figures S68–S72), and **11** was elucidated to be 20-hydroxy-20,23-protorifamycin I-hemiacetal, which was supported by ^1^H NMR of H-31 (*δ*_H_ 1.31), ^13^C NMR of C-20 (*δ*_C_ 82.3) and C-23 (*δ*_C_ 106.8) (Table 2 and Table 3) (Supplementary Table S11 and Supplementary Figures S73–S77).

Based on the HRESIMS data, NMR spectroscopic analysis and the comparison with the reported NMR data, compounds **12**–**19** were determined to be proansamycin B (**12**) [40], protorifamycin I (**13**) [42], protorifamycin I-lactone (**14**) [40], prorifamycin B-M1 (**15**) [40], 8-deoxy-rifamycin S (**16**) [43], 8-deoxy-rifamycin SV (**17**) [43], 8-deoxy-rifamycin B (**18**) [43] and rifamycin W (**19**) [44], respectively.

Compounds **1**–**18** were assayed for their antimicrobial activity against *Staphylococcus aureus* ATCC 25923, *Mycobacterium smegmatis* mc^2^ 155, *Pseudomonas aeruginosa* PA01 and *Proteusbacillus vulgaris* CPCC160013 through the paper disc diffusion method [26], respectively. The results (Supplementary Figure S78) showed that **2**, **3**, **5**, **6**, **13** and **15** exhibited inhibitory activity against *S*. *aureus* ATCC 25923, and **2** and **6** had modest inhibitory against *P. vulgaris* CPCC160013. Compounds **2**, **3**, **5**, **6**, **13** and **15** were further tested for their antibacterial activity against *S*. *aureus* ATCC 25923 using the microbroth dilution method [27], and their MIC values were determined to be 10, 20, 20, 20, 40 and 20 μg/mL, respectively (Supplementary Tables S12 and S13).

Compounds **1**–**18** were evaluated for their antiproliferative activity against KG1 cells using the Cell Counting Kit-8 (CCK-8) (Bimake, USA) and etoposide (VP-16) was used as a positive control. Compounds **14**, **15**, **16**, **17** and **18** showed potent activity in inhibiting the proliferation of KG1 cells with IC_50_ values of 14.9, 44.8, 2.2, 18.7 and 8.1 μM, respectively (Supplementary Table S14). The cytotoxicity of **16** and **18** was close to that of the positive control VP16 (IC_50_: 1.5 μM). Compounds **1**−**18** were further assayed for their activities of inhibiting the T3SS of *Salmonella enterica* Typhimurium UK-1 χ8956. Only **14** and **17** showed modest activity (Supplementary Figure S79).

## 4. Discussion

The biosynthesis of rifamycins has been extensively studied ever since the discovery of its biosynthetic gene cluster, and it can be divided into three stages: the first stage is the synthesis of the starting unit AHBA (3-amino-5-hydroxybenzoic acid) [45,46], the second stage is the extension of rifamycin polyketide [16,20,21] and the third stage is the rifamycin post-PKS modification [17]. The first two stages have been clearly studied, however, the formation process from the putative proansamycin X to rifamycin W, an important intermediate, is still unclear. According to previous research, there may be a C7/C8 dehydrogenation reaction in this progress. To investigate the dehydrogenation of proansamycin X, the mutant strain *Amycolatopsis mediterranei* S699 *ΔrifT* was constructed by deleting the *rifT* gene (putative NADH-dependent dehydrogenase gene).

**Figure 4:**
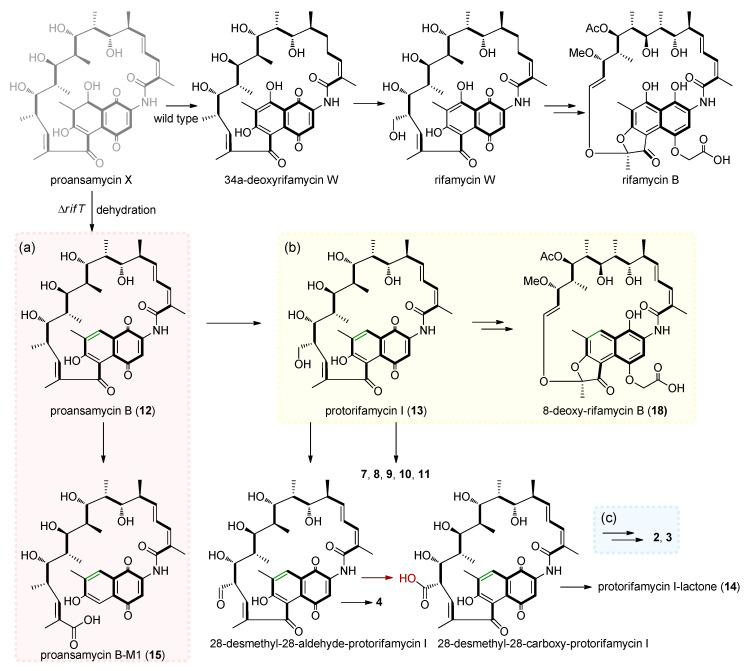
Three cleavage patterns of the *ansa* chain in the biosynthesis of 8-deoxy-rifamycins. (**a**) 5,11 retro-Claisen cleavage lead to proansamycin B-M1 (**15**). (**b**) 12,19 double bond cleavage lead to 8-deoxy-rifamycin B (**18**). (**c**) 11,12 Baeyer-Villiger oxidation cleavage to **2** and **3**.

The structures revealed that all eighteen compounds isolated from the *ΔrifT* mutant strain had undergone deoxygenation at C-8. However, we could not successfully obtain proansamycin X, which was possibly due to its instability of a hydroxyl group at C-8 within the conjugated system from C-1 to C-10. When the putative *rifT* gene-dependent dehydrogenation in rifamycin B biosynthetic route ceased, accumulated proansamycin X tended to undergo dehydration at C-7/C-8 to form a stable naphthalene ring and transformed to proansamycin B (Figure 4a), which can be subjected to sequent *ansa* polyketide chain post-PKS modifications to produce a series of 8-deoxy-rifamycin derivatives. Moreover, the metabolites of the complementation mutant *ΔrifT::rifT* were identical to that of the wild-type strain (Supplementary Figure S7), indicating that the *rifT* gene was involved in the biosynthesis of rifamycins.

The biosynthesis pathway of 8-deoxy-rifamycins demonstrated diverse cleavage patterns of *ansa* polyketide backbone, including 5,11 retro-Claisen cleavage, just like that observed in *ansa* biosynthesis of divergolides R and S [47], hygrocins I and J [48] and microansamycins G-I [49], which lead to protorifamycin I-M1 and proansamycin B-M1 (**15**) (Figure 4a), 12,19 double-bond cleavage and skeleton rearrangement lead to 8-deoxy-rifamycin B (**18**) (Figure 4b) and a novel 11,12-cleavage carried out by a typical Baeyer-Villiger oxidation and intramolecular transesterification formed **2** and **3** (Figure 4c) [41]. Compounds **5**, **6**, **7**, **8**, **9**, **10** and **11** oxygenated at C-20, C-23, C-30 and C-31 suggested that the *ansa* chain is prone to be oxidized in the *ΔrifT* strain during fermentation. In addition, the oxidation process of C-34a from compounds **4** and **14** to compounds **2** and **3** represented that the oxidation of C-34a alcohol to the carboxyl group may occur before the 12,29-olefinic bond and 11,12-oxygen insertion cleavage.

## 5. Conclusions

In summary, the results of in vivo gene inactivation and complementation indicated that the *rifT* gene is involved in the biosynthesis of rifamycins, and the 8-deoxy-rifamycin proansamycin B could undergo post-PKS modifications similar to that of its 8-hydroxyl analogue 34a-deoxyrifamycin W. Accordingly, eleven new derivatives of 8-deoxy-rifamycin were isolated and characterized, including a novel amide *N*-glycoside of *seco*-rifamycin **1**, **2** and **3**, which featured the third *ansa* chain cleavage pattern of rifamycins [41]. Compounds **2**, **3**, **5**, **6**, **13** and **15** exhibited antibacterial activity against *Staphylococcus aureus*. Compounds **14**, **15**, **16**, **17** and **18** showed potent antiproliferative activity against KG1 cells, respectively.

## Figures and Tables

**Table 1:** ^1^H NMR Spectroscopic Data of Compounds **1**–**6** (*δ*_H_, *J* in Hz) *.

Position	1 *^a^*	2 *^b^*	3 *^b^*	4 *^b^*	5 *^b^*	6 *^b^*
3	7.30, s	7.67, s	7.67, s	7.67, s	7.60, s	7.61, s
5	7.34, s	/	/	/	/	/
8	7.68, s	7.92, s	7.93, s	7.92, s	7.96, s	7.97, s
13	1.74, s	1.44, s	2.17, s	1.97, s	2.10, d (1.0)	2.04, d (1.0)
14	2.29, s	2.35, s	2.35, s	2.33, s	2.36, s	2.37, s
17	6.23, d (10.7)	6.47, d (11.2)	6.47, d (11.0)	6.52, d (11.3)	6.24, d (10.8)	6.28, d (11.1)
18	6.64, t (13.1)	6.80, dd (14.7, 11.2)	6.80, dd (15.0, 11.2)	7.13, dd (15.1, 11.3)	6.49, dd (15.9, 11.0)	6.53, dd (16.6, 11.2)
19	5.93, dd (14.2, 6.7)	6.03, dd (15.1, 7.9)	6.04, dd (15.0, 8.0)	6.07, dd (15.2, 10.2)	6.08, dd (15.9, 6.7)	6.02, dd (15.2, 6.4)
20	2.24, m	2.41, m	2.41, m	2.49, m	2.31, m	2.43, m
21	3.63, d (8.0)	3.76, m	3.78, dd (8.9, 0.7)	3.88, m	4.03, m	4.25, dd (8.6, 2.0)
22	1.74, m	1.88, m	1.90, m	1.65, m	1.86, m	1.94, m
23	3.43, overlap	3.50, dd (3.5, 8.7)	3.50, dd (7.8, 4.6)	3.27, dd (9.9, 3.1)	3.47, dd (10.4, 1.9)	3.46, dd (9.9, 2.3)
24	1.67, m	1.81, m	1.84, m	1.93, m	1.78, m	1.77, m
25	3.82, d (8.8)	3.74, m	4.05, dd (11.3, 2.0)	3.97, dd (8.6, 2.8)	3.97, m	3.95, dd (9.6, 0.7)
26	1.56, m	1.93, m	2.01, m	1.48, m	1.38, m	1.43, m
27	3.71, d (5.84)	3.98, d (3.6)	3.46, t (8.5)	3.17, t (10.2)	4.30, m	3.98, m
28	2.53, overlap	4.39, d (5.2)	3.96, td (8.6, 3.4)	2.51, m	2.86, m	2.61, m
29	6.69, d (9.4)	2.48, m 1.75, m	2.76, dd (15.6, 3.5) 2.65, dd (15.6, 8.9)	5.75, d (8.2, 0.7)	6.26, dd (10.4, 1.1)	6.42, dd (9.2, 2.0)
30	2.04, s	2.07, s	2.07, s	4.46, m 4.24, m	2.07, s	2.08, s
31	0.89, d (6.1)	0.99, d (6.8)	0.99, d (6.2)	1.14, d (6.9)	0.91, d (6.9)	3.53, d (4.7)
32	0.78, d (6.4)	1.02, d (7.0)	0.97, d (5.8)	0.66, d (7.0)	1.05, d (7.0)	1.08, d (3.2)
33	0.82, d (7.2)	0.85, d (7.0)	0.95, d (6.6)	1.06, d (7.0)	0.71, d (6.8)	0.74, d (6.8)
34	0.66, d (6.4)	0.83, d (7.0)	1.04, d (6.5)	0.80, d (6.4)	0.38, d (7.0)	0.37, d (7.0)
34a	0.85, d (6.9)	/	/	5.12, d (4.1)	4.02, m 4.00, m	1.06, d (3.3)
1′	4.82, s				2.03, s	
2′	4.09, s					
3′	3.23, overlapped					
4′	3.33, overlapped					
5′	3.96, m					
6′	1.19, d (5.8)					

* s: singlet, t: triplet, d: doublet, dd: double doublet. ***^a,b^*** Recorded in DMSO-*d*_6_ (dimethyl sulfoxide-*d*_6_, hexadeuterodimethyl sulfoxide) (600 MHz) and CD_3_OD (400 MHz), respectively.

**Table 2:** ^13^C NMR Spectroscopic Data of Compounds **1**–**11** (*δ*_C_).

Position	1 *^a^*	2 *^b^*	3 *^b^*	4 *^b^*	5 *^b^*	6 *^b^*	7 *^b^*	8 *^b^*	9 *^b^*	10 *^b^*	11 *^b^*
1	130.1, C	180.0, C	181.3, C	181.9, C	181.1, C		180.5, C	181.0, C	181.0, C	181.0, C	179.7, C
2	136.9, C	142.0, C	142.3, C	143.1, C	124.7, C		141.2, C	133.7, C	142.0, C	142.6, C	141.8, C
3	104.5, CH	117.4, CH	117.9, CH	116.7, CH	118.9, CH	118.7, CH	118.2, CH	118.5, CH	119.0, CH	119.2, CH	119.0, CH
4	147.7, C		187.2, C		188.0, C			188.0, C	188.0, C		
5	103.7, CH				129.3, C			129.1, C			
6	153.4, C	160.6, C	160.3, C	161.3, C	164.0, C	160.6, C	160.0, C	160.7, C	160.7, C	161.4, C	162.0, C
7	127.4, C	132.5, C	133.1, C	133.5, C	133.6, C	133.1, C		132.2, C	132.3, C	131.5, C	132.9, C
8	123.1, CH	131.2, CH	132.0, CH	132.1, CH	132.5, CH	132.0, CH	131.6, CH	132.0, CH	132.4, CH	132.0, CH	131.2, C
9	137.1, C		124.5, C	136.4, C	126.9, C			124.2, C	129.1, C		
10	122.6, C	130.5, C	130.7, C		132.9, C		132.0, C	142.5, C	132.5, C	133.0, C	133.0, C
11	169.3, C			200.2, C	201.1, C	201.3, C	201.1, C	201.2, C	201.1, C	201.4, C	200.3, C
12	125.9, C	107.5, C	211.0, C	143.3, C	142.6, C	138.5, C	140.7, C	141.7, C	142.2, C	142.2, C	142.9, C
13	12.5, CH_3_	24.4, CH_3_	31.3, CH_3_	17.5, CH_3_	13.2, CH_3_	12.2, CH_3_	12.6, CH_3_	13.0, CH_3_	13.2, CH_3_	13.2, CH_3_	12.5, CH_3_
14	17.1, CH_3_	17.1, CH_3_	17.6, CH_3_	12.8, CH_3_	17.7, CH_3_	17.5, CH_3_	17.1, CH_3_	17.5, CH_3_	17.7, CH_3_	17.5, CH_3_	16.8, CH_3_
15	167.2, C	170.2, C	170.4, C	170.8, C	173.5, C	173.3, C	172.7, C	170.9, C	172.2, C	173.3, C	172.0, C
16	121.0, C	129.9, C	130.1, C	133.0, C	132.5, C	133.5, C	133.4, C	134.3, C	133.4, C	134.3, C	132.9, C
17	133.8, CH	138.5, CH	139.1, CH	141.7, CH	135.6, CH	135.0, CH	134.6, CH	140.0, CH	135.9, CH	133.3, CH	134.5, CH
18	125.9, CH	127.6, CH	128.2, CH	129.4, CH	126.9, CH	128.9, CH	128.4, CH	127.4, CH	126.0, CH	130.4, CH	124.3, CH
19	142.8, CH	145.8, CH	146.6, CH	146.3, CH	142.0, CH	137.8, CH	137.5, CH	146.2, CH	148.2, CH	136.6, CH	142.8, CH
20	40.6, CH	42.4, CH	43.0, CH	48.2, CH	39.8, CH	48.1, CH	47.4, CH	39.3, CH	76.9, CH	52.7, CH	82.3, C
21	73.2, CH	75.7, CH	76.3, CH	78.0, CH	75.5, CH	72.2, CH	71.7, CH	76.6, CH	76.6, CH	73.9, CH	86.4, CH
22	36.2, CH	35.8, CH	37.3, CH	43.8, CH	35.0, CH	35.5, CH	35.2, CH	34.6, CH	35.2, CH	50.0, CH	48.3, CH
23	76.7, CH	78.1, CH	79.6, CH	82.1, CH	79.5, CH	79.4, CH	79.1, CH	79.5, CH	80.7, CH	211.3, C	106.8, C
24	35.0, CH	37.6, CH	39.1, CH	34.5, CH	38.7, CH	38.7, CH	38.5, CH	38.4, CH	38.9, CH	50.8, CH	43.0, CH
25	69.9, CH	75.4, CH	84.2, CH	73.7, CH	71.9, CH	71.6, CH	71.2, CH	71.7, CH	72.2, CH	71.5, CH	73.8, CH
26	38.2, CH	34.5, CH	44.8, CH	41.8, CH	44.5, CH	44.1, CH	44.0, CH	44.5, CH	44.5, CH	42.8, CH	43.7, CH
27	72.3, CH	89.2, CH	83.4, CH	74.2, CH	69.2, CH	74.5, CH	68.8, CH	69.6, CH	69.7, CH	68.9, CH	68.3, CH
28	37.1, CH	71.2, CH	81.5, CH	49.8, CH	46.8, CH	41.4, CH	49.7, CH	50.3, CH	50.3, CH	49.7, CH	48.9, CH
29	147.0, CH	46.6, CH_2_	49.1, CH_2_	145.8, CH	140.5, CH	147.0, CH	141.9, CH	142.0, CH	142.5, CH	141.3, CH	142.0, CH
30	20.6, CH_3_	20.6, CH_3_	21.1, CH_3_	65.7, CH_2_	20.9, CH_3_	20.6, CH_3_	20.1, CH_3_	65.4, CH_2_	20.9, CH_3_	20.9, CH_3_	20.3, CH_3_
31	16.8, CH_3_	17.2, CH_3_	17.9, CH_3_	20.3, CH_3_	18.7, CH_3_	63.9, CH_2_	63.5, CH_2_	18.2, CH_3_	26.7, CH_3_	65.1, CH_2_	28.7, CH_3_
32	10.1, CH_3_	11.3, CH_3_	11.6, CH_3_	12.2, CH_3_	11.8, CH_3_	12.5, CH_3_	12.0, CH_3_	11.3, CH_3_	14.5, CH_3_	15.5, CH_3_	13.0, CH_3_
33	10.4, CH_3_	10.3, CH_3_	11.3, CH_3_	12.1, CH_3_	9.5, CH_3_	9.3, CH_3_	8.8, CH_3_	9.4, CH_3_	9.7, CH_3_	8.5, CH_3_	8.6, CH_3_
34	9.0, CH_3_	12.6, CH_3_	14.9, CH_3_	12.9, CH_3_	12.3, CH_3_	11.5, CH_3_	11.7, CH_3_	11.9, CH_3_	12.3, CH_3_	12.2, CH_3_	12.4, CH_3_
34a	16.9, CH_3_			94.8, CH	66.4, CH_2_	20.0, CH_3_	64.3, CH_2_	65.0, CH_2_	65.0, CH_2_	65.0, CH_2_	64.8, CH_2_
1′	105.1, CH				173.0, C						
2′	70.8, CH				21.5, CH_3_						
3′	73.0, CH										
4′	72.1, CH										
5′	71.1, CH										
6′	18.0, CH_3_										

***^a,b^*** Recorded in DMSO-*d*_6_ (150 MHz) and CD_3_OD (100 MHz), respectively; CH_3_: primary carbon; CH_2_: secondary carbon; CH: tertiary carbon; C: quaternary carbon.

**Table 3:** ^1^H NMR (400 MHz) Spectroscopic Data of Compounds **7**–**11** (*δ*_H_, in MeOD, *J* in Hz) *.

Position	7	8	9	10	11
3	7.61, s	7.59, s	7.58, s	7.59, s	7.43, s
8	7.97, s	7.93, s	7.94, s	7.92, s	7.89, s
13	2.09, s	2.10, s	2.08, s	2.07, s	2.04, s
14	2.36, s	2.38, s	2.35, s	2.34, s	2.31, s
17	6.29, dd (11.0, 1.0)	6.51, br d (10.9)	6.24, dd (10.8, 0.8)	6.26, dd (10.9, 1.1)	6.22, dd (11.4, 1.4)
18	6.53, dd (15.9, 11.0)	6.90, dd (16.1, 11.0)	6.45, dd (15.9, 10.9)	6.13, dd (15.1, 11.0)	6.29, br d (14.4)
19	6.01, dd (15.8, 7.0)	6.36, dq (7.4, 1.3)	5.95, br d (16.0)	5.82, dd (15.1, 12.4)	5.84, d (14.4)
20	2.42, m	2.32, m		1.85, m	
21	4.26, dd (9.1, 1.8)	4.05, br d (9.9)	3.94, br s	3.81, br d (10.0)	3.80, d (10.5)
22	1.95, m	1.93, m	2.00, m	2.93, m	1.79, m
23	3.47, m	3.49, dd (10.3, 1.8)	3.42, dd (9.4, 2.6)		
24	1.78, m	1.81, m	1.71, m	2.45, dd (7.4, 0.72)	1.91, m
25	3.97, dd (10.2, 1.0)	3.97, dd (10.2, 1.2)	3.92, dd (10.2, 1.0)	3.86, br d (9.6)	4.23, dd (10.2, 0.7)
26	1.40, m	1.42, m	1.39, m	1.32, m	1.46, m
27	4.37, m	4.38, d (6.6)	4.35, br s	4.41, m	4.28, br s
28	2.66, m	2.69, q (7.7)	2.65, qd (7.9, 1.2)	2.56, m	2.56, m
29	6.32, dd (9.4, 1.1)	6.34, d (6.4)	6.29, dd (9.5, 0.7)	6.22, dd (9.2, 1.4)	6.28, d (3.5)
30	2.08, s	4.36, br d (12.1) 4.23, br d (12.1)	2.09, s	2.04, s	2.08, s
31	3.54, m	0.94, d (7.0)	1.02, s	4.37, d (12.8) 4.23, d (12.8)	1.31, s
32	1.08, d (7.0)	1.06, d (7.0)	1.17, d (7.0)	1.03, d (6.8)	1.13, d (6.5)
33	0.73, d (6.8)	0.74, d (6.8)	0.74, d (6.8)	1.13, d (7.4)	0.97, d (7.2)
34	0.39, d (7.0)	0.42, d (7.0)	0.40, d (7.0)	0.44, d (7.0)	0.55, d (8.1)
34a	3.56, m	3.61, dd (10.9, 7.9)	3.59, dd (10.9, 7.8)	3.50, m 3.39, m	3.46, m 3.35, m
		3.43, dd (10.9, 7.9)	3.40, dd (10.9, 7.8)		

* s: singlet, t: triplet, d: doublet, dd: double doublet.

## Supplementary Materials

The following are available online at https://www.mdpi.com/2218-273X/10/9/1265/s1, Figure S1: Construction and enzymatic digestion verification of *rifT* gene knock-out plasmid pOJ260-*rifT*; Figure S2: PCR verification of *rifT* gene knock-out single crossover mutant; Figure S3: PCR verification of *rifT* gene knock-out double crossover mutant; Figure S4: Construction flow chart of *rifT* gene knock-out mutant; Figure S5: Construction and enzymatic digestion verification of *rifT* gene complementation plasmid pSET152-*rifK*p-*rifT*; Figure S6: PCR verification of *rifT* gene complementation mutant *ΔrifT::rifT*; Figure S7: HPLC detection of *rifT* gene deletion and complementation mutant; Figure S8: Structures of known compounds **12**–**18**; Figures S9–S14: NMR and HRESIMS spectra of **1**; Figures S15–S17: Analysis of the sugar moiety of **1**; Figures S18–S77**:** NMR and HRESIMS spectra of **2**–**11**; Figure S78: Antimicrobial activity against *Staphylococcus aureus* ATCC 25923 and *Proteusbacillus vulgaris* CPCC 160013; Figure S79: SDS-PAGE analysis of the inhibitory activity of compounds **1–18** (100μM, respectively) against the T3SS of *S. enterica* Typhimurium UK-1 χ8956; Tables S1–S11: NMR spectroscopic data for **1**–**11** in DMSO-*d*_6_ or CD_3_OD (*δ* in ppm, *J* in Hz); Table S12: Diameter of the inhibition zones and MIC of active compounds against *Staphylococcus aureus* ATCC 25923; Table S13: The OD_600_ value of *Staphylococcus aureus* ATCC 25923 bacterial solution in different concentration gradients of active compounds; Table S14: Antiproliferative activity against KG1 cells of compounds **14**–**18**.

## References

[1] Rinehart K.L., Shield L.S. (1976). Chemistry of the ansamycin antibiotics. Fortschritte der Chemie Organischer Naturstoffe.

[2] Wehrli W. (1977). Ansamycins chemistry, biosynthesis and biological activity. Topics in Current Chemistry.

[3] Maggi N., Pasqualucci C.R., Ballotta R., Sensi P. (1966). Rifampicin: A new orally active rifamycin. Chemotherapy.

[4] Cassady J.M., Chan K.K., Floss H.G., Leistner E. (2004). Recent developments in the maytansinoid antitumor agents. Chem. Pharm. Bull..

[5] Kusari P., Kusari S., Eckelmann D., Zühlke S., Kayser O., Spiteller M. (2016). Cross-species biosynthesis of maytansine in *Maytenus serrata*. RSC Adv..

[6] Whitesell L., Mimnaugh E.G., De Costa B., Myers C.E., Neckers L.M. (1994). Inhibition of heat shock protein HSP90-pp60v-src heteroprotein complex formation by benzoquinone ansamycins: Essential role for stress proteins in oncogenic transformation. Proc. Natl. Acad. Sci. USA.

[7] Kang Q., Shen Y., Bai L. (2012). Biosynthesis of 3,5-AHBA-derived natural products. Nat. Prod. Rep..

[8] Sensi P., Margalith P., Timbal M.T. (1959). Rifamycin, a new antibiotic. Farmaco. Sci..

[9] Sensi P., Greco A.M., Gallo G.G., Rolland G. (1957). Isolation and structure determination of a new amicetin-like antibiotic: Amicetin B. Antibiot. Chemother..

[10] Sensi P. (1957). Applications of paper chromatography & countercurrent distribution to steroids & antibiotics. Boll. Chim. Farm..

[11] Wehrli W., Staehelin M. (1971). Actions of the rifamycins. Bacteriol. Rev..

[12] Steffen R., Jiang Z.D., Gracias Garcia M.L., Araujo P., Stiess M., Nacak T., Greinwald R., DuPont H.L. (2018). Rifamycin SV-MMX(R) for treatment of travellers’ diarrhea: Equally effective as ciprofloxacin and not associated with the acquisition of multi-drug resistant bacteria. J. Travel Med..

[13] Cho N.K., Sunada Y., Nohara S. (1963). Clinical studies on a new antibiotic, rifamycin SV. J. Showa Med Assoc..

[14] Girling D.J. (1977). Adverse reactions to rifampicin in antituberculosis regimens. J. Antimicrob. Chemother..

[15] Goldstein B.P. (2014). Resistance to rifampicin: A review. J. Antibiot..

[16] Yu T.W., Shen Y.M., Doi-Katayama Y., Tang L., Park C., Moore B.S., Hutchinson C.R., Floss H.G. (1999). Direct evidence that the rifamycin polyketide synthase assembles polyketide chains processively. Proc. Natl. Acad. Sci. USA.

[17] Xu J., Wan E., Kim C.J., Floss H.G., Mahmud T. (2005). Identification of tailoring genes involved in the modification of the polyketide backbone of rifamycin B by *Amycolatopsis mediterranei* S699. Microbiology.

[18] August P.R., Li T., Yoon Y.J., Ning S., Muller R., Yu T.W., Taylor M., Hoffmann D., Kim C.G., Zhang X. (1998). Biosynthesis of the ansamycin antibiotic rifamycin: Deductions from the molecular analysis of the *rif* biosynthetic gene cluster of *Amycolatopsis mediterranei* S699. Chem. Biol..

[19] Floss H.G., Yu T.W. (2005). Rifamycins-mode of action, resistance, and biosynthesis. Chem. Rev..

[20] Tang L., Yoon Y.J., Choi C.Y., Hutchinson C.R. (1998). Characterization of the enzymatic domains in the modular polyketide synthase involved in rifamycin B biosynthesis by *Amycolatopsis mediterranei*. Gene.

[21] Floss H.G., Yu T.W. (1999). Lessons from the rifamycin biosynthetic gene cluster. Curr. Opin. Chem. Biol..

[22] Stratmann A., Toupet C., Schilling W., Traber R., Oberer L., Schupp T. (1999). Intermediates of rifamycin polyketide synthase produced by an *Amycolatopsis mediterranei* mutant with inactivated *rifF* gene. Microbiology.

[23] Bierman M., Logan R., O’Brien K., Seno E.T., Rao R.N., Schoner B.E. (1992). Plasmid cloning vectors for the conjugal transfer of DNA from *Escherichia coli* to *Streptomyces* spp.. Gene.

[24] Gibson D.G., Young L., Chuang R.Y., Venter J.C., Hutchison C.A., Smith H.O. (2009). Enzymatic assembly of DNA molecules up to several hundred kilobases. Nat. Methods.

[25] Ding X.M., Tian Y.Q., Chiao J.S., Zhao G.P., Jiang W.H. (2003). Stability of plasmid pA387 derivatives in *Amylcolatopsis mediterranei* producing rifamycin. Biotechnol. Lett..

[26] Raahave D. (1974). Paper disk-ager diffusion assay of penicillin in the presence of streptomycin. Antimicrob. Agents Chemother..

[27] Arendrup M.C., Prakash A., Meletiadis J., Sharma C., Chowdhary A. (2017). Comparison of EUCAST and CLSI reference microdilution MICs of eight antifungal compounds for *Candida auris* and associated tentative epidemiological cutoff values. Antimicrob. Agents Chemother..

[28] Jiang Z., Zhou Q., Ge C., Yang J., Li H., Chen T., Xie H., Cui Y., Shao M., Li J. (2019). Rpn10 promotes tumor progression by regulating hypoxia-inducible factor 1 alpha through the PTEN/Akt signaling pathway in hepatocellular carcinoma. Cancer Lett..

[29] Crump J.A., Luby S.P., Mintz E.D. (2004). The global burden of typhoid fever. Bull. World Health Organ..

[30] Linington R.G., Robertson M., Gauthier A., Finlay B.B., van Soest R., Andersen R.J. (2002). Caminoside A, an antimicrobial glycolipid isolated from the marine sponge *Caminus sphaeroconia*. Organic Lett..

[31] Sun Y.H., Rolan H.G., Tsolis R.M. (2007). Injection of flagellin into the host cell cytosol by *Salmonella enterica* serotype Typhimurium. J. Biol. Chem..

[32] Lostroh C.P., Lee C.A. (2001). The *Salmonella* pathogenicity island-1 type III secretion system. Microbes Infect..

[33] Worrall L.J., Lameignere E., Strynadka N.C. (2011). Structural overview of the bacterial injectisome. Curr. Opin. Microbiol..

[34] Li J., Sun W., Guo Z., Lu C., Shen Y. (2014). Fusaric acid modulates type three secretion system of *Salmonella enterica* serovar Typhimurium. Biochem. Biophys. Res. Commun..

[35] Zhang Z., Zhang J., Song R., Guo Z., Wang H., Zhu J., Lu C., Shen Y. (2017). Ansavaricins A–E: Five new streptovaricin derivatives from *Streptomyces* sp. S012. RSC Adv..

[36] Guo Z., Li X., Li J., Yang X., Zhou Y., Lu C., Shen Y. (2016). Licoflavonol is an inhibitor of the type three secretion system of *Salmonella enterica* serovar Typhimurium. Biochem. Biophys. Res. Commun..

[37] Curtiss R., Wanda S.Y., Gunn B.M., Zhang X., Tinge S.A., Ananthnarayan V., Mo H., Wang S., Kong W. (2009). *Salmonella enterica* serovar Typhimurium strains with regulated delayed attenuation in vivo. Infect. Immun..

[38] Li J., Lv C., Sun W., Li Z., Han X., Li Y., Shen Y. (2013). Cytosporone B, an inhibitor of the type III secretion system of *Salmonella enterica* serovar Typhimurium. Antimicrob. Agents Chemother..

[39] Wehrli W., Staeheli M. (1969). The rifamycins-relation of chemical structure and action on RNA polymerase. Biochim. Biophys. Acta.

[40] Stratmann A., Schupp T., Toupet C., Schilling W., Oberer L., Traber R. (2002). New insights into rifamycin B biosynthesis: Isolation of proansamycin B and 34a-deoxy-rifamycin was early macrocyclic intermediates indicating two separated biosynthetic pathways. J. Antibiot..

[41] Shi Y., Zhang J., Tian X., Wu X., Li T., Lu C., Shen Y. (2019). Isolation of 11,12- *seco*-rifamycin W derivatives reveals a cleavage pattern of the rifamycin *ansa* chain. Organic Lett..

[42] Ghisalba O., Traxler P., Nuesch J. (1978). Early intermediates in the biosynthesis of ansamycins. I. Isolation and identification of protorifamycin I. J. Antibiot..

[43] Ghisalba O., Traxler P., Fuhrer H., Richter W.J. (1980). Early intermediates in the biosynthesis of ansamycins. III. Isolation and identification of further 8-deoxyansamycins of the rifamycin-type. J. Antibiot. (Tokyo).

[44] White R.J., Martinelli E., Lancini G. (1974). Ansamycin biogenesis: Studies on a novel rifamycin isolated from a mutant strain of *Nocardia mediterranei*. Proc. Natl. Acad. Sci. USA.

[45] Guo J., Frost J.W. (2002). Kanosamine Biosynthesis:  A Likely Source of the Aminoshikimate Pathway’s Nitrogen Atom. J. Am. Chem. Soc..

[46] Arakawa K., Müller R., Mahmud T., Yu T.W., Floss H.G. (2002). Characterization of the Early Stage Aminoshikimate Pathway in the Formation of 3-Amino-5-hydroxybenzoic Acid:  The RifN Protein Specifically Converts Kanosamine into Kanosamine 6-Phosphate. J. Am. Chem. Soc..

[47] Zhao G., Li S., Guo Z., Sun M., Lu C. (2015). Overexpression of div8 increases the production and diversity of divergolides in Streptomyces sp. W112. RSC Adv..

[48] Li S., Lu C., Ou J., Deng J., Shen Y. (2015). Overexpression of hgc1 increases the production and diversity of hygrocins in Streptomyces sp. LZ35. RSC Adv..

[49] Wang J., Li W., Wang H., Lu C. (2018). Pentaketide Ansamycin Microansamycins A-I from Micromonospora sp. Reveal Diverse Post-PKS Modifications. Organic Lett..

