# Supplementary material for: 8-Deoxy-Rifamycin Derivatives from Amycolatopsis mediterranei S699 ΔrifT Strain
Source: Biomolecules. 2020 Sep 2;10(9):1265. doi: 10.3390/biom10091265 (PMC7563148; doi:10.3390/biom10091265)
Supplement: Supplementary file 1 [file biomolecules-10-01265-s001.pdf]

Supplementary Material

8-Deoxy-Rifamycin Derivatives from *Amycolatopsis mediterranei* S699 *ΔrifT* Strain

Feng Ye [1,†], Yanrong Shi [1,†], Shengliang Zhao [1], Zhiying Li [1], Haoxin Wang [2], Chunhua Lu [1] and Yuemao Shen [1,2,*]

[1] Key Laboratory of Chemical Biology (Ministry of Education), School of Pharmaceutical Sciences, Cheeloo College of Medicine, Shandong University, No. 44 West Wenhua Road, Jinan, Shandong 250012, China; yefeng1997@mail.sdu.edu.cn (F.Y.); yrshi910212@sdu.edu.cn (Y.S.); 201720261108@mail.sdu.edu.cn (S.Z.); 201936104@mail.sdu.edu.cn (Z.L.); ahua0966@sdu.edu.cn (C.L.)

[2] State Key Laboratory of Microbial Technology, Shandong University, Qingdao, Shandong 266237, China; wanghaoxin@sdu.edu.cn

* Correspondence: yshen@sdu.edu.cn; Tel.: +86-531-8838-2108

† Contributed equally to this work.

Contents

1. Construction of the mutant strain *Amycolatopsis mediterranei* S699 Δ*rifT*.. 4

1.1 Construction of the plasmid pOJ260-rifT.. 5

1.2 Construction of the mutant strain Amycolatopsis mediterranei S699 ΔrifT.. 5

1.3 Construction of the rifT gene complementation mutant.. 6

1.3.1 Construction of the rifT gene complementation plasmid.. 6

1.3.2 Construction of the rifT gene complementation mutant.. 6

1.4 HPLC analysis of the metabolites of Amycolatopsis mediterranei S699 ΔrifT and the rifT gene complementation mutant strain ΔrifT::rifT.. 7

Figure S8. Structures of known compounds **12–18**.. 8

2. NMR data of compounds 1–11.. 8

Table S1. NMR spectroscopic data for **1** in DMSO-d_6 (*δ* in ppm, *J* in Hz).. 8

Table S2. NMR spectroscopic data for **2** in CD_3OD (*δ* in ppm, *J* in Hz).. 9

Table S3. NMR spectroscopic data for **3** in CD_3OD (*δ* in ppm, *J* in Hz).. 10

Table S4. NMR spectroscopic data for **4** in CD_3OD (*δ* in ppm, *J* in Hz).. 11

Table S5. NMR spectroscopic data for **5** in CD_3OD (*δ* in ppm, *J* in Hz).. 12

Table S6. NMR spectroscopic data for **6** in CD_3OD (*δ* in ppm, *J* in Hz).. 13

Table S7. NMR spectroscopic data for **7** in CD_3OD (*δ* in ppm, *J* in Hz).. 14

Table S8. NMR spectroscopic data for **8** in CD_3OD (*δ* in ppm, *J* in Hz).. 15

Table S9. NMR spectroscopic data for **9** in CD_3OD (*δ* in ppm, *J* in Hz).. 16

Table S10. NMR spectroscopic data for **10** in CD_3OD (*δ* in ppm, *J* in Hz).. 17

Table S11. NMR spectroscopic data for **11** in CD_3OD (*δ* in ppm, *J* in Hz).. 18

3. NMR and HRESIMS spectra of compounds 1–11.. 19

Figures S9–14. HRESIMS and NMR spectra of **1**.. 19

Figures S15-17. Analysis of sugar stereochemistry in compound **1**.. 22

Figures S18–23. HRESIMS and NMR spectra of **2**.. 24

Figures S24–29. HRESIMS and NMR spectra of **3**.. 27

Figures S30–35. HRESIMS and NMR spectra of **4**.. 30

Figures S36–50. HRESIMS and NMR spectra of **5**.. 33

Figures S42–47. HRESIMS and NMR spectra of **6**.. 36

Figures S48–50. HRESIMS spectra of **7**–**9**.. 39

Figures S51–55. NMR spectra of **7**.. 40

Figures S56–60. NMR spectra of **8**.. 43

Figures S61–65. NMR spectra of 9.. 45

Figures S66 and S67. HRESIMS spectra of **10** and **11**.. 48

Figures S68–72. NMR spectra of **10**.. 49

Figures S73–77. NMR spectra of **11**.. 51

4. Bioactivity.. 54

*4.1 Antimicrobial activity of compounds **1–18** (Figure S78)*.. 54

Table S12. Diameter of the inhibition zones and MIC of active compounds against Staphylococcus aureus ATCC 25923 54

Table S13. The OD_{600} value of Staphylococcus aureus ATCC 25923 bacterial solution in different concentration gradients of active compounds 54

*Table S14. Antiproliferative activity against KG1 cells of compounds **14**-**18***........ 55

*4.2 Anti-Type III secretion system (T3SS) activity of compounds **1**-**18** (Figure S79)*........ 56

1. Construction of the mutant strain *Amycolatopsis mediterranei* S699 *ΔrifT*

A

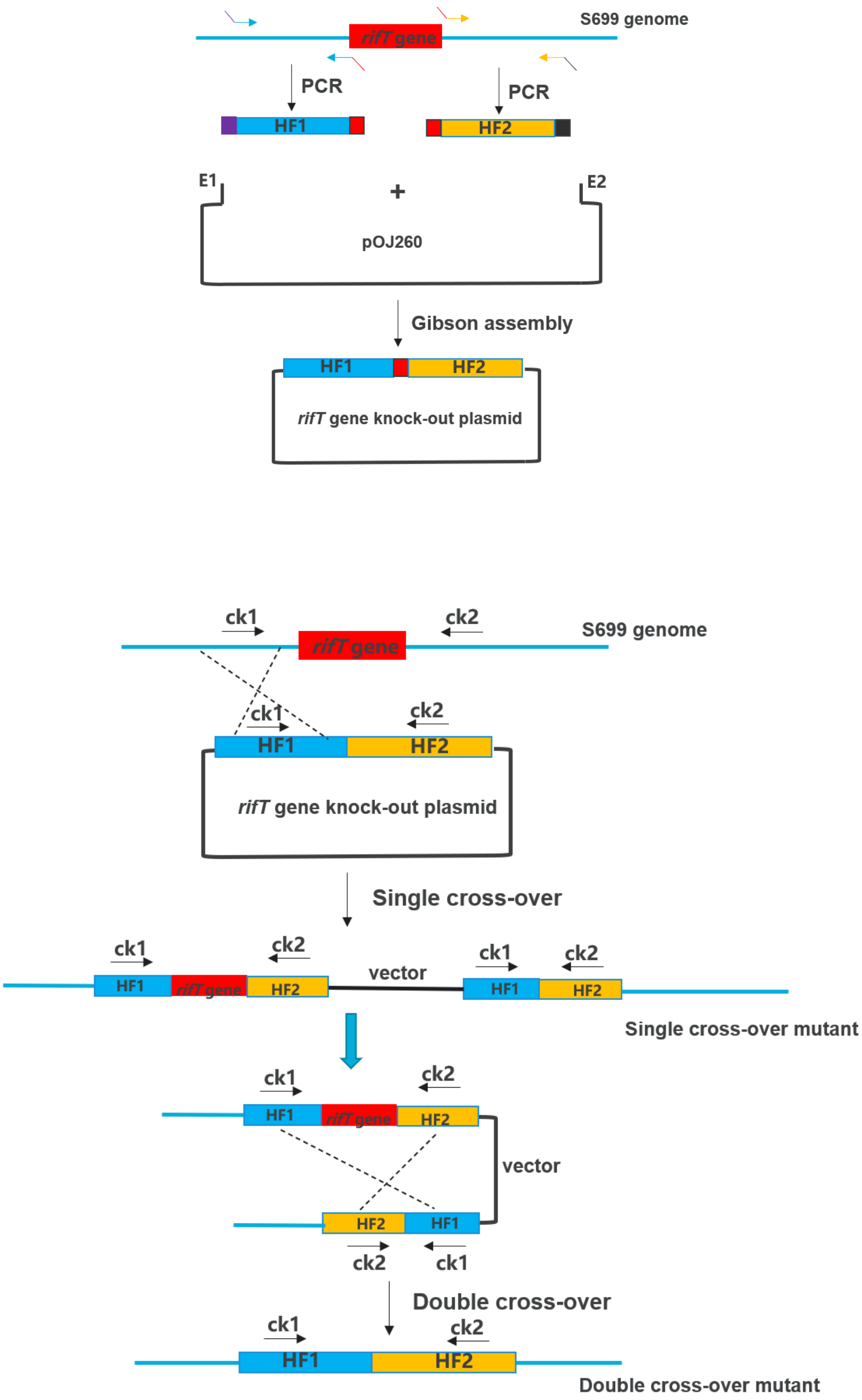

Figure S1. Flow chart for the construction of *Amycolatopsis mediterranei* S699 *ΔrifT*. (**A**) Construction of the plasmid pOJ260-*rifT*; (**B**) Construction of *Amycolatopsis mediterranei* S699 *ΔrifT*.

1.1 Construction of the plasmid pOJ260-rifT

The left and right homologous fragments rif-HF_1 and rif-HF_2 were amplified by PCR with the primer pairs of *rifT*-HF_1-F/R and *rifT*-HF_2-F/R using the genomic DNA of *Amycolatopsis mediterranei* S699 as a template, and ligated to the vector pOJ260 by Gibson Assembly. The resulting construct pOJ260-rifT was verified by KpnI digestion, and further conformed by DNA sequencing (Figure S2).

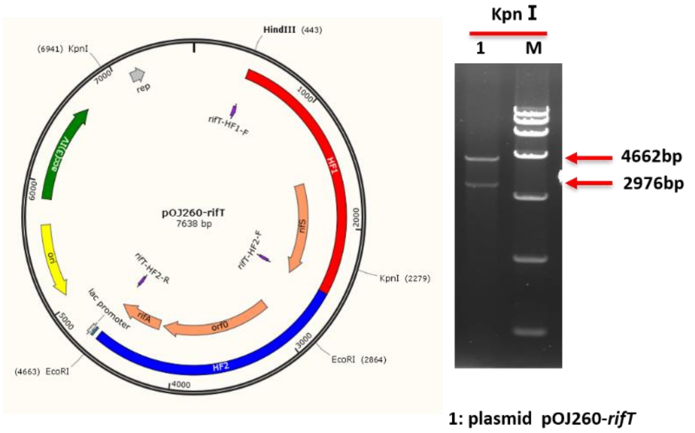

Figure S2. Verification of the *rifT* gene knock-out plasmid pOJ260-*rifT*.

1.2 Construction of the mutant strain Amycolatopsis mediterranei S699 ΔrifT

1) Plasmid pOJ260-*rifT* was transformed into *Amycolatopsis mediterranei* S699 wild type strain, and the transformants were selected on YMG agar plate (60 μg/mL Apr). The single crossover mutants were verified by PCR with the primer pairs of *rifT*-CK-F/R (Figure S3).

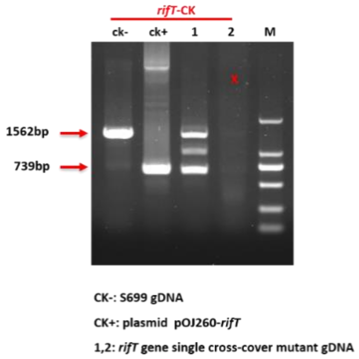

Figure S3. PCR verification of the single cross-over mutants.

2) The verified single crossover mutant strain was cultivated on YMG agar plates without antibiotics for four generation to allow the production of the double crossover mutants. The candidates were first selected by comparing their growth on YMG agar plates with or without Apr (60 μg/mL). The ones which cannot grow on YMG agar plates with apramycin were further screened by PCR with the primer pairs of *rifT*-CK-F/R to identify the *rifT* gene deleted mutant strain (Figure S4).

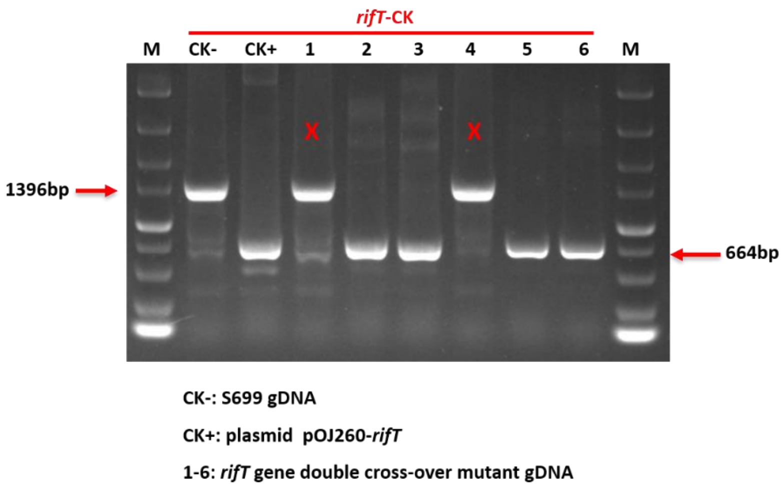

Figure S4. PCR verification of *rifT* gene double cross-over mutant.

1.3 Construction of the rifT gene complementation mutant

1.3.1 Construction of the rifT gene complementation plasmid

The *rifT* gene was amplified by PCR with the primer pairs of *rifT*-CP-F/R and ligated into the integrative vector pSET152 to produce pSET152-pRIFK-*rifT*, which were verified by PCR and further confirmed by DNA sequencing (Figure S5).

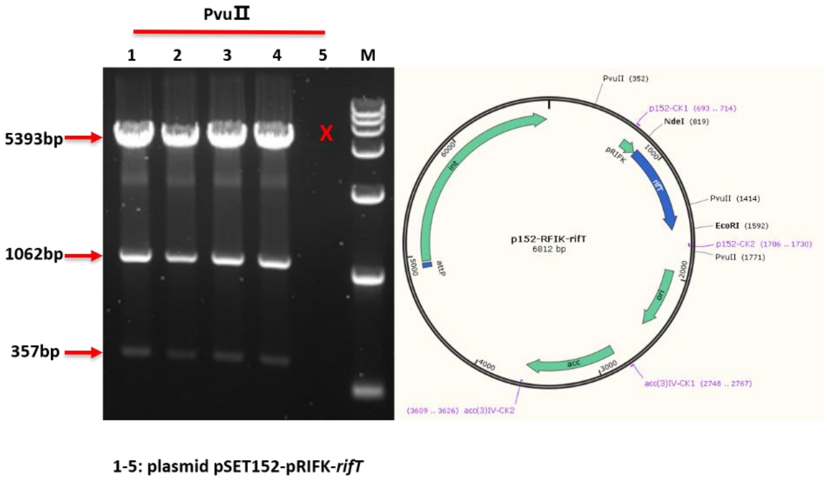

Figure S5. Verification of the *rifT* gene complementation plasmid pSET152-pRIFK-*rifT*.

1.3.2 Construction of the rifT gene complementation mutant

1) The construct pSET152-pRIFK-*rifT* was electroporated into *Amycolatopsis mediterranei S699 ΔrifT*. The transformants were selected on YMG agar plates (60 µg / mL Apr) and verified by PCR with the primer pairs of *rifT*-CK-F/R, *rifT*-CP-F/R and pSET152-CK-F/R to obtain the *rifT* gene complementation mutant *ΔrifT:: rifT* (Figure S6).

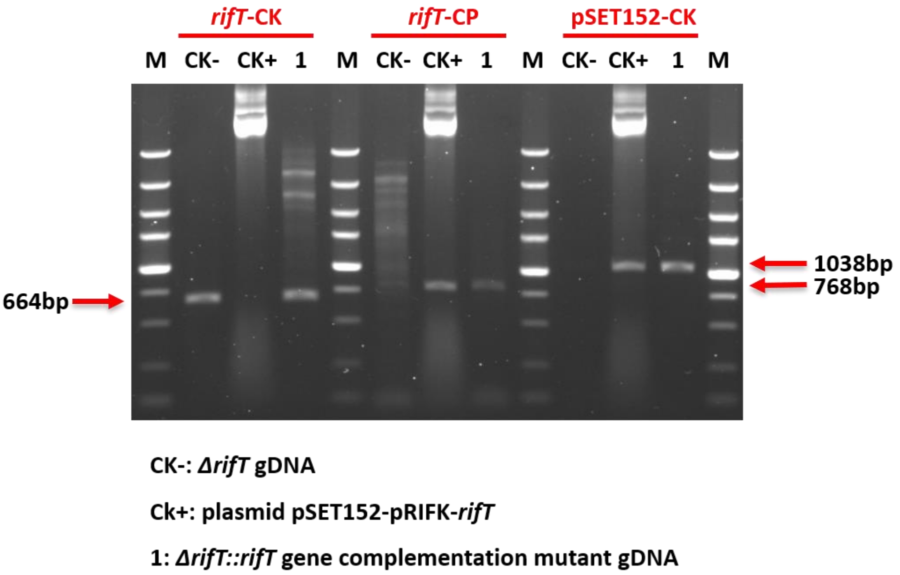

Figure S6. PCR verification of the *rifT* gene complementation mutant *ΔrifT::rifT.*

Primers used in this study

primers	Nucleotide sequence (5′ to 3′)
rifT-HF$_1$-F	ccagtgcc**AAGCTT**ctcggaagtcagcgattcggccgt
rifT-HF$_1$-R	gctgctcccctcaccggcagttcga
rifT-HF$_2$-F	gtgaggggagcagccgcggcttcatctctaagggtct
rifT-HF$_2$-R	ctatgacatgattac**GAATTC**tcgtccgcggcgggctcg
rifT-CK-F	gaaggggccgtcgcgacc
rifT-CK-R	gatgtcgcagccgtcgacg
rifT-CP-F	Ggagattcggaga**CATATG**gtgaaggtcgccatcct
rifT-CP-R	acatgattac**GAATTC**ttacaggtcgacgggctgc
pSET152-CK- F	ctaccaagccgagggatgtaag
pSET152-CK- R	gcaacgcaattaatgtgagttagct

1.4 HPLC analysis of the metabolites of Amycolatopsis mediterranei S699 ΔrifT and the rifT gene complementation mutant strain ΔrifT::rifT

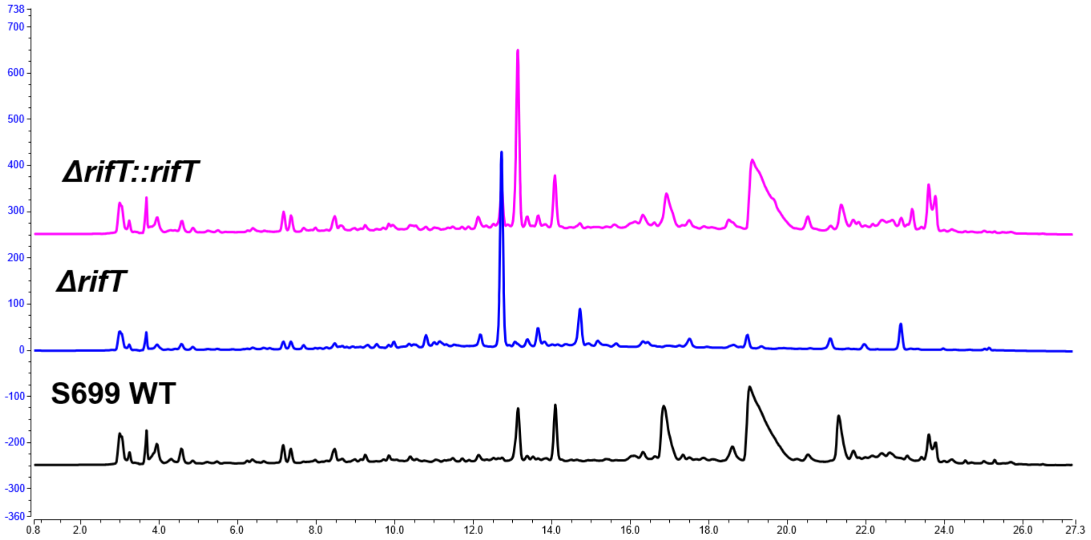

Figure S7. HPLC analysis of the *rifT* gene knock-out and complementation mutants.

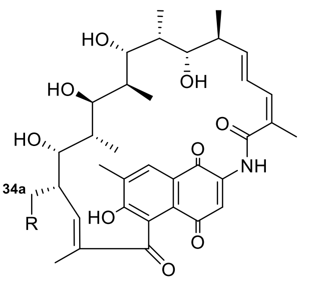

12 (proansamycin B) R = H
13 (protorifamycin I) R = OH

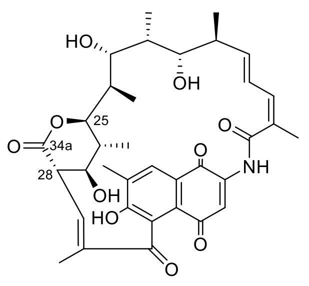

14 (protorifamycin I-lactone)

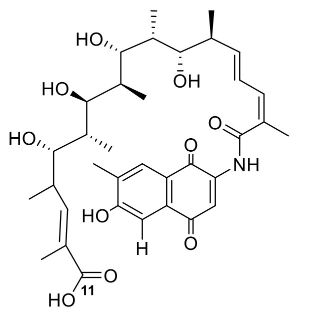

15 (proansamycin B-M$_1$)

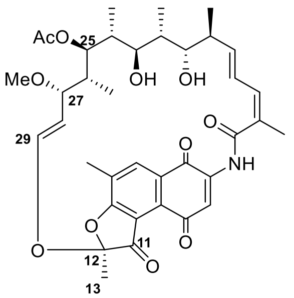

16 (8-deoxy-rifamycin S)

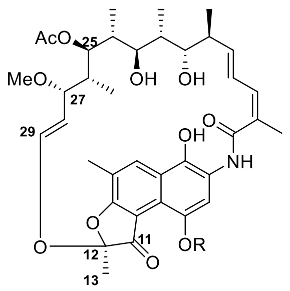

17 (8-deoxy-rifamycin SV) R = H
18 (8-deoxy-rifamycin B) R = CH$_2$COOH

Figure S8. Structures of known compounds **12–18**

2. NMR data of compounds 1–11

Table S1. NMR spectroscopic data for **1** in DMSO-*d*$_6$ (*δ* in ppm, *J* in Hz)

No.	δ_H (mult.)	δ_C mult.	HMBC	^{1}H-^{1}H COSY
1		130.1s		
2		136.9s		
3	7.30 (s)	104.5d	C-9, C-10	
4		147.7s		
5	7.34 (s)	103.7d	C-4, C-6, C-7, C-8	
6		153.4s		
7		127.4s		
8	7.68 (s)	123.1d	C-6, C-8, C-9, C-13	H-13
9		137.1s		
10		122.6s		
11		169.3s		
12		125.9s		
13	1.74 (s)	12.5q	C-11, C-12, C-29	H-29
14	2.29 (s)	17.1q	C-6, C-7, C-8	
15		167.2s		
16		121.0s		
17	6.23 (d, 10.7)	133.8d		H-18, H-30
18	6.64 (t, 13.1)	125.9d	C-15	H-17, H-19
19	5.93 (dd, 14.2, 6.7)	142.8d	C-16	H-18, H-20
20	2.24 (m)	40.6d		H-21, H-31
21	3.63 (dd, 8.0)	73.2d	C-32	H-20
22	1.74 (m)	36.2d		H-23, H-32
23	3.43 (m)	76.7d		H-22
24	1.67 (m)	35.0d		H-33
25	3.82 (d, 8.8)	69.9d	C-27, C-33	H-23, H-26
26	1.56 (m)	38.2d		H-25, H-34
27	3.71 (dd, 5.84)	72.3d		H-28
28	2.53 (m)	37.1d	C-27	H-29, H-34a

29	6.69 (d, 9.4)	147.0d	C-11, C-13	H-13, H-28
30	2.04 (s)	20.6q	C-15, C-16, C-17	H-17
31	0.89 (d, 6.1)	16.8q	C-19, C-20, C-21	H-20
32	0.78 (d, 6.4)	10.1q	C-21, C-22, C-23	H-22
33	0.82 (d, 7.2)	10.4q	C-23, C-24, C-25	H-24
34	0.66 (d, 6.4)	9.0q	C-25, C-26, C-27	H-26
34a	0.85 (d, 6.9)	16.9q	C-27, C-28, C-29	H-28
1′	4.82 (s)	105.1d	C-2′, C-2	H-2′
2′	4.09 (s)	70.8d		
3′	3.23	73.0d		
4′	3.33	72.1d	C-2′, C-6′	H-5′
5′	3.96 (m)	71.1d		H-4′, H-6′
6′	1.19 (d, 5.8)	18.0q	C-1′, C-2′	H-5′

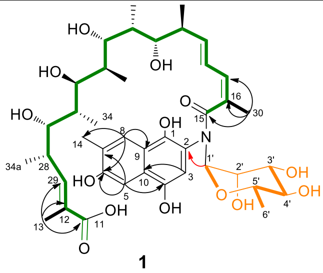

1

Table S2. NMR spectroscopic data for **2** in CD_3OD (δ in ppm, *J* in Hz)

No.	δ_H (mult.)	δ_C mult.	HMBC	^1H-^1H COSY
1		180.0s		
2		142.0s		
3	7.67 (s)	117.4d	C-1, C-2, C-10	H-14
4		nd[a]		
5		nd[a]		
6		160.6s		
7		132.5s		
8	7.92 (s)	131.2d	C-1, C-6, C-10, C-14	
9		nd[a]		
10		130.5s		
11		nd[a]		
12		107.5s		
13	1.44 (s)	24.4q	C-12, C-29	
14	2.35 (s)	17.1q	C-6, C-7, C-8	
15		170.2s		
16		129.9s		
17	6.47 (d, 11.2)	138.5d	C-15, C-30	H-18, H-30
18	6.80 (dd, 11.2, 14.7)	127.6d		H-17, H-19
19	6.03 (dd, 7.9, 15.1)	145.8d	C-17	H-18, H-20
20	2.41 (m)	42.4d	C-18, C-21	H-19, H-21, H-31
21	3.76 (m)	75.7d		H-20, H-22
22	1.88 (m)	35.8d		H-23, H-32
23	3.50 (dd, 3.5, 8.7)	78.1d	C-24, C-25	H-24
24	1.81 (m)	37.6d	C-25, C-33	H-23, H-33
25	3.74 (m)	75.4d	C-24, C-33, C-34	H-24, H-26
26	1.93 (m)	34.5d		H-25, H-27, H-34
27	3.98 (d, 3.6)	89.2d	C-12, C-25, C-28	H-26
28	4.39 (d, 5.2)	71.2d		H-29
29	2.48<br>1.75 (m)	46.6t	C-12, C-27, C-28	H-28
30	2.07 (s)	20.6q	C-15, C-16, C-17	H-17
31	0.99 (d, 6.8)	17.2q	C-19, C-20, C-21	H-20

32	1.02 (d, 7.0)	11.3q	C-21, C-22, C-23	H-22
33	0.85 (d, 7.0)	10.3q	C-23, C-24, C-25	H-24
34	0.83 (d, 7.0)	12.6q	C-25, C-26, C-27	H-26

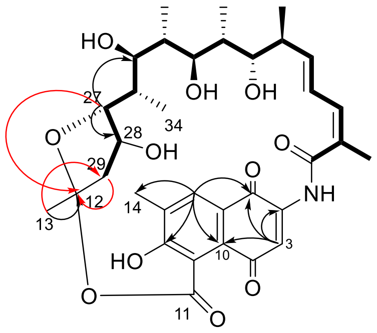

2

Table S3. NMR spectroscopic data for **3** in CD3OD (*δ* in ppm, *J* in Hz)

No.	δ_H (mult.)	δ_C mult.	HMBC	^{1}H-^{1}H COSY
1		181.3s		
2		142.3s		
3	7.67 (s)	117.9d	C-1, C-2, C-10	
4		187.2s		
5		nd[a]		
6		160.3s		
7		133.1s		
8	7.93 (s)	132.0d	C-1, C-6, C-10, C-14	
9		124.5s		
10		130.7s		
11				
12		211.0s		
13	2.17 (s)	31.3q	C-12, C-29	
14	2.35 (s)	17.6q	C-6, C-7, C-8	
15		170.4s		
16		130.1s		
17	6.47 (d, 11.0)	139.1d	C-15, C-19, C-30	H-18, H-30
18	6.80 (dd, 11.2, 15.0)	128.2d	C-17, C-20	H-17, H-19
19	6.04 (dd, 8.0, 15.0)	146.6d	C-17	H-18, H-20
20	2.41 (m)	43.0d	C-19, C-21	H-21, H-31
21	3.78 (dd, 0.7, 8.9)	76.3d	C-19, C-20, C-23, C-32	H-20, H-22
22	1.90 (m)	37.3d	C-32	H-21, H-32
23	3.50 (dd, 4.6, 7.8)	79.6d	C-21, C-24, C-25, C-33	H-22, H-24
24	1.84 (m)	39.1d	C-23, C-33	H-23, H-33
25	4.05 (dd, 2.0, 11.3)	84.2d	C-23, C-26, C-33, C-34	H-24, H-26
26	2.01 (m)	44.8d		H-25, H-27, H-34
27	3.44 (q, 8.5)	83.4d	C-29, C-34	H-26, H-28
28	3.96 (dq, 3.4, 8.6)	81.5d	C-12	H-27, H-29
29	2.76	49.1t	C-12, C-28	H-28
	2.65 (dq, 3.5, 15.6)			
30	2.07 (s)	21.1q	C-15, C-16, C-17	
31	0.99 (d, 6.2)	17.9q	C-19, C-20, C-21	H-20
32	0.97 (d, 5.8)	11.6q	C-21, C-22, C-23	H-22
33	0.95 (d, 6.6)	11.3q	C-23, C-24, C-25	H-24
34	1.04 (d, 6.5)	14.9q	C-25, C-26, C-27	H-26

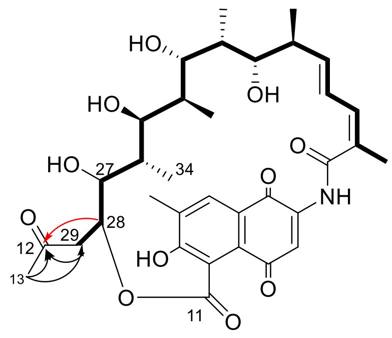

3

Table S4. NMR spectroscopic data for **4** in CD_3OD (*δ* in ppm, *J* in Hz)

No	δ_H (mult.)	δ_C mult.	HMBC	^1H-^1H COSY
1		181.9s		
2		143.1s		
3	7. 67 (s)	116.7d	C-1,C-2,C-9	
4		nd[a]		
5		nd[a]		
6		161.3s		
7		133.5s		
8	7.92 (s)	132.1d	C-1,C-6,C-9,C-13	
9		136.4s		
10		nd[a]		
11		200.2s		
12		143.3s		
13	2.32 (s)	17.5q	C-11, C-12, C-29	H-29
14	1.97 (s)	12.8q	C-6, C-7, C-8	
15		170.8s		
16		133.0s		
17	6.52 (d, 11.3)	141.7d	C-15, C-19, C-30	H-18
18	7.13 (dd, 11.3, 15.1)	129.4d	C-17, C-19	
19	6.07 (dd, 10.2, 15.2)	146.3d	C-17, C-31	H-18, H-20
20	2.49 (m)	48.2d		H-31
21	3.88 (m)	78.0d	C-19	H-20, H-22
22	1.65 (m)	43.8d		H-23, H-32
23	3.27 (dd, 3.1, 9.9)	82.1d		H-22, H-24
24	1.93 (m)	34.5d		H-33
25	3.97 (d, 8.6)	73.7d	C-34	H-26
26	1.48 (m)	41.8d		H-25, H-34
27	3.17 (t, 10.2)	74.2d	C-29, C-34	H-26, H-28
28	2.51 (m)	49.8d		H-29, H-34a
29	5.75 (d, 8.2)	145.8d	C-11, C-14	H-14, H-28
30	4.46 (m)	65.7t	C-15, C-16, C-17	
	4.24 (m)			
31	1.14 (d, 6.9)	20.3q	C-19, C-20, C-21	H-20
32	0.66 (d, 7.0)	12.2q	C-21, C-22, C-23	H-22
33	1.06 (d, 7.0)	12.1q	C-23, C-24, C-25	H-24
34	0.80 (d, 6.4)	12.9q	C-25, C-26, C-27	H-26
34a	5.12 (d, 4.1)	94.8d	C-25	H-28

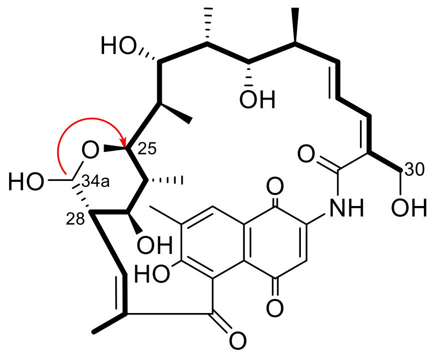

4

Table S5. NMR spectroscopic data for **5** in CD_3OD (δ in ppm, *J* in Hz)

No.	δ_H (mult.)	δ_C mult.	HMBC	^{1}H-^{1}H COSY
1		181.1s		
2		124.7s		
3	7. 60 (s)	118.9d	C-1, C-5, C-10	
4		188.0s		
5		129.3s		
6		164.0s		
7		133.6s		
8	7.96 (s)	132.5d	C-1, C-6, C-10, C-14	
9		126.9s		
10		132.9s		
11		201.1s		
12		142.6s		
13	2.11 (s)	13.2q	C-11, C-12, C-29	H-29
14	2.36 (s)	17.7q	C-6, C-7, C-8	
15		173.5s		
16		132.5s		
17	6.24 (d, 10.8)	135.6d	C-15, C-19, C-30	H-18
18	6.49 (dd, 11.0, 15.9)	126.9d	C-17, C-20	H-17
19	6.08 (dd, 6.7, 15.9)	142.0d	C-17, C-20, C-21	H-18, H-20
20	2.31 (m)	39.8d		H-19, H-21
21	4.03 (m)	75.5d	C-32	H-20, H-22
22	1.86 (m)	35.0d	C-32	H-32
23	3.48 (dd, 1.9, 10.4)	79.5d	C-21	H-24
24	1.78 (m)	38.7d	C-23	H-23, H-33
25	3.97 (m)	71.9d	C-23, C-24, C-26, C-33	H-26
26	1.38 (m)	44.5d	C-25, C-34	H-25, H-34
27	4.30 (s)	69.2d	C-25, C-26, C-28, C-29, C-34, C-34a	H-26, H-28
28	2.86 (m)	46.8d	C-12, C-29, C-34a	H-29, H-34a
29	6.26 (dd, 1.1, 10.4)	140.5d	C-11, C-13	H-28
30	2.09 (s)	20.9q	C-15, C-16, C-17	H-17
31	0.91 (d, 6.9)	18.7q	C-19, C-20, C-21	H-20
32	1.05 (d, 7.0)	11.8q	C-21, C-22, C-23	H-22
33	0.71 (d, 6.8)	9.5q	C-23, C-24, C-25	H-24
34	0.38 (d, 7.0)	12.3q	C-25, C-26, C-27	H-26
34a	4.00, 4.01 (m)	66.4t	C-27, C-35	H-28
35		173.0s		
36	2.03 (s)	21.5q	C-35	

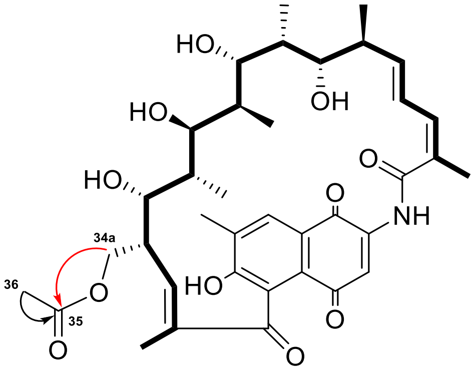

5

Table S6. NMR spectroscopic data for **6** in CD_3OD (δ in ppm, *J* in Hz)

No.	δ_H (mult.)	δ_C mult.	HMBC	^1H-^1H COSY
1		nd[a]		
2		nd[a]		
3	7.61 (s)	118.7d	C-1, C-5, C-10	
4		nd[a]		
5		nd[a]		
6		160.6s		
7		133.1s		
8	7.97 (s)	132.0d	C-1, C-6, C-10, C-14	
9		nd[a]		
10		nd[a]		
11		201.3s		
12		138.5s		
13	2.04 (d,1.0)	12.2q	C-11, C-12, C-29	H-29
14	2.37 (s)	17.5q	C-6, C-7, C-8	
15		173.3s		
16		133.5s		
17	6.28 (d, 11.1)	135.0d	C-18, C-30	
18	6.53 (dd, 11.2, 16.6)	128.9d		H-17,H-19
19	6.01 (dd, 6.4, 15.2)	137.8d	C-18, C-20	H-18,H-20
20	2.43 (m)	48.1d		H-19, H-21, H-31
21	4.24 (dd, 2.0, 8.6)	72.2d		H-20, H-22
22	1.94 (m)	35.5d		H-21, H-23, H-32
23	3.46 (dd, 2.3, 9.9)	79.4d		H-22, H-24
24	1.77 (m)	38.7d		H-23, H-33
25	3.95 (d, 9.6)	71.6d	C-33	H-26
26	1.40 (m)	44.1d		H-25, H-34
27	3.98 (s)	74.5d	C-29, C-34, C-34a	H-28
28	2.61 (m)	41.4d		H-29, H-34a
29	6.42 (dd, 2.0, 9.2)	147.0d	C-11, C-13	H-13, H-28
30	2.08 (s)	20.6q	C-15, C-16, C-17	H-17
31	3.52, 3.53 (d, 4.8)	63.9t	C-19, C-21	H-20
32	1.07 (d, 3.2)	12.5q	C-21, C-22, C-23	H-22
33	0.73 (d, 6.8)	9.3q	C-23, C-24, C-25	H-24
34	0.36 (d, 7.0)	11.5q	C-25, C-26, C-27	H-26
34a	1.06 (d, 3.3)	20.0q	C-27, C-35	H-28

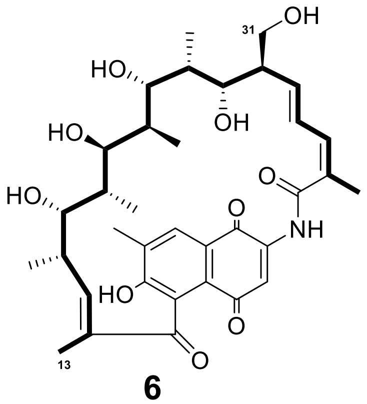

6

Table S7. NMR spectroscopic data for **7** in CD_3OD (*δ* in ppm, *J* in Hz)

No.	δ_H (mult.)	δ_C mult.	HMBC	^1H-^1H COSY
1		180.5s		
2		141.2s		
3	7.61 (s)	118.2d	C-1, C-2, C-10	
4		nd[a]		
5		nd[a]		
6		160.0s		
7		nd[a]		
8	7.97 (s)	131.6d	C-1, C-6, C-10, C-14	
9		nd[a]		
10		132.0s		
11		201.1s		
12		140.7s		
13	2.09 (s)	12.6q	C-11, C-12, C-29	H-29
14	2.36 (s)	17.1q	C-6, C-8, C-10	
15		172.7s		
16		133.4s		
17	6.29 (dd, 1.0, 11.0)	134.6d	C-15, C-30	H-18, H-30
18	6.53 (dd, 11.0, 15.9)	128.4d	C-20	H-17, H-19
19	6.01 (dd, 7.0, 15.8)	137.5d	C-17	H-18, H-20
20	2.42 (m)	47.4d		H-21, H-31
21	4.26 (1.8, 9.1)	71.7d	C-32	H-20, H-22
22	1.95 (m)	35.2d	C-32	H-32
23	3.47 (m)	79.1d	C-25	H-22, H-24
24	1.78 (m)	38.5d		H-23, H-33
25	3.96 (dd, 1.0, 10.2)	71.2d	C-33	H-24, H-26
26	1.40 (m)	44.0d		H-25, H-34
27	4.37 (m)	68.8d	C-25, C-28, C-29, C-34, C-34a	H-26, H-28
28	2.66 (m)	49.7d		H-29, H-34a
29	6.32 (dd, 1.1, 9.4)	141.9d	C-11, C-13	H-13, H-28
30	2.08 (s)	20.1q	C-15, C-16, C-17	H-17
31	3.54 (m)	63.5t	C-19, C-20, C-21	H-20
32	1.08 (d, 7.0)	12.0q	C-21, C-22, C-23	H-22
33	0.73 (d, 6.8)	8.8q	C-23, C-24, C-25	H-24
34	0.39 (d, 7.0)	11.7q	C-25, C-26, C-27	H-26
34a	3.56 (m)	64.3t		H-28

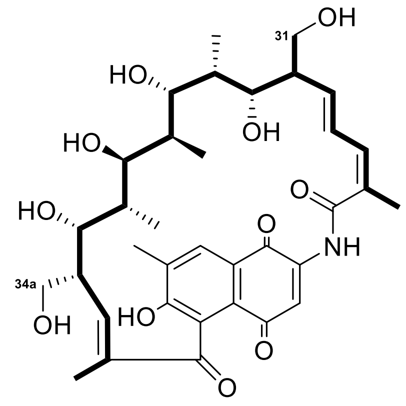

7

Table S8. NMR spectroscopic data for **8** in CD_3OD (δ in ppm, *J* in Hz)

No.	δH (mult.)	δC mult.	HMBC	^1H-^1H COSY
1		181.0s		
2		133.7s		
3	7.59 (s)	118.5d	C-1, C-5, C-10	
4		188.0d		
5		129.1s		
6		160.7s		
7		132.2s		
8	7.93 (s)	132.0d	C-1, C-7, C-10, C-14	H-14
9		124.2s		
10		142.5s		
11		201.2s		
12		141.7s		
13	2.10 (s)	13.0q	C-11, C-12, C-29, C-30	
14	2.38 (s)	17.5q	C-6, C-7, C-8	
15		170.9s		
16		134.3s		
17	6.51 (d, 10.9)	140.0d	C-15, C-18, C-19, C-30	H-18
18	6.90 (dd, 11.0, 16.1)	127.4d	C-16, C-17, C-20	H-19
19	6.36 (dq, 1.3, 7.4)	146.2d	C-17, C-20, C-21	H-18, H-20
20	2.32 (m)	39.3s	C-18, C-19, C-21, C-31	H-21, H-31
21	4.05 (d, 9.9)	76.6d	C-19, C-20, C-32	H-20, H-22
22	1.93 (m)	34.6d	C-32	H-21, H-23, H-32
23	3.49 (dd, 1.8, 10.3)	79.5d	C-21, C-24, C-32, C-33	H-22, H-24
24	1.81 (m)	38.4d	C-23, C-33	H-23, H-25, H-33
25	3.97 (dd, 1.2, 10.2)	71.7d	C-23, C-24, C-26, C-27, C-33	H-24, H-26
26	1.42 (m)	44.5d	C-25, C-34	H-25, H-34
27	4.38 (d, 6.6)	69.6d	C-25, C-26, C-28, C-29, C-34, C-34a	H-26, H-28
28	2.69 (q, 7.2, 16.6)	50.3d	C-29, C-30	H-27, H-29, H-34a
29	6.34 (d, 6.4)	142.0d	C-11, C-12, C-13, C-27	H-13, H-28
30	4.23, 4.36 (dq, 12.1)	65.4t	C-15, C-16, C-17	
31	0.94 (d, 7.0)	18.2q	C-19, C-21	H-20
32	1.06 (d, 7.0)	11.3q	C-21, C-22, C-23	H-22
33	0.74 (d, 6.8)	9.4q	C-23, C-24, C-25	H-24
34	0.42 (d, 7.0)	11.9q	C-25, C-26, C-27	H-26
34a	3.43, 3.61 (dq, 7.9, 10.9)	65.0t	C-27, C-28, C-29	H-28

nd[a]: not observed and/or not defined

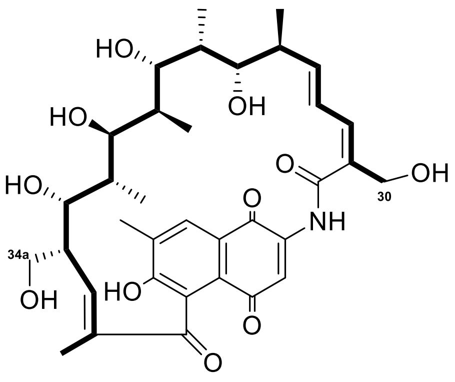

8

Table S9. NMR spectroscopic data for **9** in CD_3OD (δ in ppm, *J* in Hz)

No.	δ_H (mult.)	δ_C mult.	HMBC	^{1}H-^{1}H COSY
1		181.0s		
2		142.0s		
3	7.58 (s)	119.0d	C-1, C-2, C-10	
4		188.0s		
5		nd[a]		
6		160.7s		
7		132.3s		
8	7.94 (s)	132.4d	C-1, C-7, C-9, C-10, C-14	H-14
9		129.1s		
10		132.5s		
11		201.1s		
12		142.2s		
13	2.08 (s)	13.2q	C-11, C-12, C-29	H-29
14	2.35 (s)	17.7q	C-6, C-7, C-8	
15		172.2s		
16		133.4s		
17	6.24 (dd, 0.8,10.8)	135.9d	C-15, C-18, C-19, C-30	H-18, H-30
18	6.45 (dd, 10.9, 15.9)	126.0d	C-16, C-17, C-21	H-17, H-19
19	5.95 (d, 16.0)	148.2d	C-17, H-18, C-20, C-31	H-18
20		76.9d		
21	3.94 (br d, 0.8)	76.6d	C-19, C-22, C-23, C-32	H-22
22	2.00 (m)	35.2d	C-32	H-21, H-23, H-32
23	3.42 (d, 6.8)	80.7d	C-21, C-22, C-24, C-32, C-33	H-22, H-24
24	1.71 (m)	38.9d	C-23, C-33	H-23, H-33
25	3.92 (dd, 1.0, 10.2)	72.2d	C-23, C-24, C-26, C-27, C-33	H-24, H-26
26	1.39 (m)	44.5d	C-25, C-34	H-25, H-34
27	4.35 (br s)	69.7d	C-25, C-26, C-28, C-29, C-34, C-34a	H-26, H-28
28	2.65 (dq, 1.2, 7.9)	50.3d	C-12, C-29, C-34a	H-27, H-29, H-34a
29	6.29 (dd, 0.7, 9.5)	142.5d	C-11, C-13, C-27, C-28, C-34a	H-28
30	2.09 (s)	20.9q	C-15, C-16, C-17	H-17
31	1.02 (s)	26.7q	C-19, C-20, C-21	
32	1.17 (d, 7.0)	14.5q	C-21, C-22, C-23	H-22
33	0.74 (d, 6.8)	9.7q	C-23, C-24, C-25	H-24
34	0.40 (d, 7.0)	12.3q	C-25, C-26, C-27	H-26
34a	3.40, 3.59 (dq, 1.8, 9.7)	65.0t	C-27, C-28, C-29	H-28

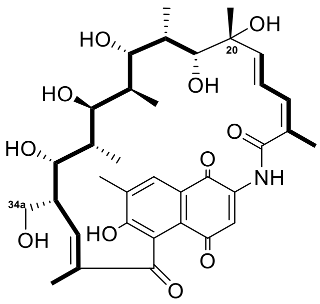

9

Table S10. NMR spectroscopic data for **10** in CD_3OD (*δ* in ppm, *J* in Hz)

No.	δ_H (mult.)	δ_C mult.	HMBC	^{1}H-^{1}H COSY
1		181.0s		
2		142.6s		
3	7.59 (s)	119.2d	C-1, C-2, C-10	H-14
4		nd[a]		
5		nd[a]		
6		161.4s		
7		131.5s		
8	7.92 (s)	132.0d	C-1, C-6, C-10, C-14	
9		nd[a]		
10		133.0s		
11		201.4s		
12		142.2s		
13	2.07 (s)	13.2q	C-11, C-12, C-29	
14	2.34 (s)	17.5q	C-6, C-7, C-8	
15		173.3s		
16		134.3s		
17	6.26 (dd, 1.1, 10.9)	133.3d	C-15, C-16, C-30	H-30
18	6.13 (dd, 11.0, 15.1)	130.4d	C-16, C-20	H-17
19	5.82 (dd, 12.4, 15.1)	136.6d	C-17, C-31	H-18, H-20
20	1.85 (m)	52.7d	C-18, C-19, C-31	H-19
21	3.81 (m)	73.9d	C-19, C-22, C-32	H-20, H-22
22	2.93 (m)	50.0d	C-21, C-32	H-21, H-32
23		211.3s	C-24, C-25	
24	2.45 (m)	50.8d	C-33	H-25, H-33
25	3.86 (m)	71.5d	C-26, C-27, C-33	H-24, H-26
26	1.32 (m)	42.8d	C-25	H-25, H-34
27	4.41 (m)	68.9d	C-25, C-26, C-28, C-29, C-34, C-34a	H-26, H-28
28	2.56 (m)	49.7d	C-29, C-34a	H-34a
29	6.23 (dd, 1.4, 9.2)	141.3d	C-11, C-13, C-27	H-28
30	2.04 (s)	20.9q	C-15, C-16, C-17	H-17
31	4.37, 4.23 (m)	65.1t	C-19	
32	1.03 (d, 6.8)	15.5q	C-21, C-22	H-22
33	1.13 (d, 7.4)	8.5q	C-24, C-25	H-24
34	0.44 (d, 7.0)	12.2q	C-25, C-26, C-27	H-26
34a	3.50, 3.39 (m)	65.0t	C-27, C-28, C-29	H-28

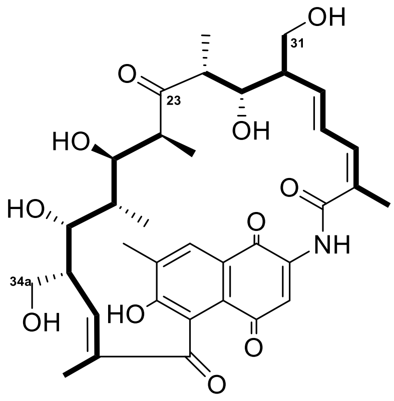

10

Table S11. NMR spectroscopic data for **11** in CD_3OD (*δ* in ppm, *J* in Hz)

No.	δ_H (mult.)	δ_C mult.	HMBC	^{1}H-^{1}H COSY
1		179.7s		
2		141.8s		
3	7.43 (s)	119.0d	C-1, C-2, C-10	
4		nd[a]		
5		nd[a]		
6		162.0s		
7		132.9s		
8	7.89 (s)	131.2d	C-1, C-6, C-10, C-14	
9				
10		133.0s		
11		200.3s		
12		142.9s		
13	2.04 (s)	12.5q	C-11, C-12, C-29	H-29
14	2.31 (s)	16.8q	C-6, C-7, C-8	
15		172.0s		
16		132.9s		
17	6.22 (m)	134.5d	C-15, C-19, C-30	H-30
18	6.29 (m)	124.3d	C-16, C-20	H-19
19	5.84 (d, 14.4)	142.8d	C-17, C-20	H-18
20		82.3s		
21	3.80 (d, 10.5)	86.4d	C-19, C-22, C-31, C-32	H-22
22	1.79 (m)	48.3d	C-21, C-23, C-32	H-21, H-32
23		106.8s		
24	1.91 (m)	43.0d	C-23, C-33	H-33
25	4.23 (dd, 0.7, 10.2)	73.8d	C-33	H-24, H-26
26	1.46 (m)	43.7d		H-25, H-27, H-34
27	4.28 (br s)	68.3d	C-25, C-26, C-28, C-29, C-34, C-34a	H-26
28	2.56 (m)	48.9d	C-29, C-34a	H-29, H-34a
29	6.25 (m)	142.0d	C-11, C-13	H-13, H-28
30	2.08 (s)	20.3q	C-15, C-16, C-17	H-17
31	1.31 (s)	28.7q	C-19, C-20, C-21	
32	1.13 (d, 6.5)	13.0q	C-21, C-22, C-23	H-22
33	0.97 (d, 7.2)	8.6q	C-23, C-24, C-25	H-24
34	0.55 (d, 8.1)	12.4q	C-25, C-26, C-27	H-26
34a	3.46, 3.35 (m)	64.8t		H-28

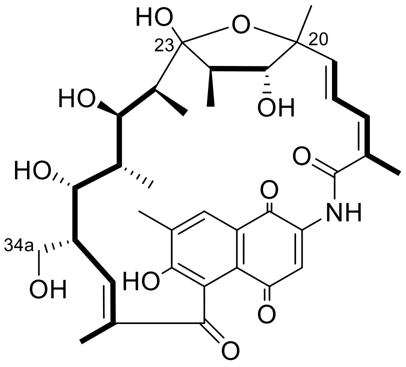

11

3. NMR and HRESIMS spectra of compounds 1–11

Figures S9–14. HRESIMS and NMR spectra of **1**

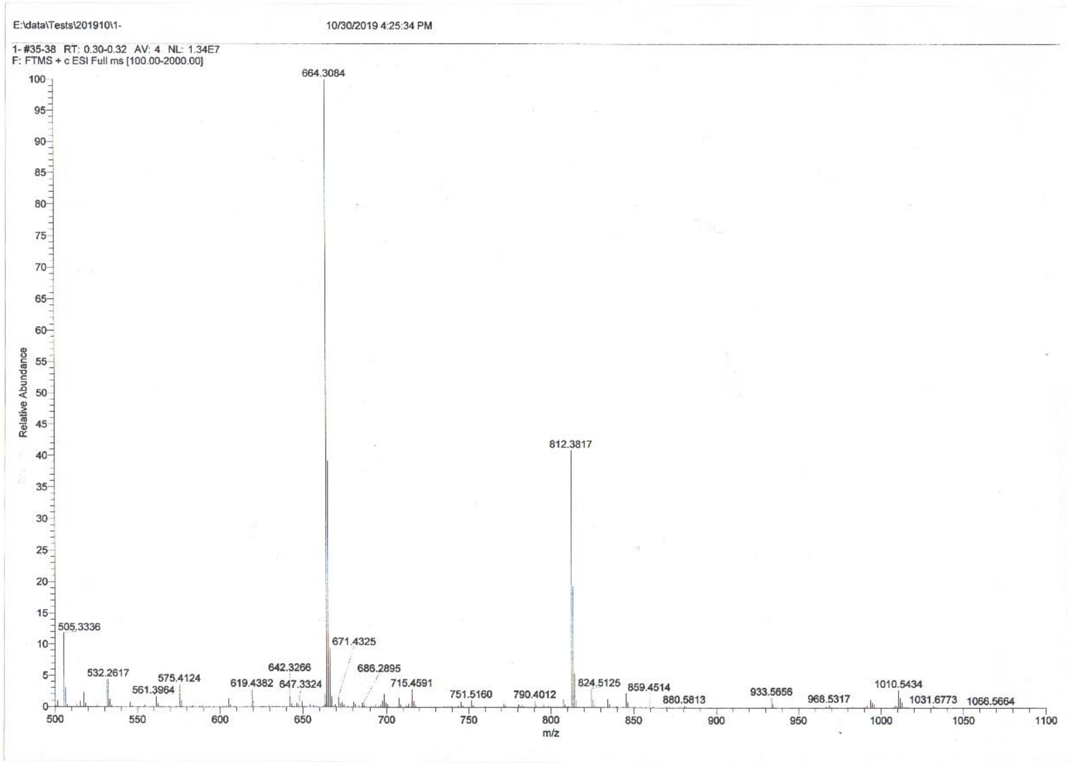

Figure S9. HRESIMS spectrum of **1**.

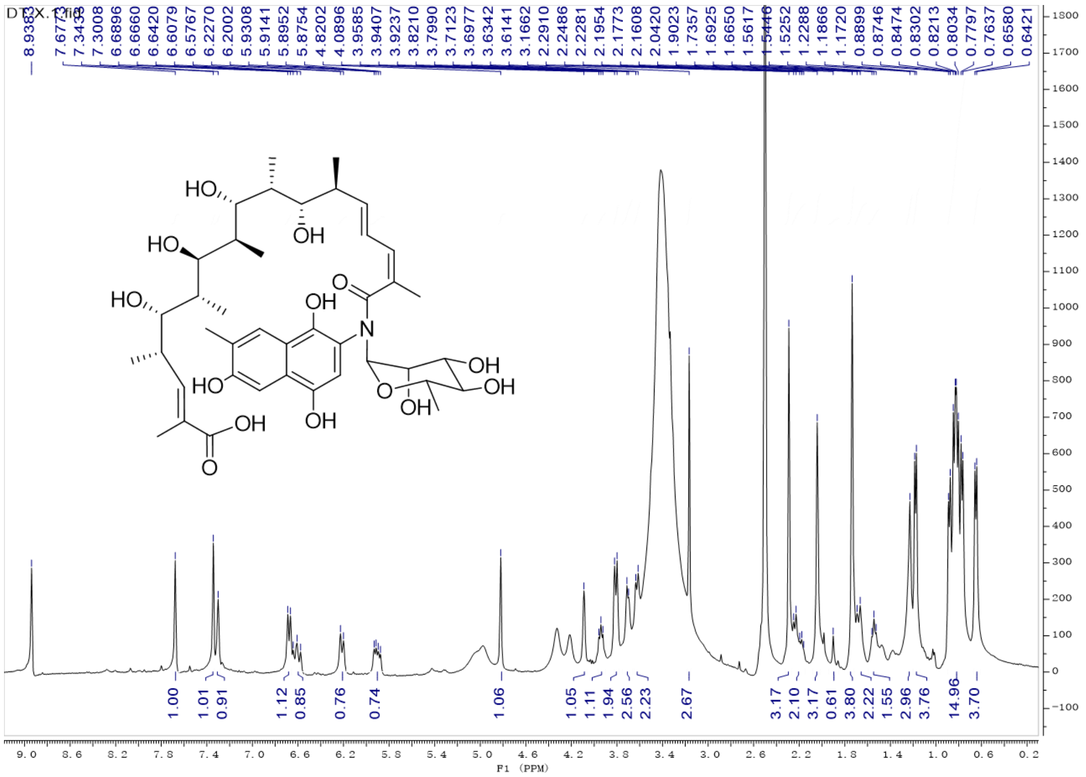

Figure S10. ^{1}H NMR spectrum of **1** in DMSO-d_6 (600 MHz).

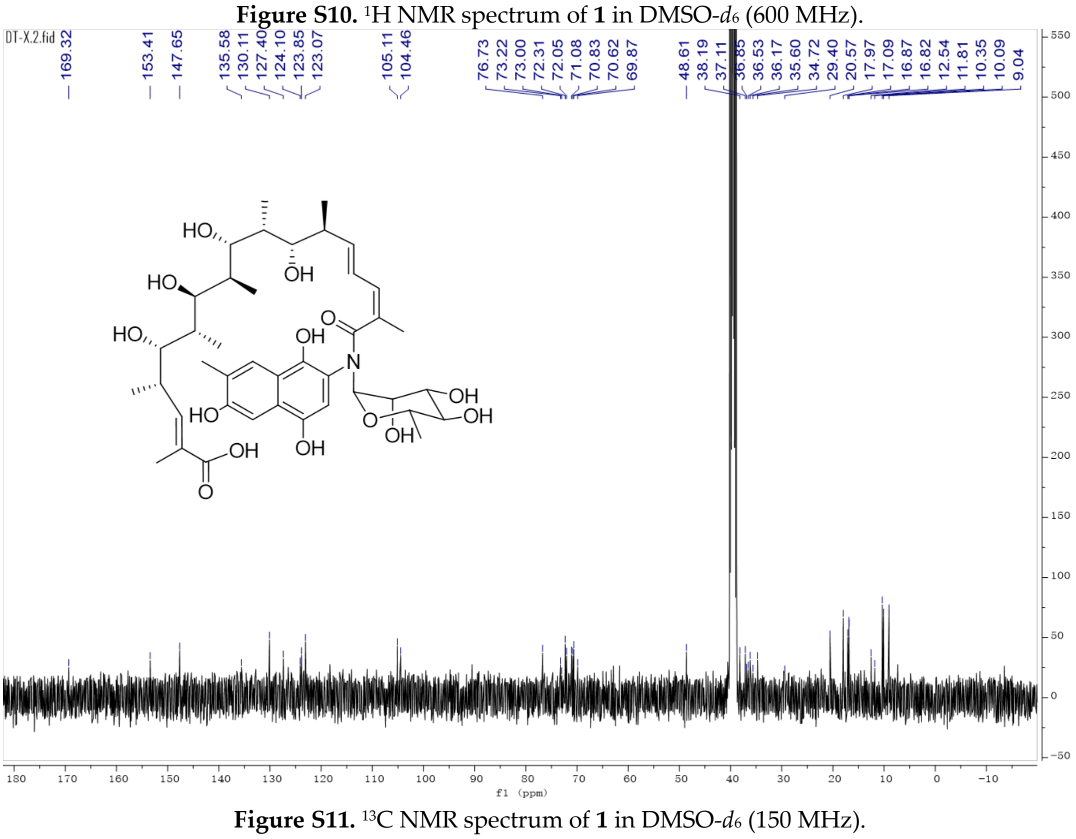

Figure S11. ^{13}C NMR spectrum of **1** in DMSO-d_6 (150 MHz).

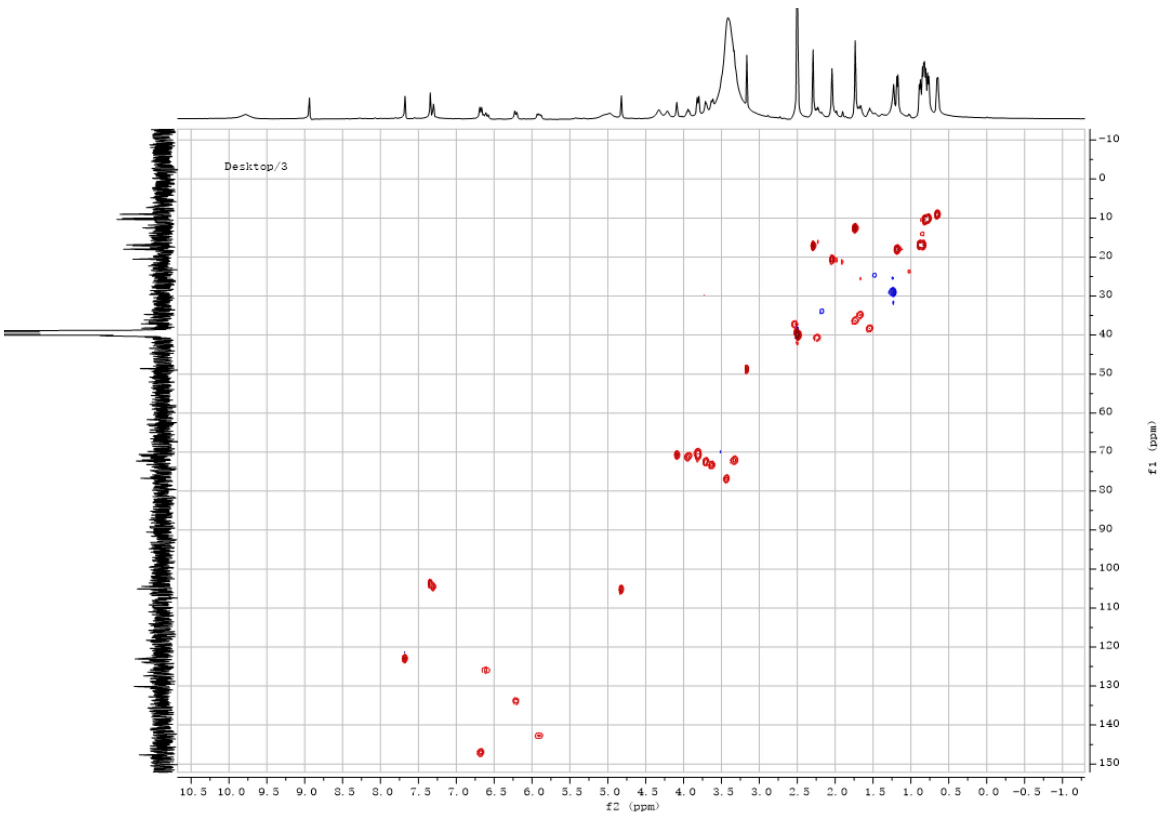

Figure S12. HSQC spectrum of **1** in DMSO-d_6.

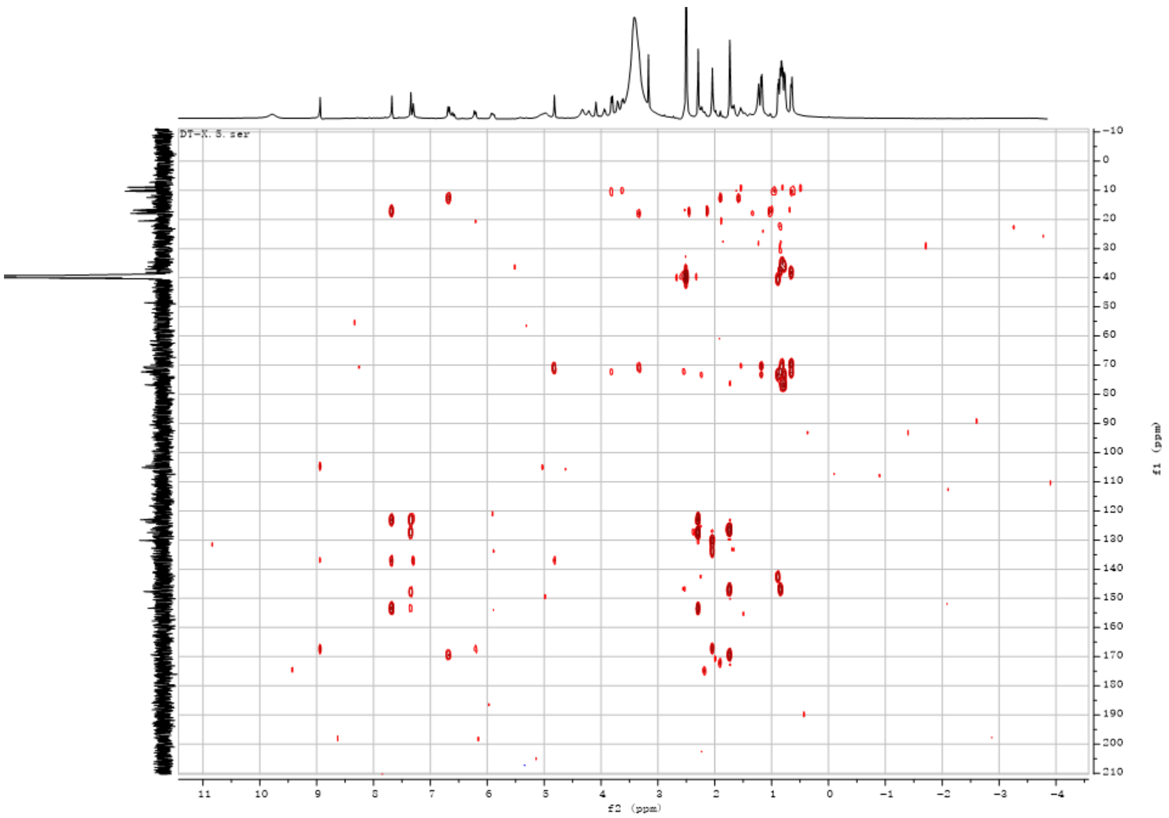

Figure S13. HMBC spectrum of **1** in DMSO-d_6.

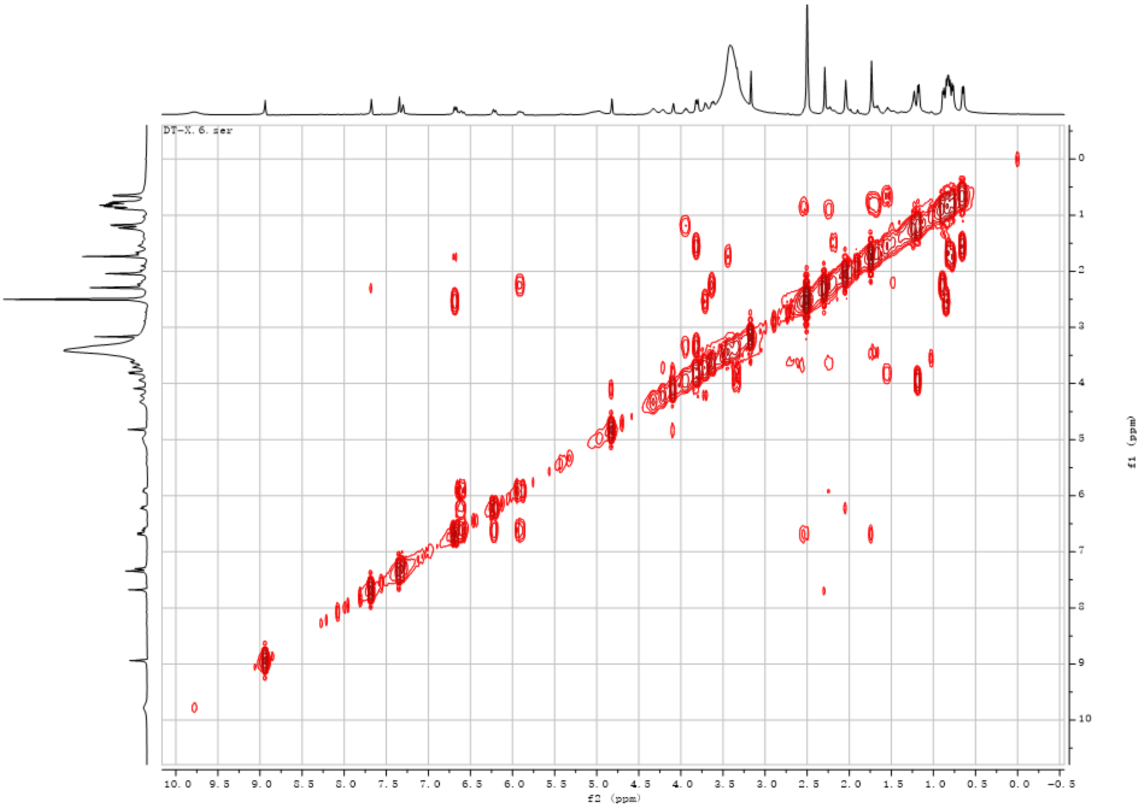

Figure S14. ¹H-¹H COSY spectrum of **1** in DMSO-d_6.

Figures S15-17. Analysis of sugar stereochemistry in compound 1

Due to the instability of amide *N*-glycoside, compound **1** occurs spontaneous hydrolysis of glycosidic bond and transform to corresponding aglycone proansamycin B-M1 and deoxyhexapyranose moiety. The process was recorded by HPLC as following:

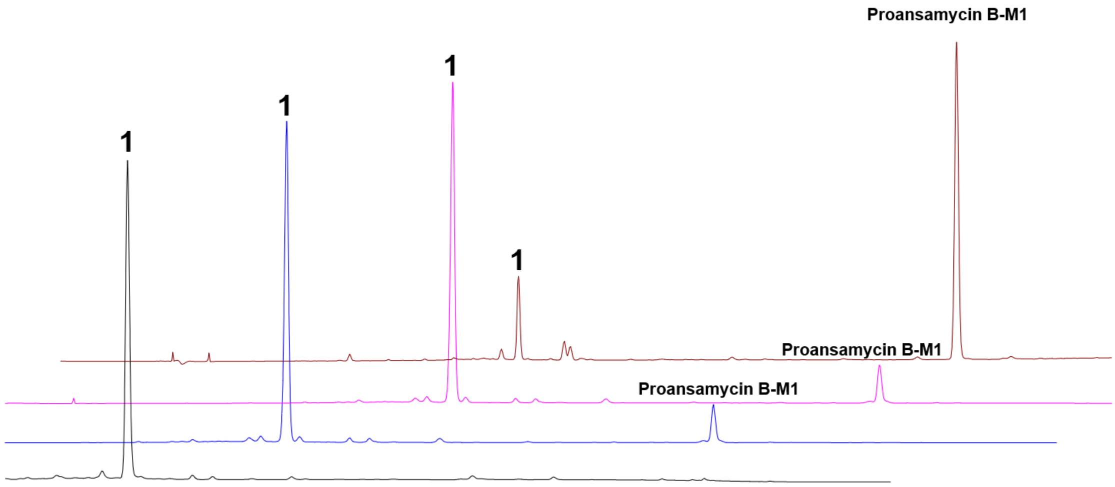

Figure S15. HPLC detection of degradation of compound **1**.

Compound **1** and its degradation mixture (10mg) was subjected to column chromatography (CC) over silica gel (CH_2Cl_2 : MeOH 10 : 1 ; 5:1 and 1:1) to afford Fr. 1A (from 10 : 1 eluent) , Fr. 1B (1.3 mg, from 5:1 eluent) and Fr. 1C (from 5:1 eluent). The components were detected by TLC as following：

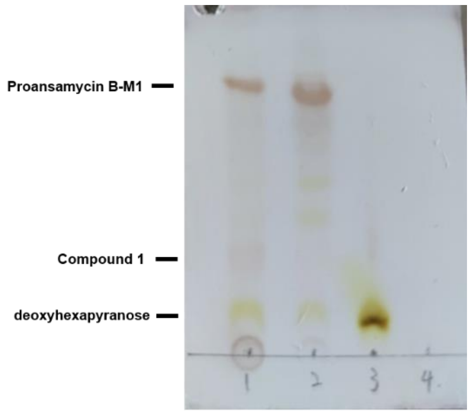

1: Original mixture
2: Fr1A (CH_2Cl_2 : MeOH 10 : 1)
3: Fr1B (CH_2Cl_2 : MeOH 5 : 1)
4: Fr1C (CH_2Cl_2 : MeOH 1 : 1)

Figure S16. TLC detection of compound **1** degradation mixture and components Fr. 1A–C.

Comparison of ^{1}H NMR of deoxyhexapyranose moiety with α-L-rhamnose standard.

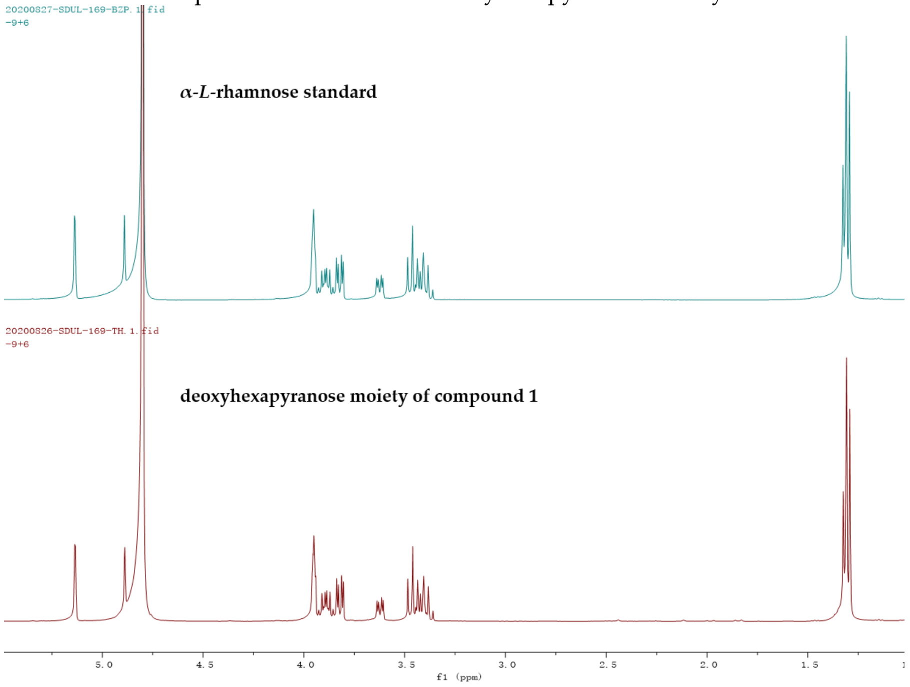

Figure S17. Comparison of ^{1}H NMR of deoxyhexapyranose moiety with α-*L*-rhamnose standard in D_2O.

Figures S18–23. HRESIMS and NMR spectra of **2**

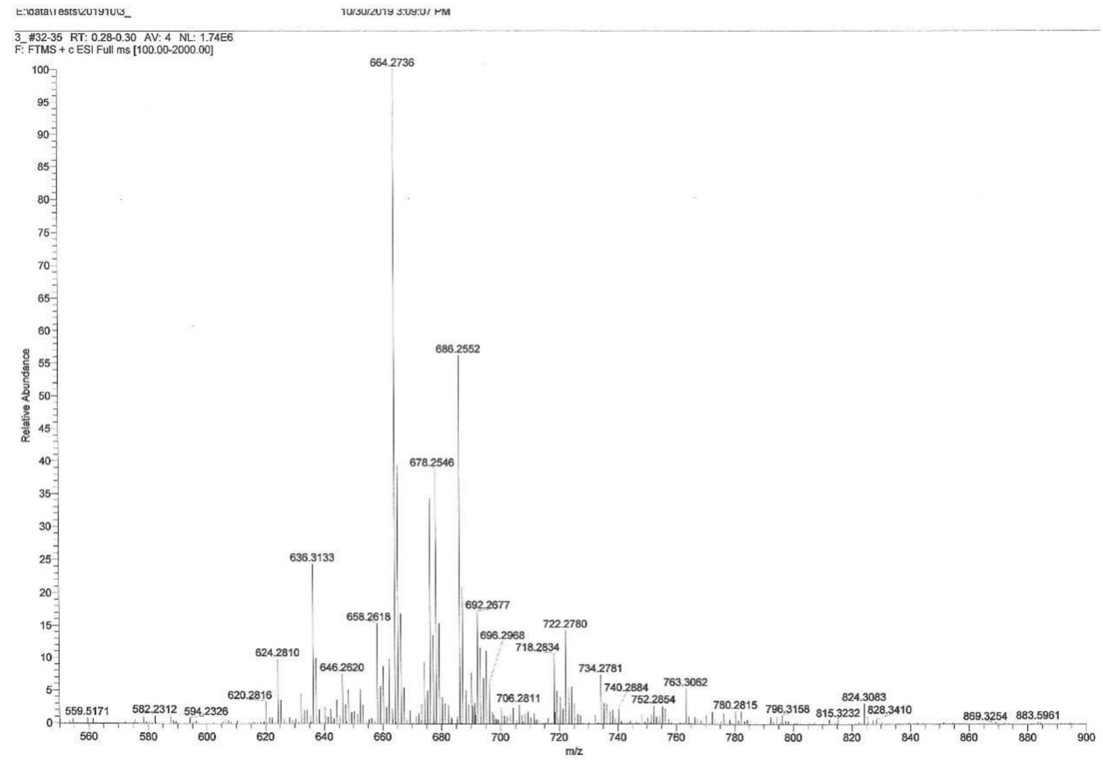

Figure S18. HRESIMS spectrum of **2**.

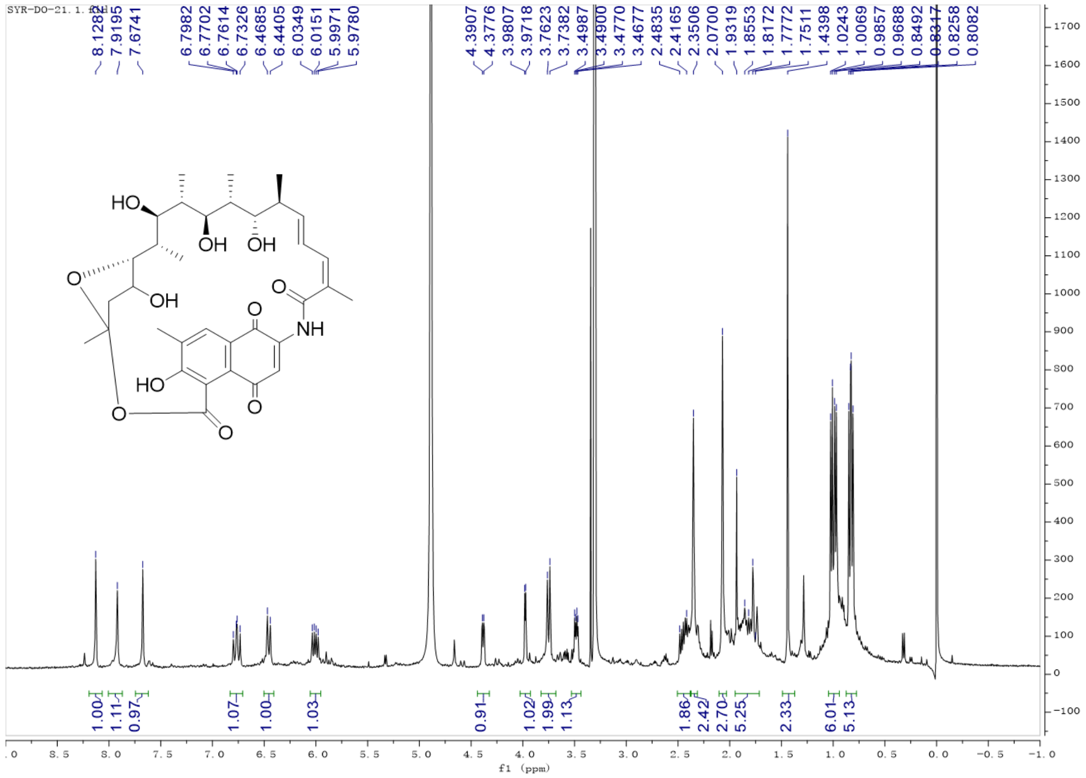

Figure S19. ^{1}H NMR spectrum of **2** in CD_3OD (400 MHz).

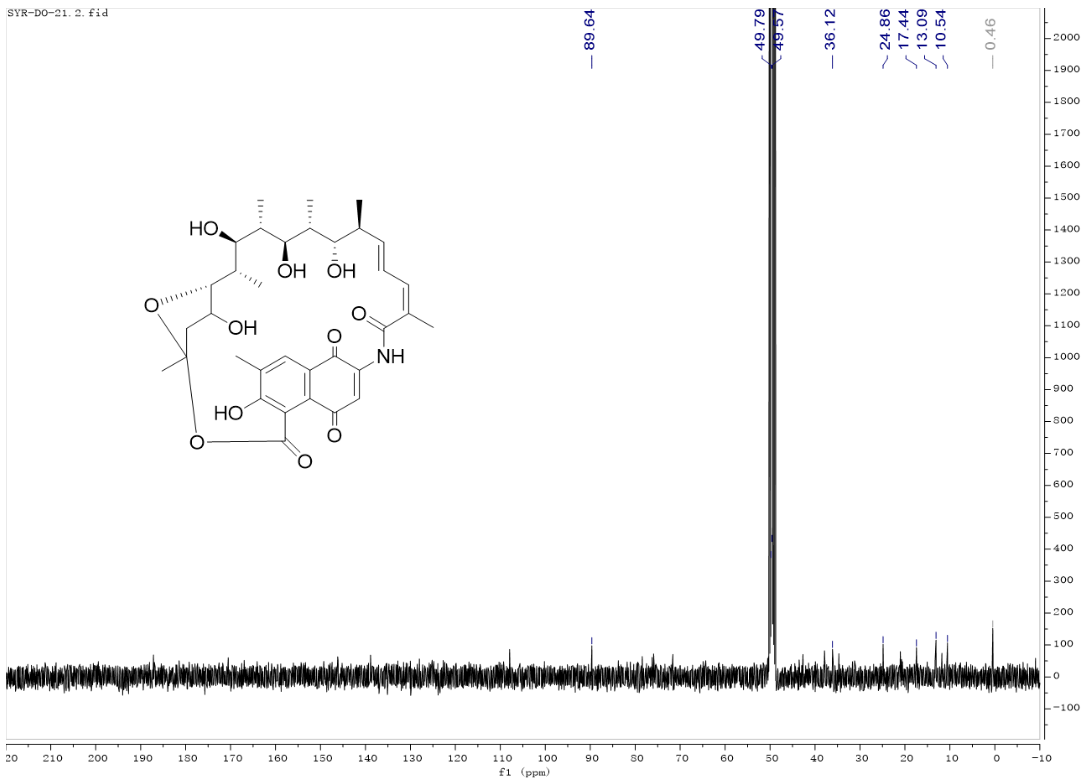

Figure S20. ^{13}C NMR spectrum of **2** in CD_3OD (100 MHz).

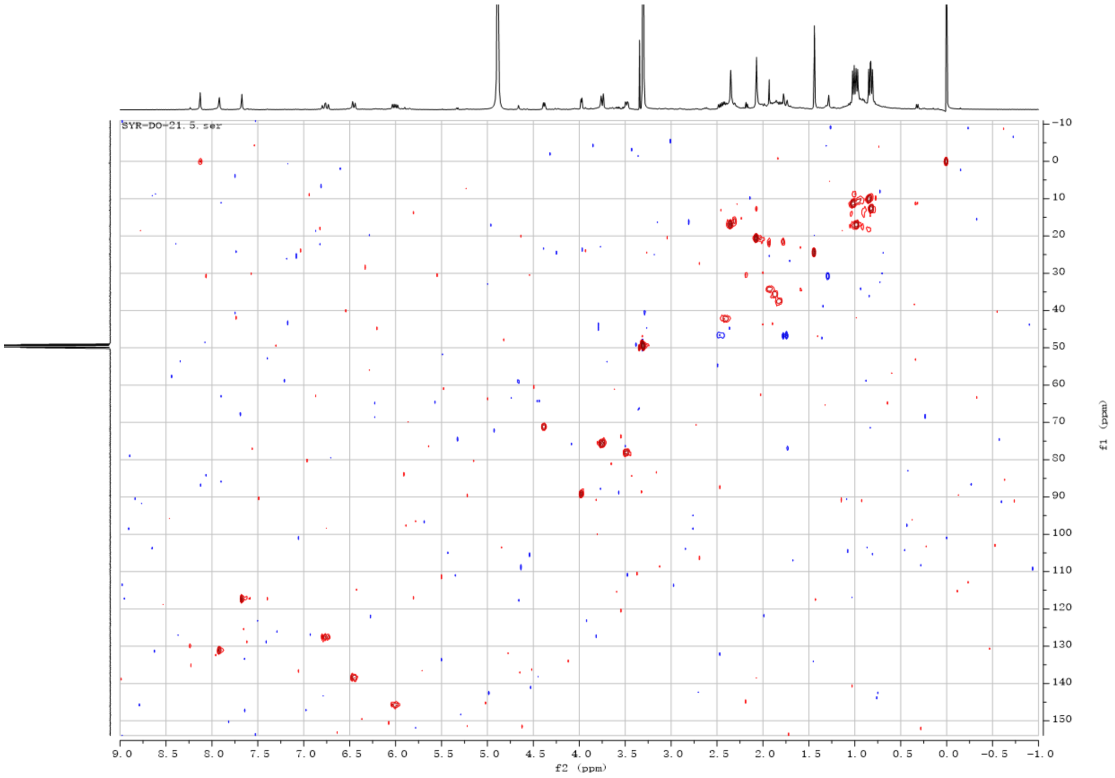

Figure S21. HSQC spectrum of **2** in CD_3OD.

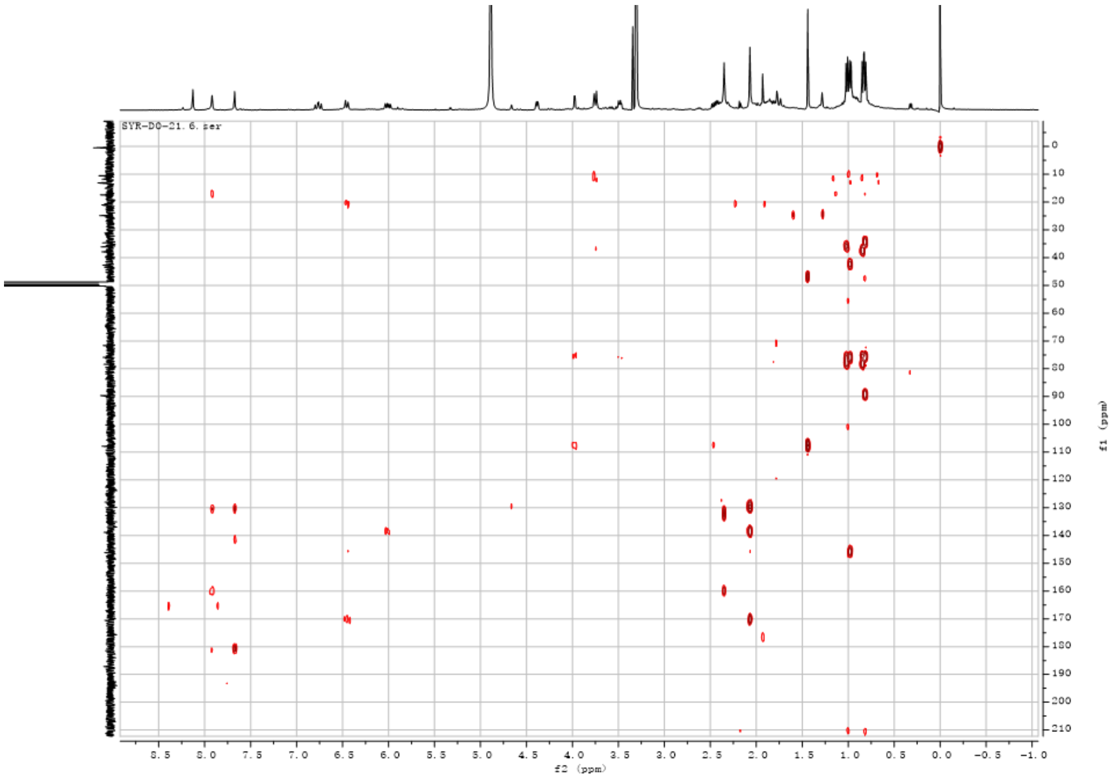

Figure S22. HMBC spectrum of **2** in CD_3OD.

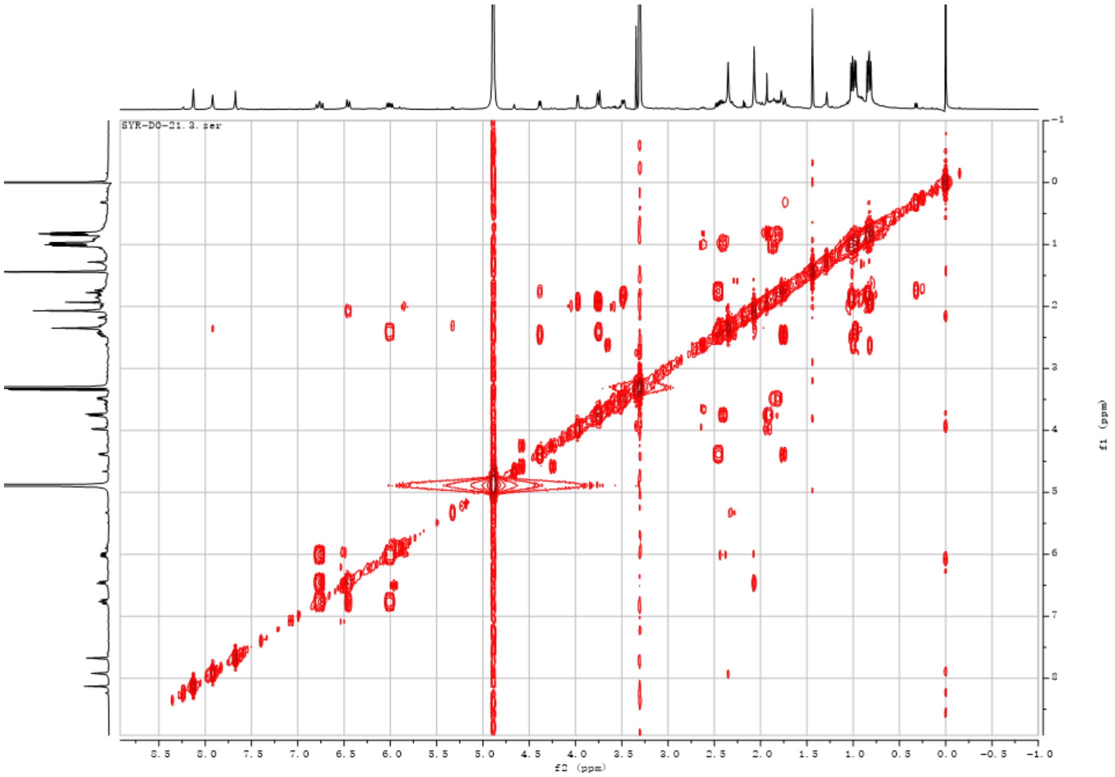

Figure S23. ^1H-^1H COSY spectrum of **2** in CD_3OD.

Figures S24–29. HRESIMS and NMR spectra of **3**

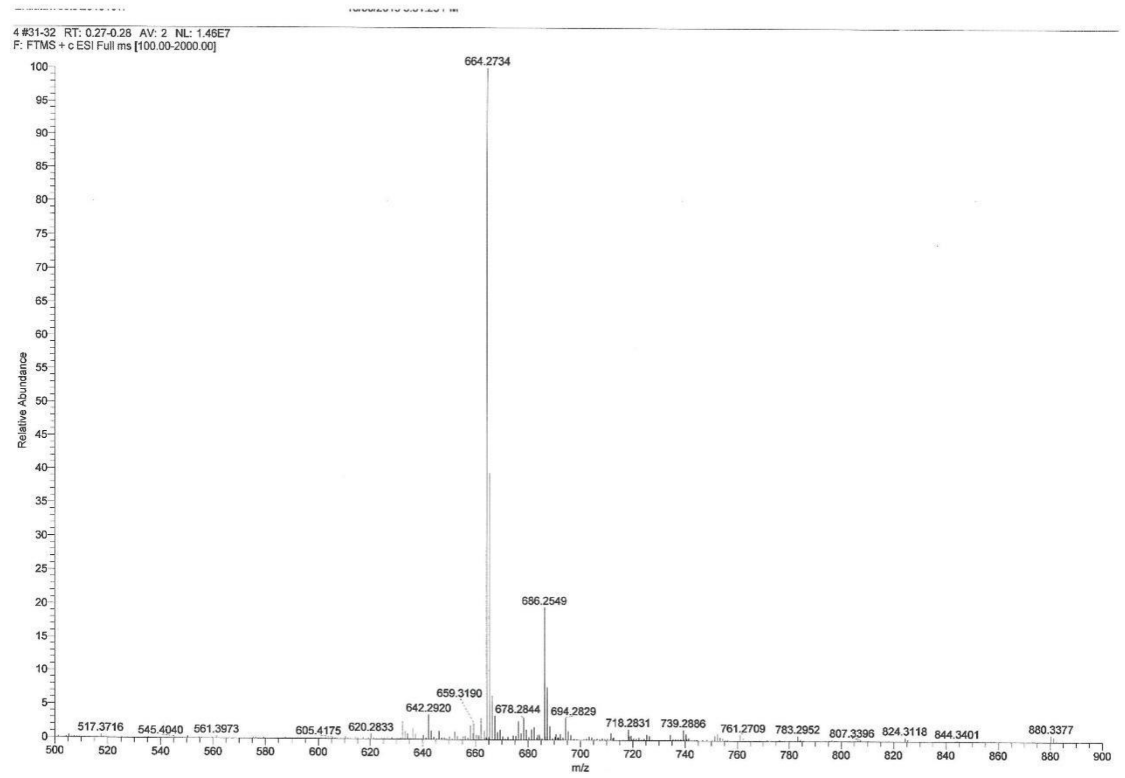

Figure S24. HRESIMS spectrum of **3**.

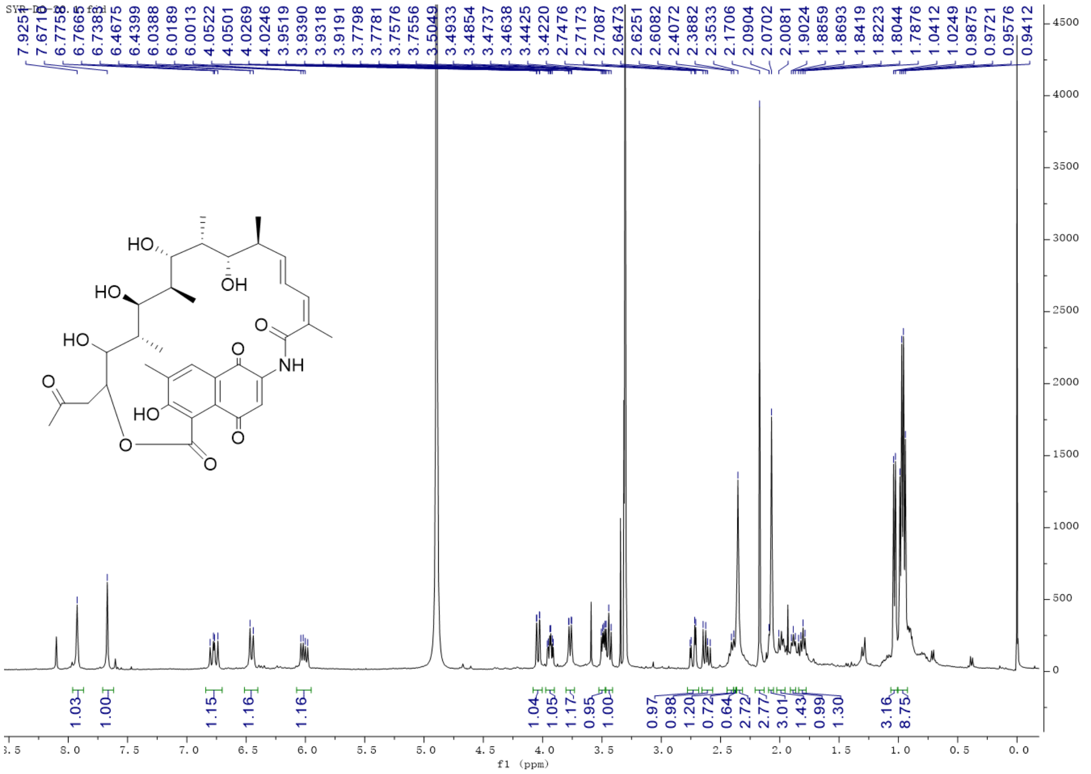

Figure S25. ^{1}H NMR spectrum of **3** in CD_3OD (400 MHz).

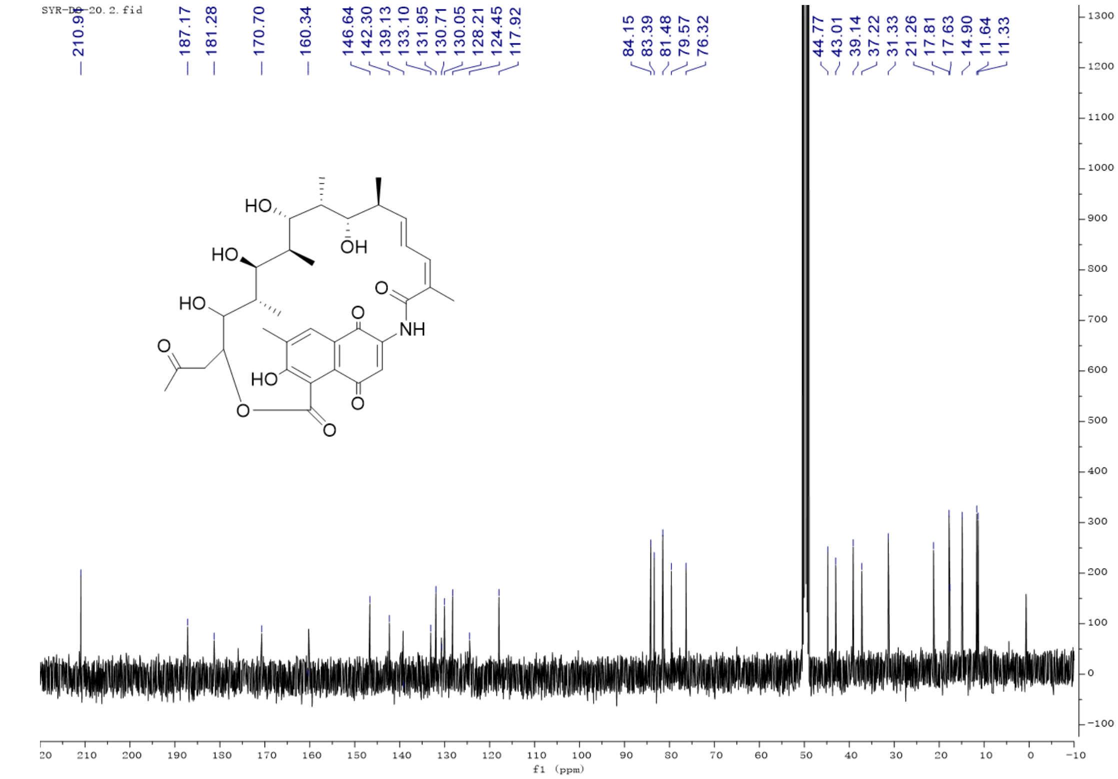

Figure S26. ^{13}C NMR spectrum of **3** in CD_3OD (100 MHz).

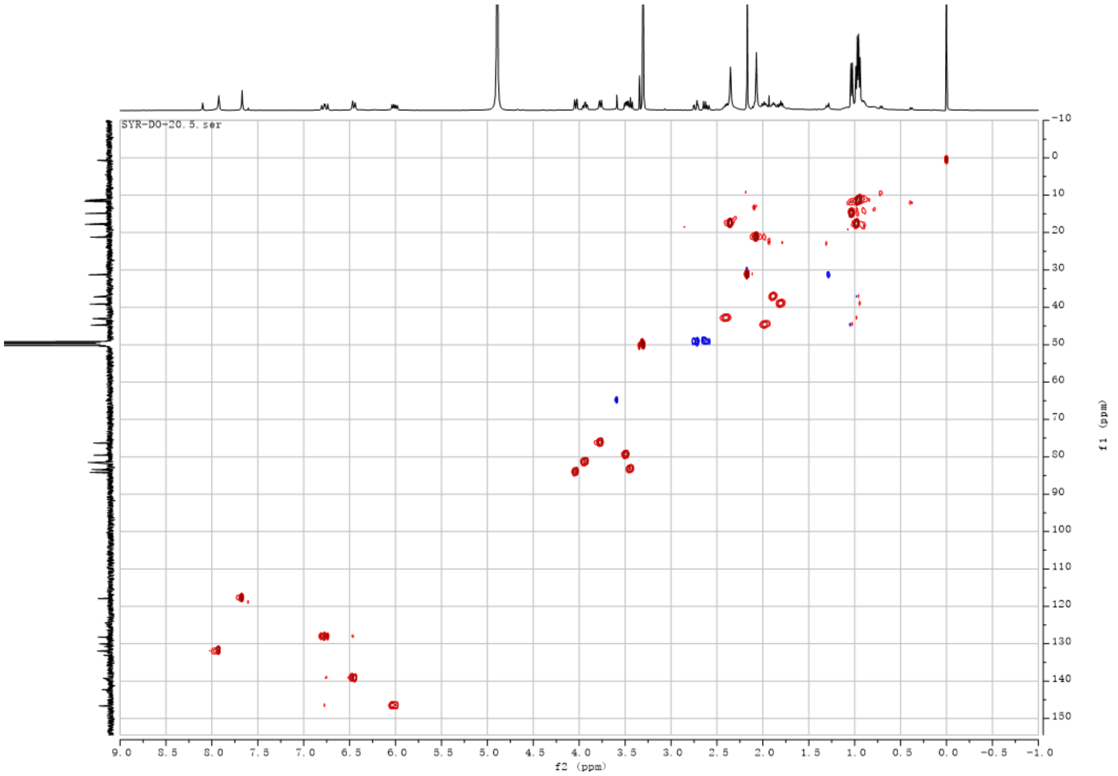

Figure S27. HSQC spectrum of **3** in CD_3OD.

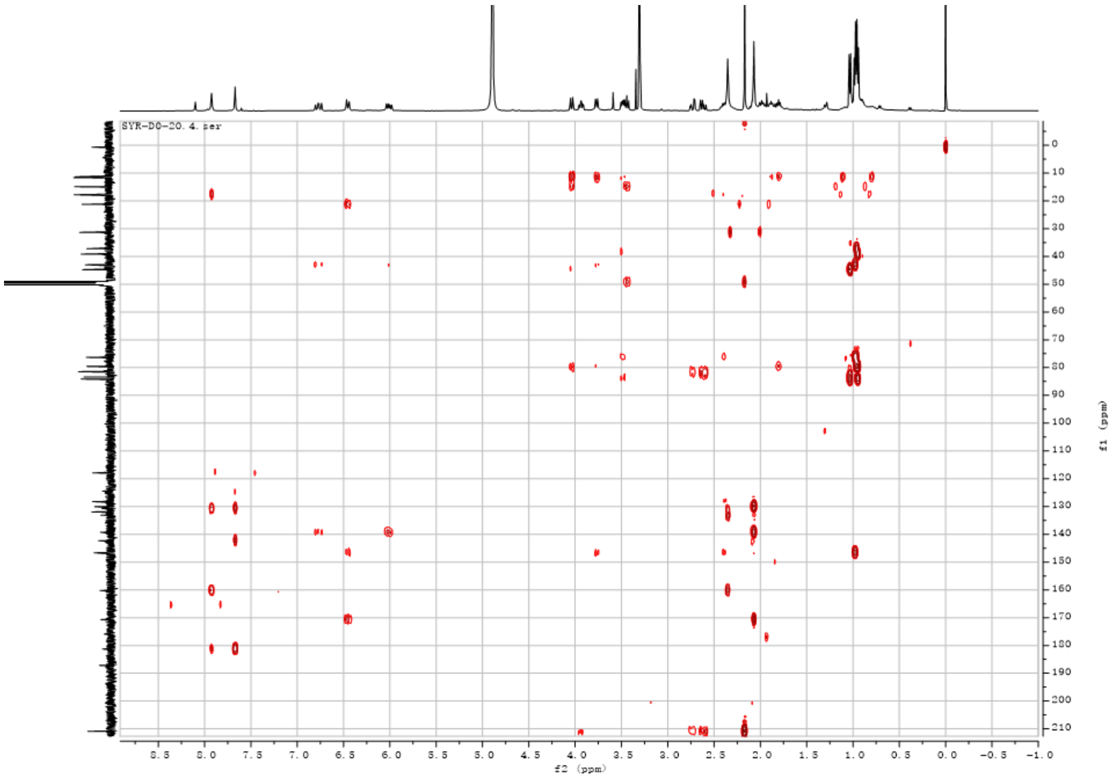

Figure S28. HMBC spectrum of **3** in CD_3OD.

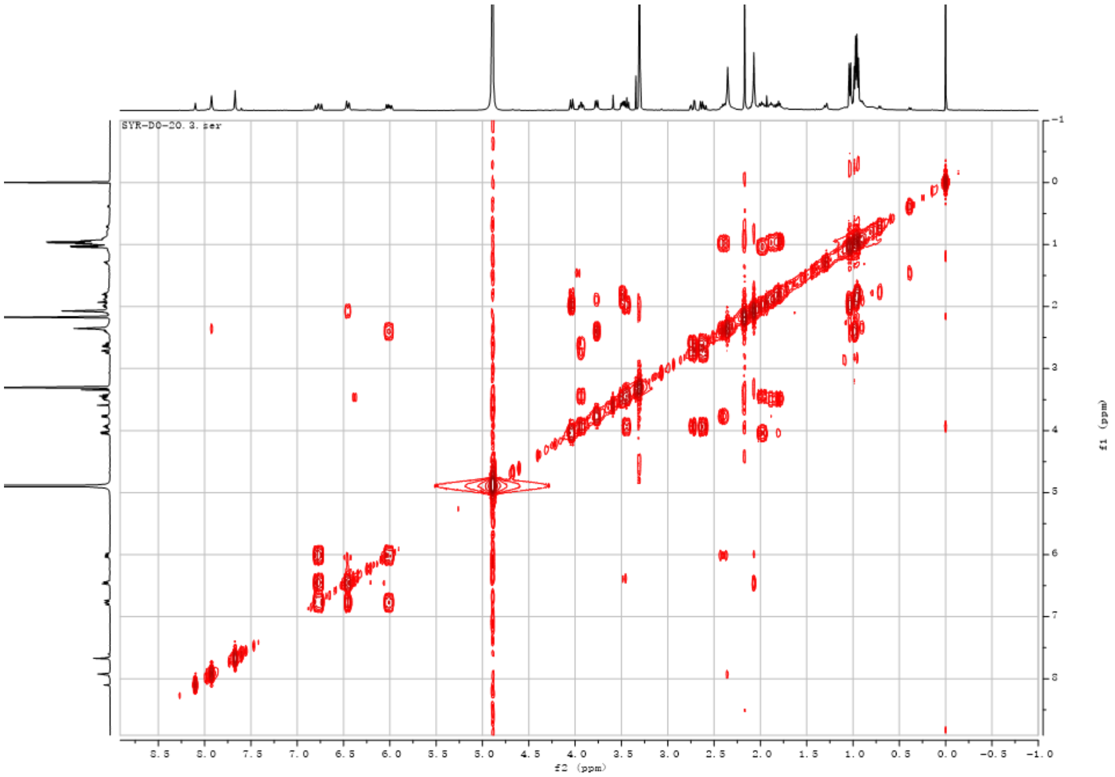

Figure S29. ^1H-^1H COSY spectrum of **3** in CD_3OD.

Figures S30–35. HRESIMS and NMR spectra of **4**

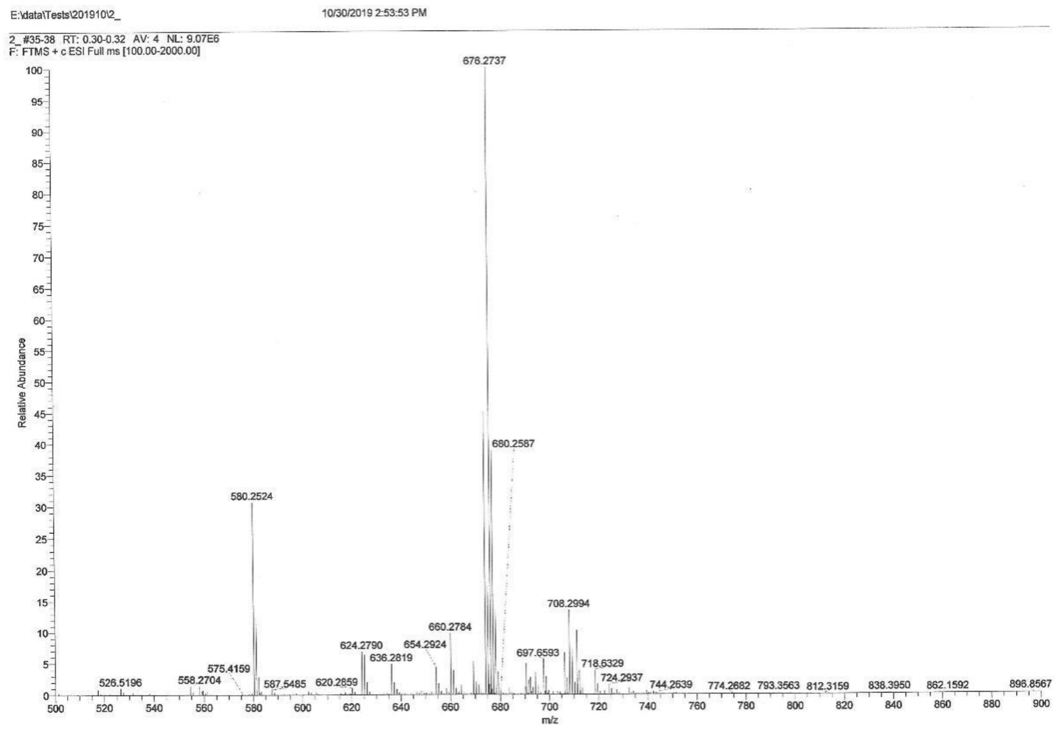

Figure S30. HRESIMS spectrum of **4**.

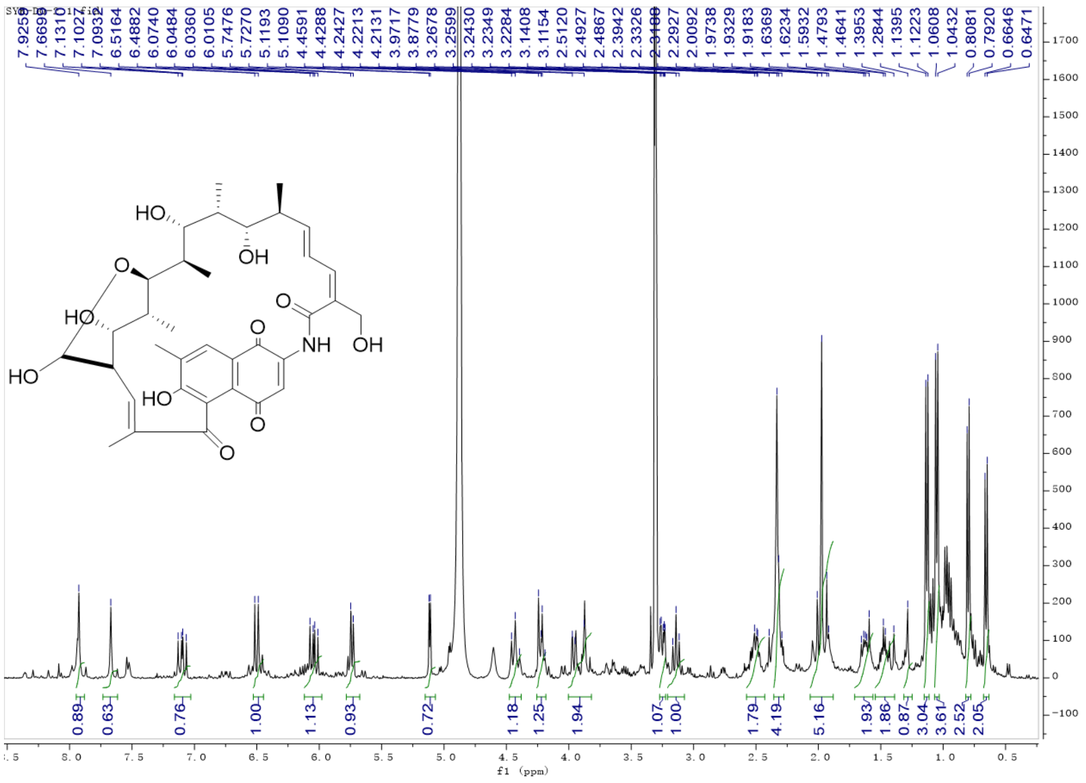

Figure S31. ^{1}H NMR spectrum of **4** in CD_3OD (400 MHz).

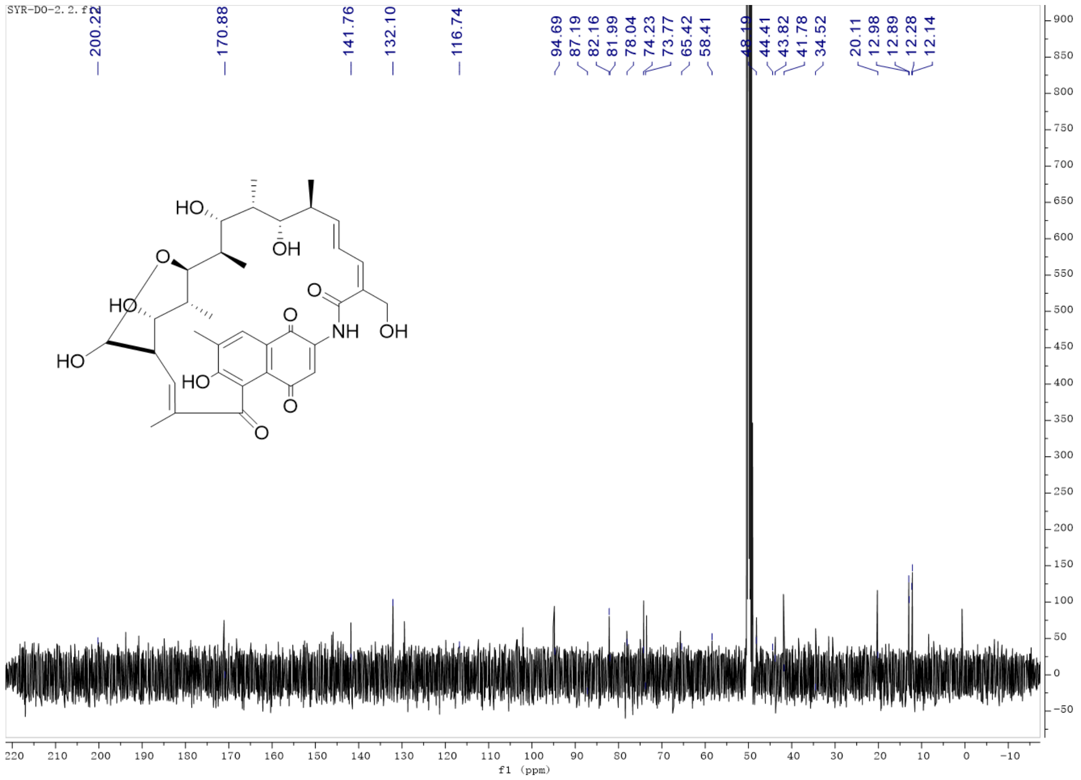

Figure S32. ^{13}C NMR spectrum of **4** in CD_3OD (100 MHz).

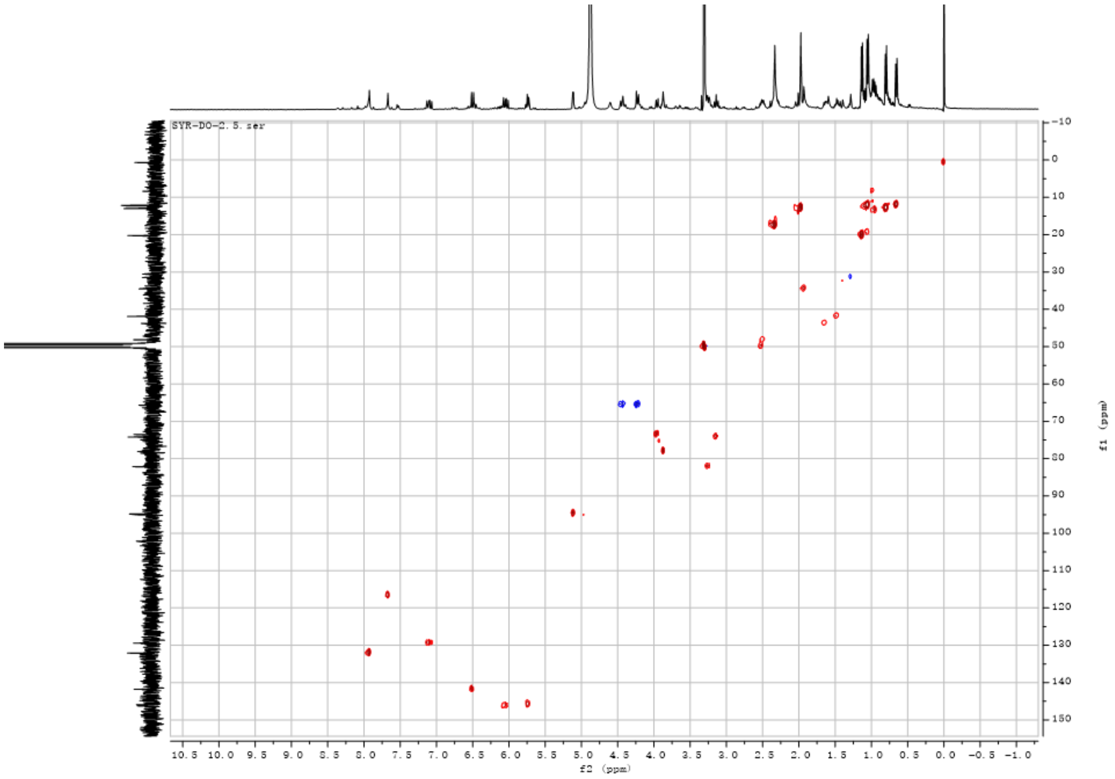

Figure S33. HSQC spectrum of **4** in CD_3OD.

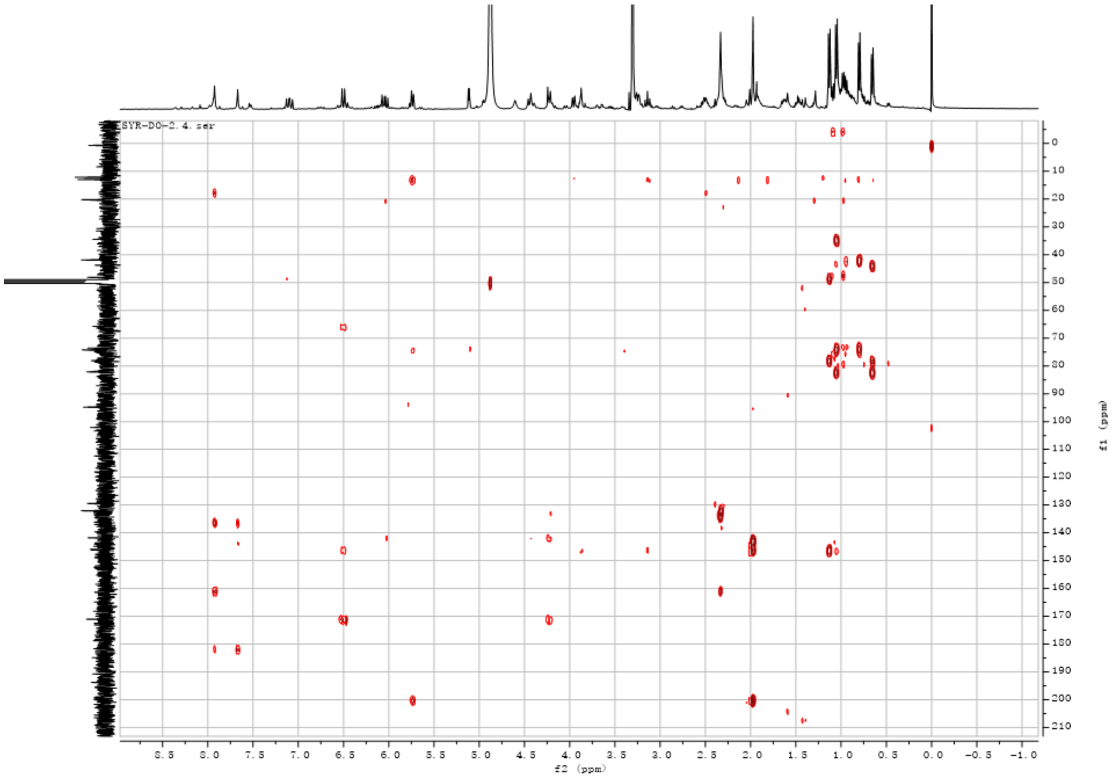

Figure S34. HMBC spectrum of **4** in CD_3OD.

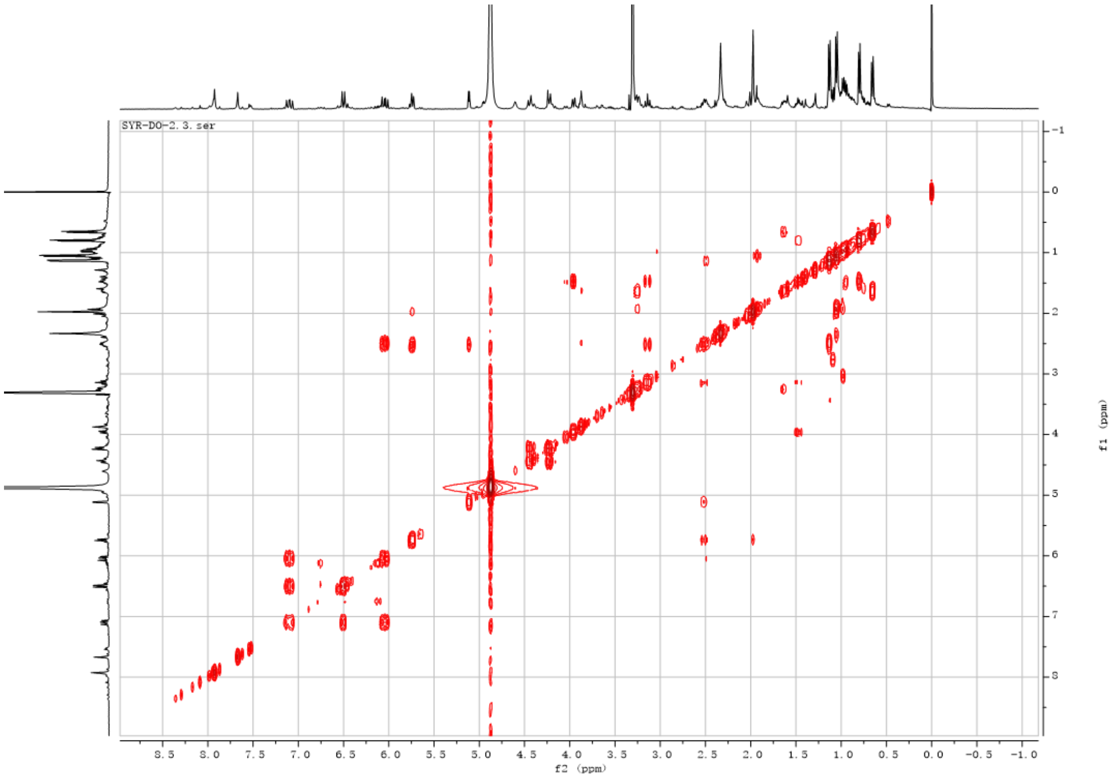

Figure S35. ^1H-^1H COSY spectrum of **4** in CD_3OD.

Figures S36–50. HRESIMS and NMR spectra of **5**

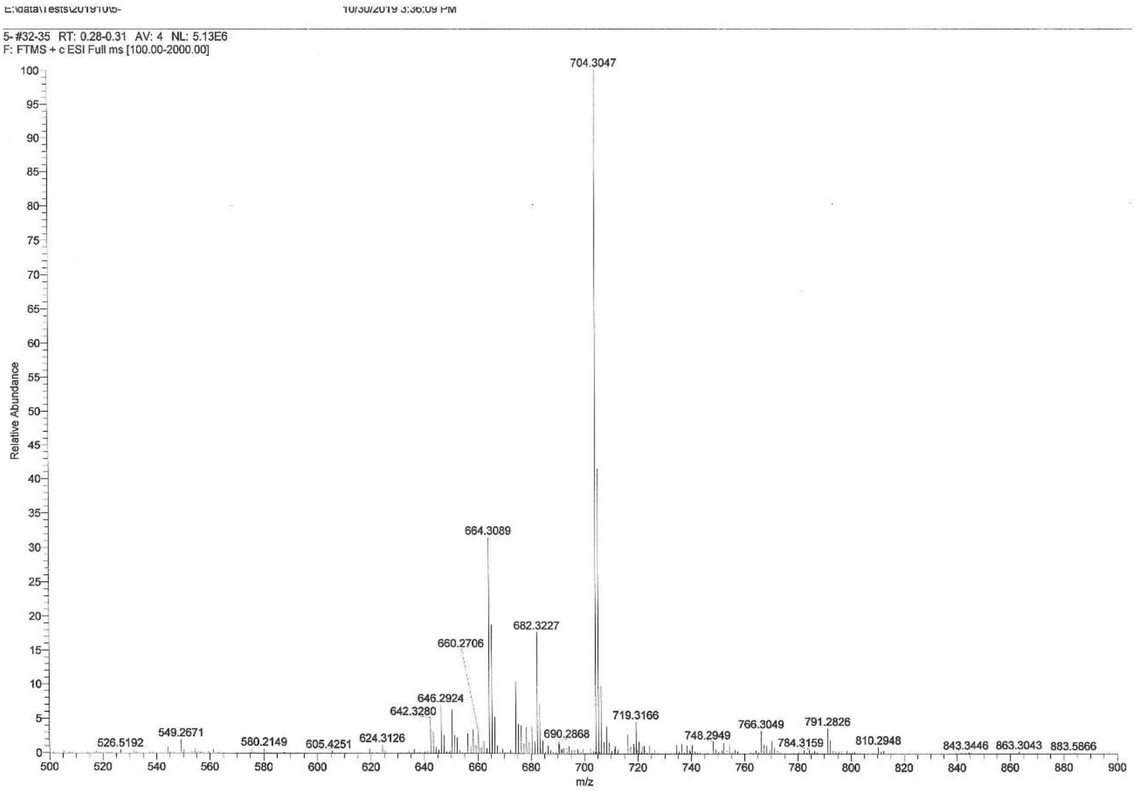

Figure S36. HRESIMS spectrum of **5**.

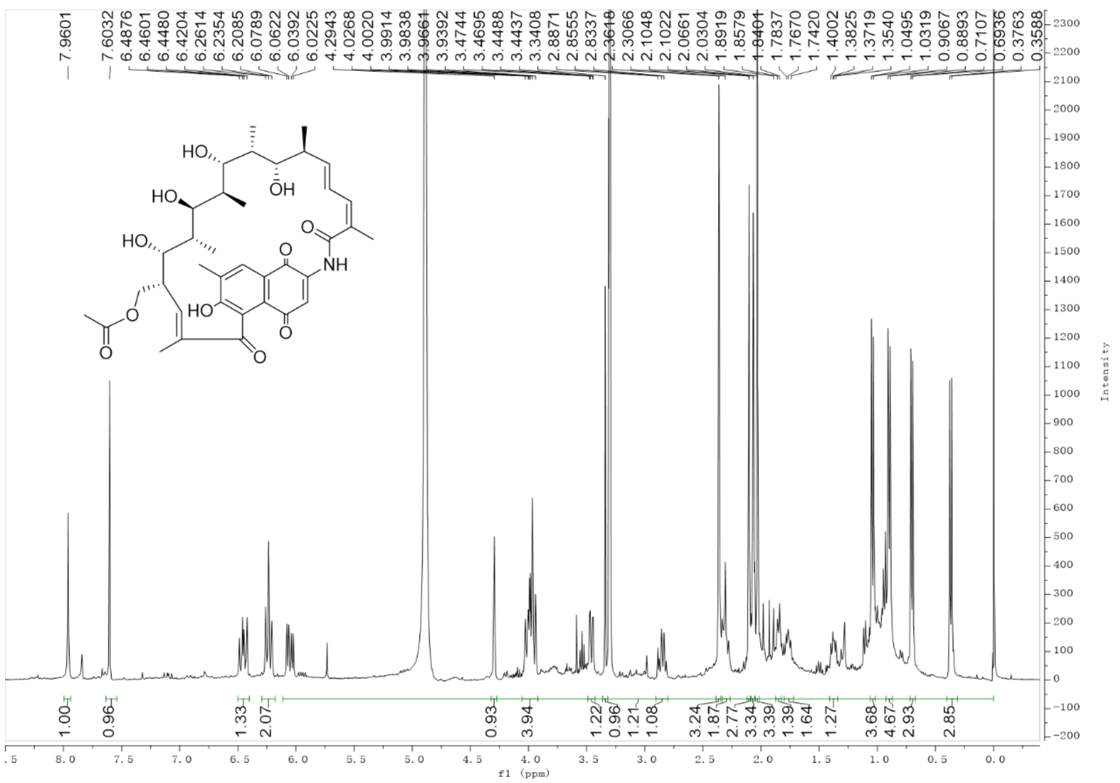

Figure S37. ^{1}H NMR spectrum of **5** in CD_3OD (400 MHz).

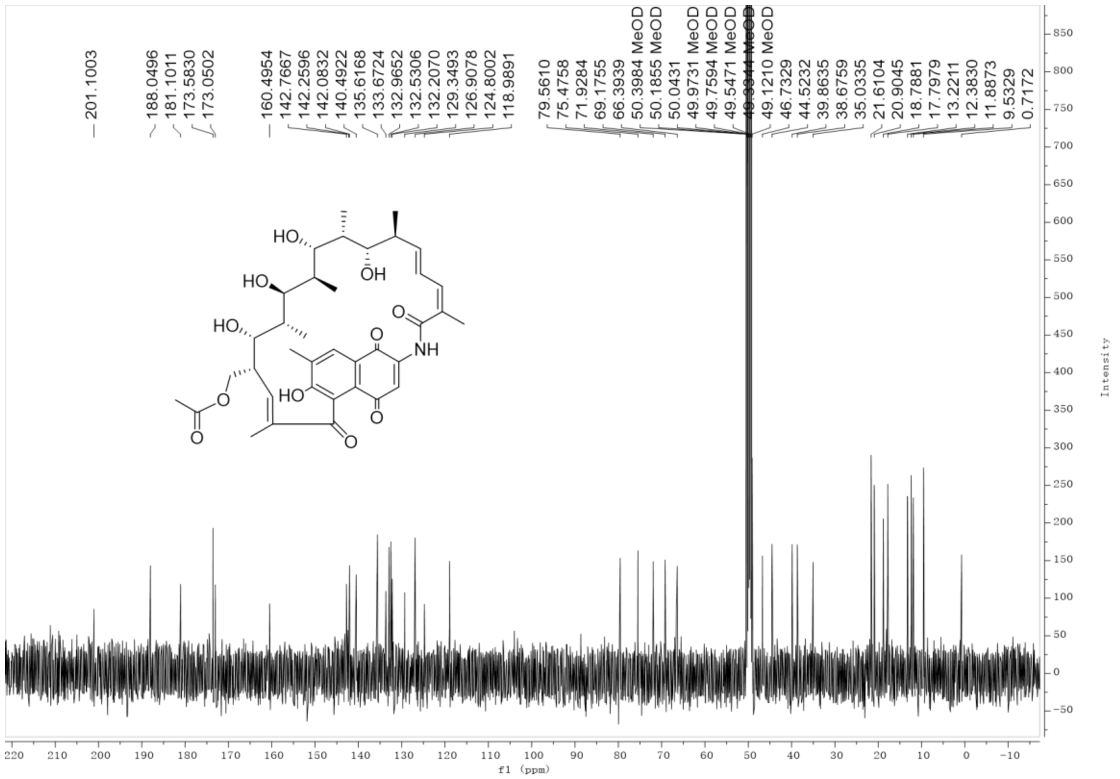

Figure S38. ^{13}C NMR spectrum of **5** in CD_3OD (100 MHz).

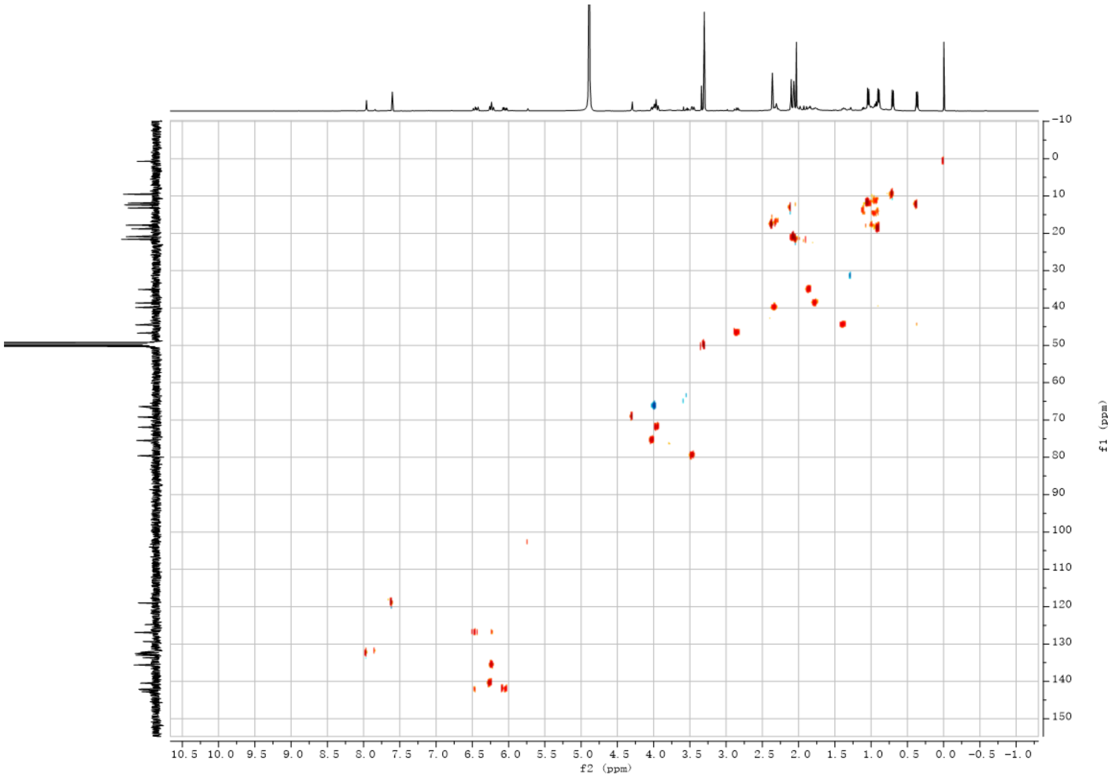

Figure S39. HSQC spectrum of **5** in CD_3OD.

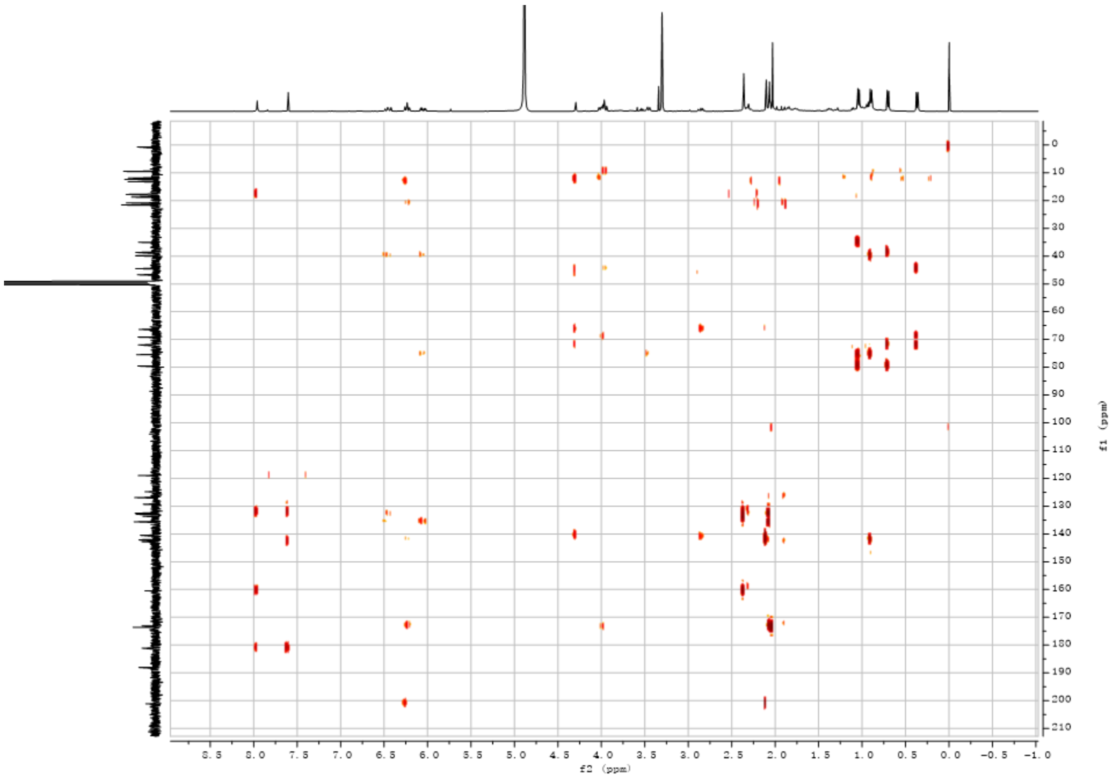

Figure S40. HMBC spectrum of **5** in CD_3OD.

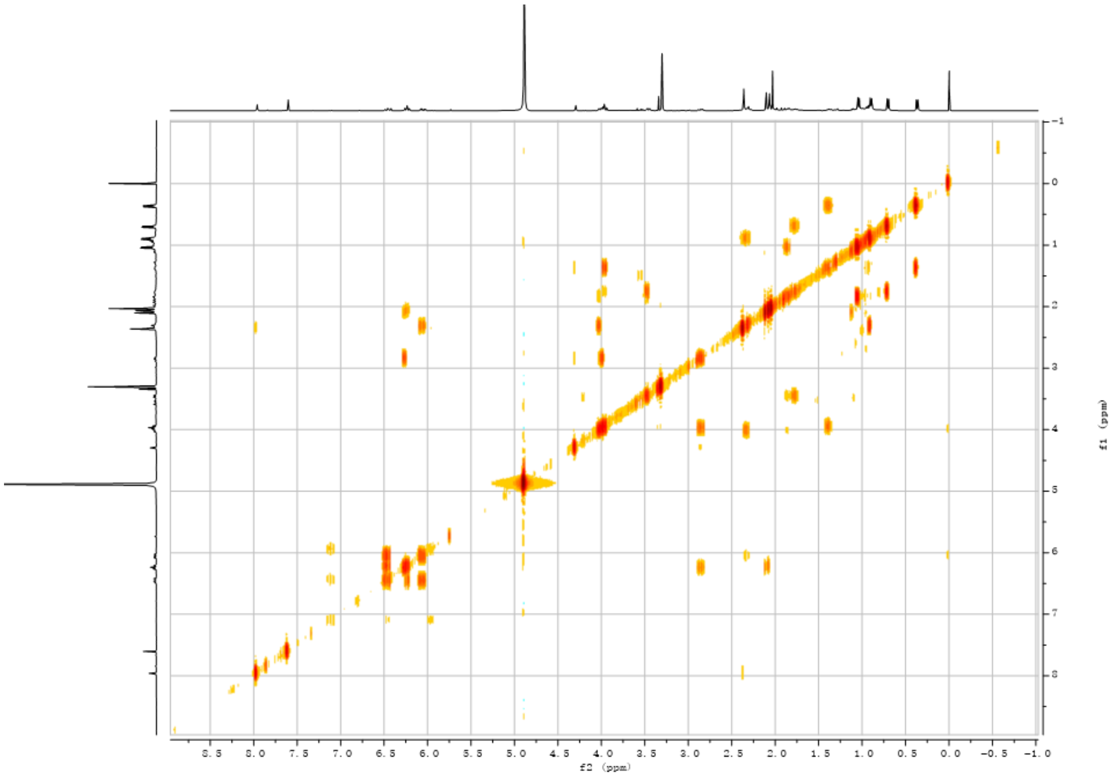

Figure S41. ^1H-^1H COSY spectrum of **5** in CD_3OD.

Figures S42–47. HRESIMS and NMR spectra of **6**

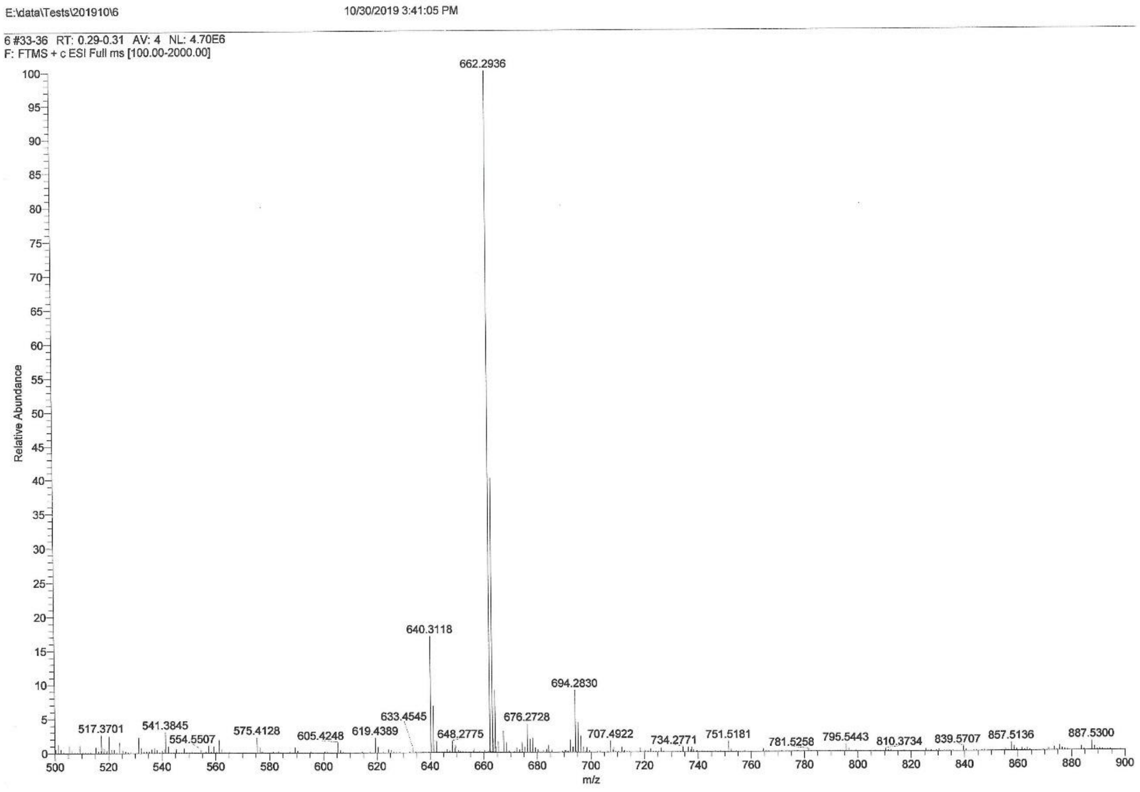

Figure S42. HRESIMS spectrum of **6**.

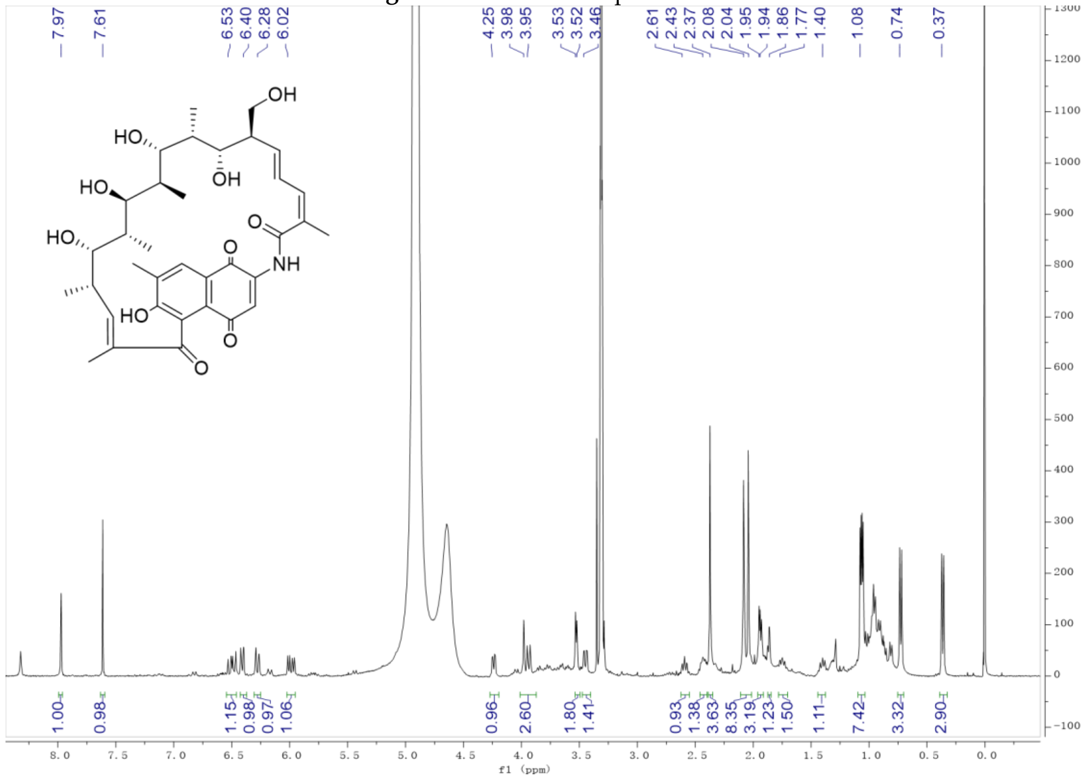

Figure S43. ^{1}H NMR spectrum of **6** in CD_3OD (400 MHz).

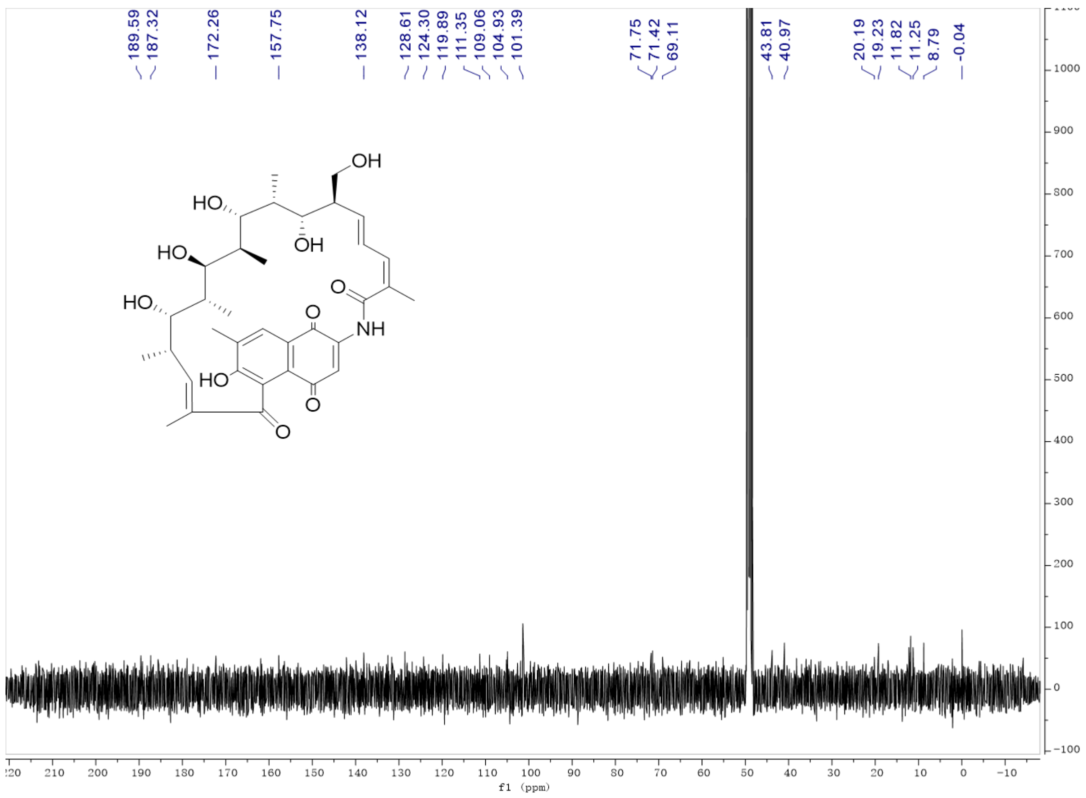

Figure S44. ^{13}C NMR spectrum of **6** in CD_3OD (100 MHz)

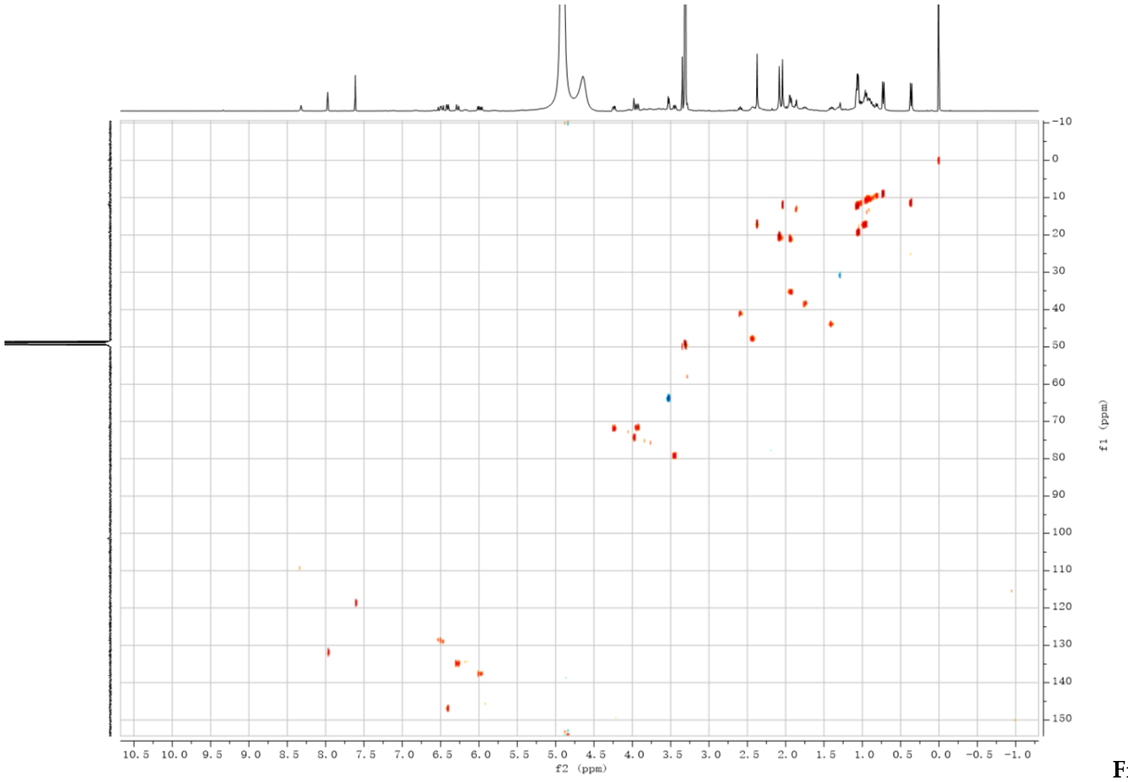

Figure S45. HSQC spectrum of **6** in CD_3OD.

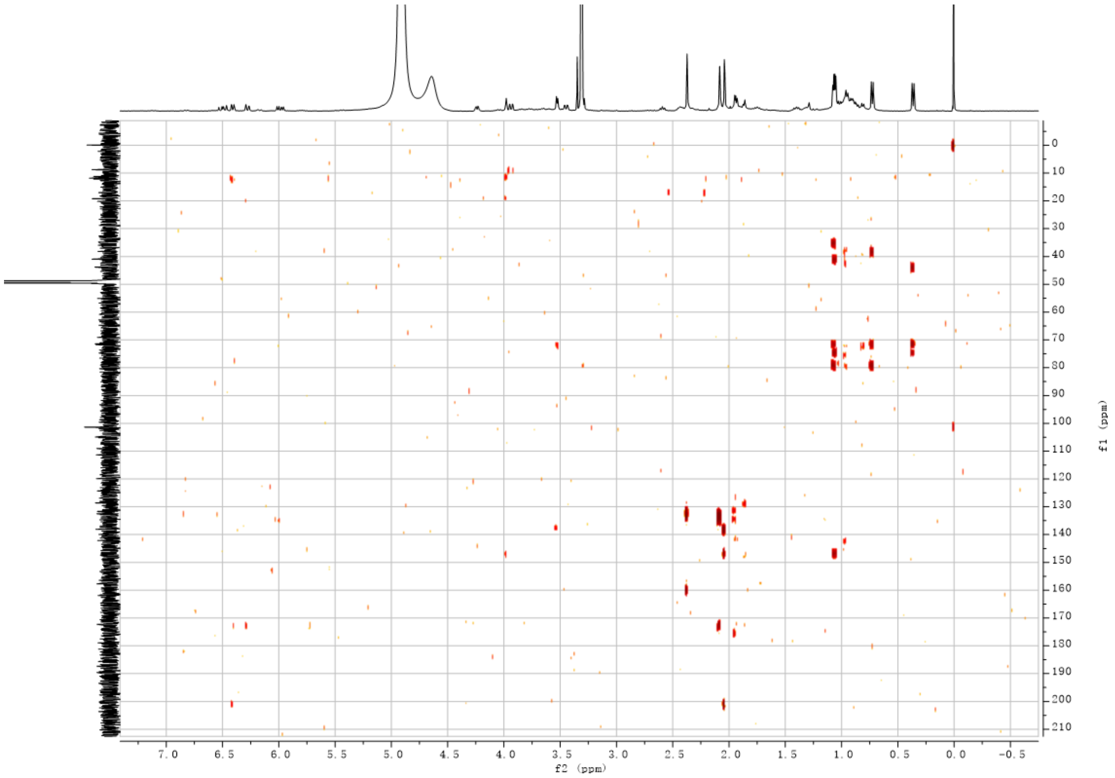

Figure S46. HMBC spectrum of **6** in CD3OD.

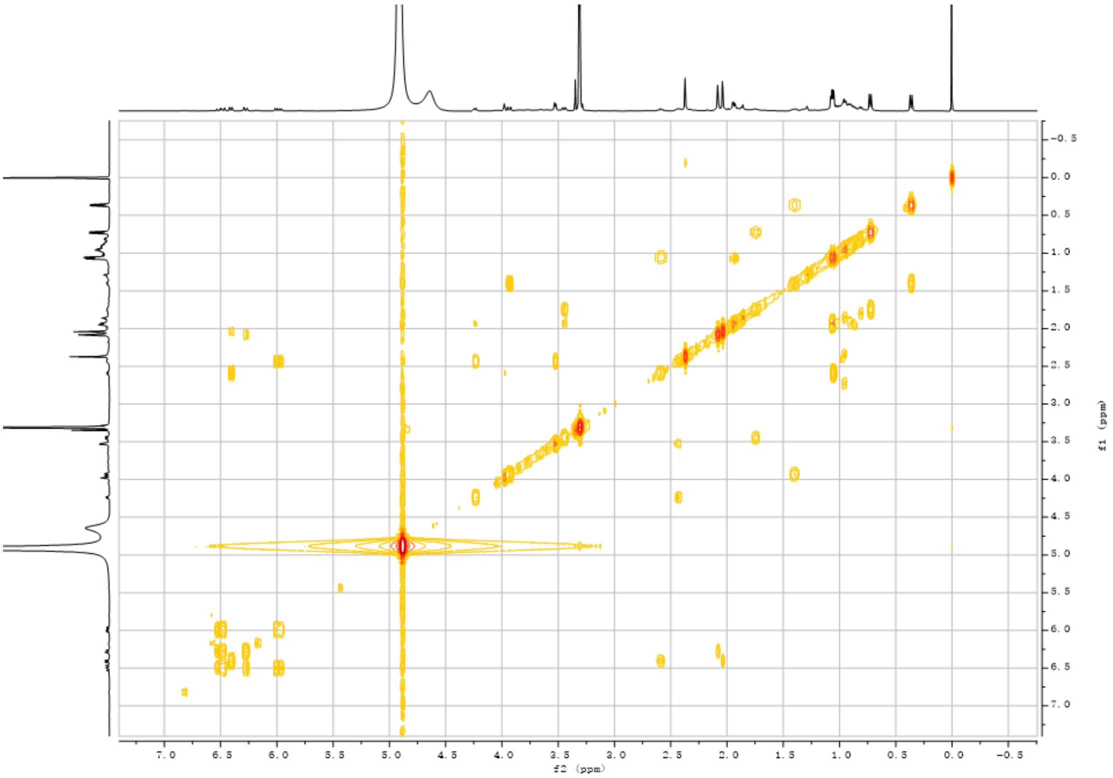

Figure S47. ¹H-¹H COSY spectrum of **6** in CD3OD.

Figures S48–50. HRESIMS spectra of **7**–**9**

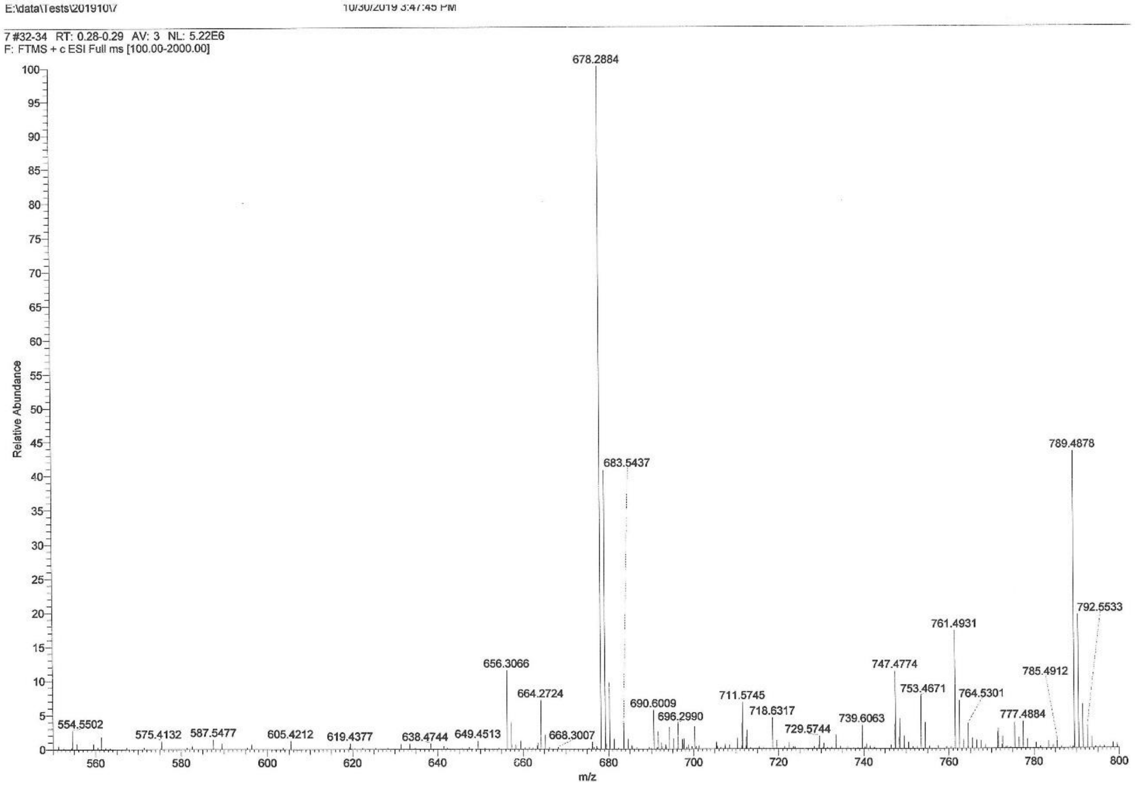

Figure S48. HRESIMS spectrum of **7**.

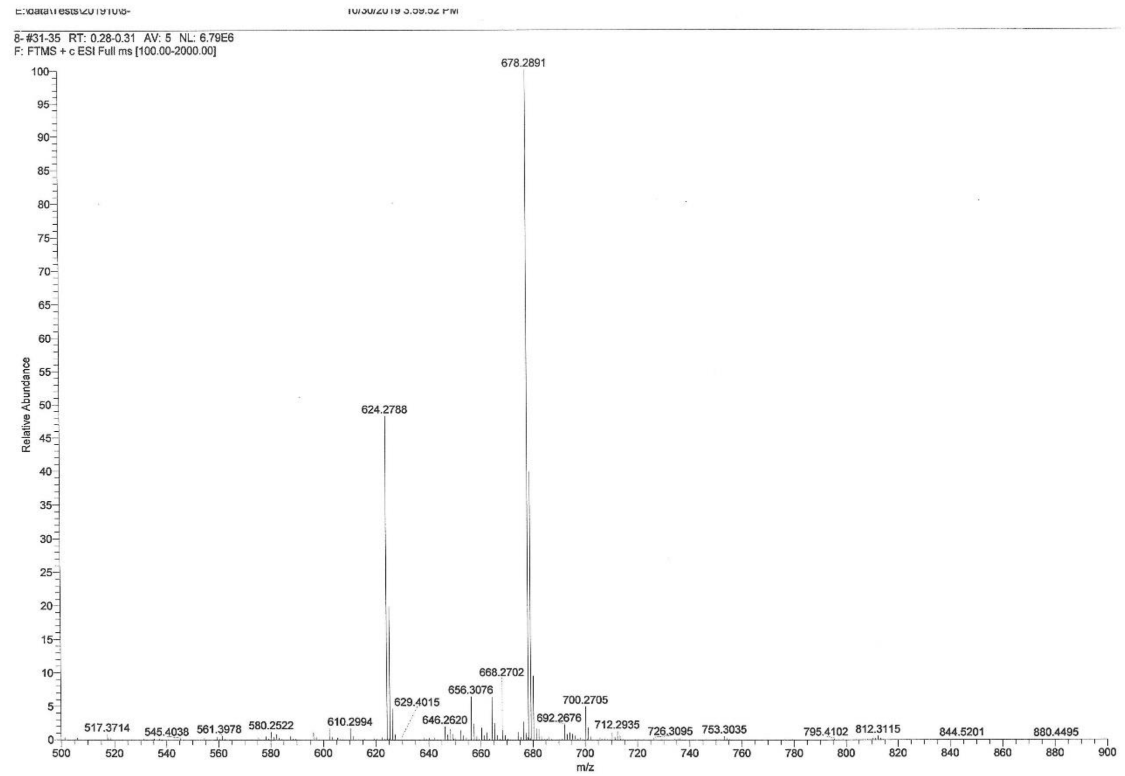

Figure S49. HRESIMS spectrum of **8**

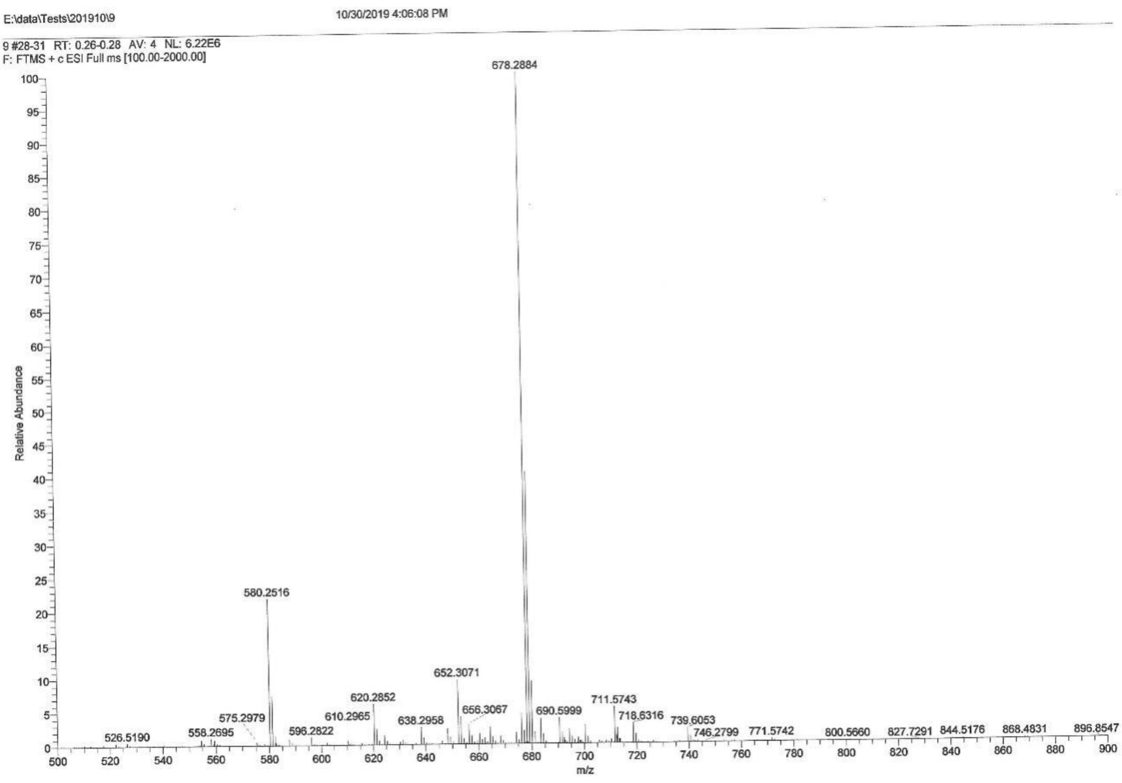

Figure S50. HRESIMS spectrum of **9**.

Figures S51–55. NMR spectra of **7**

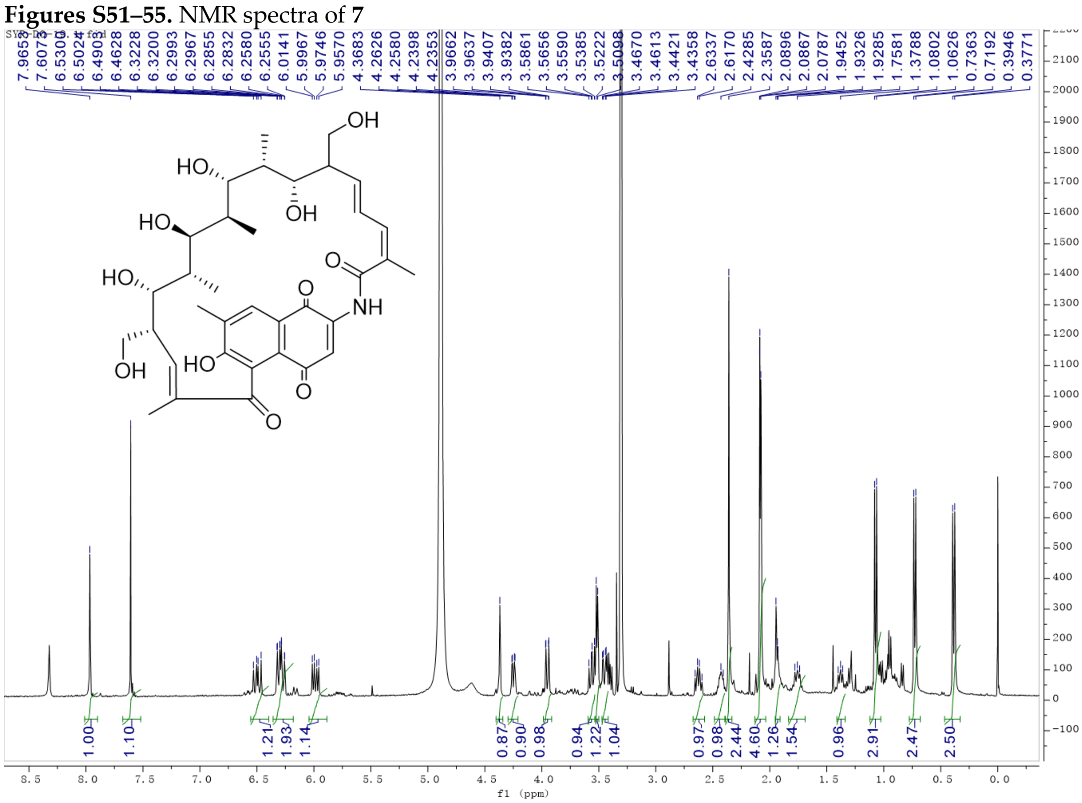

Figure S51. ^{1}H NMR spectrum of **7** in CD_3OD (400 MHz).

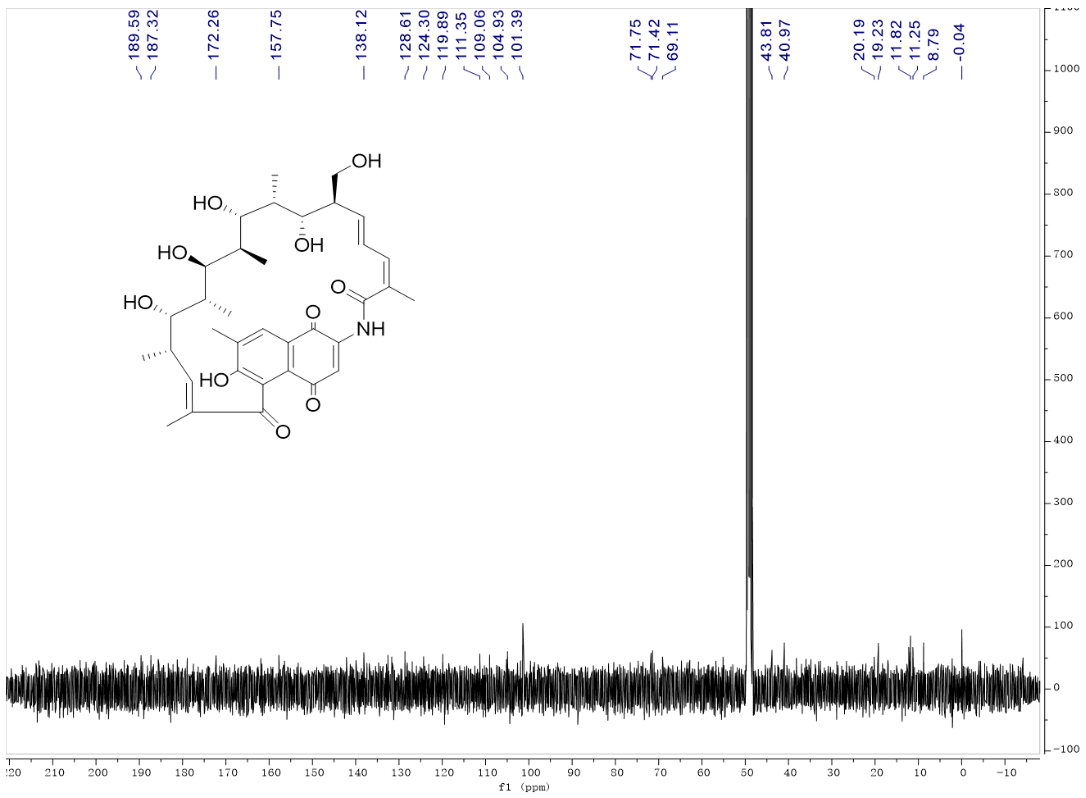

Figure S52. ^{13}C NMR spectrum of **7** in CD_3OD (100 MHz).

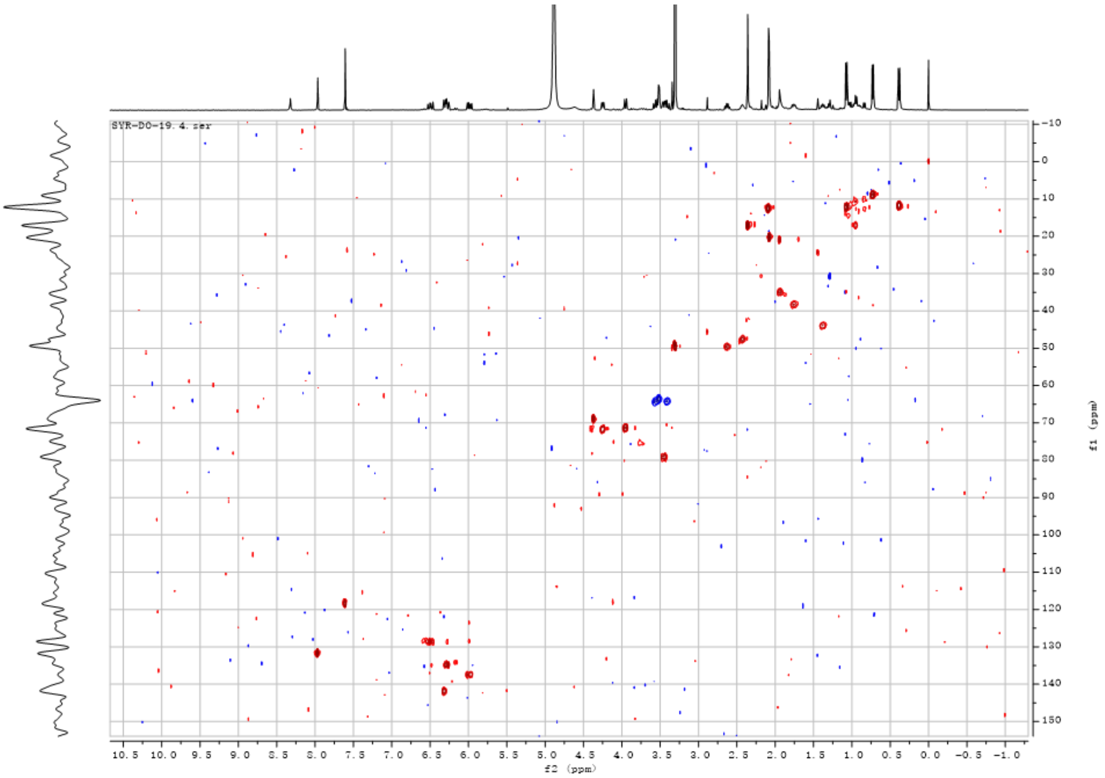

Figure S53. HSQC spectrum of **7** in CD_3OD.

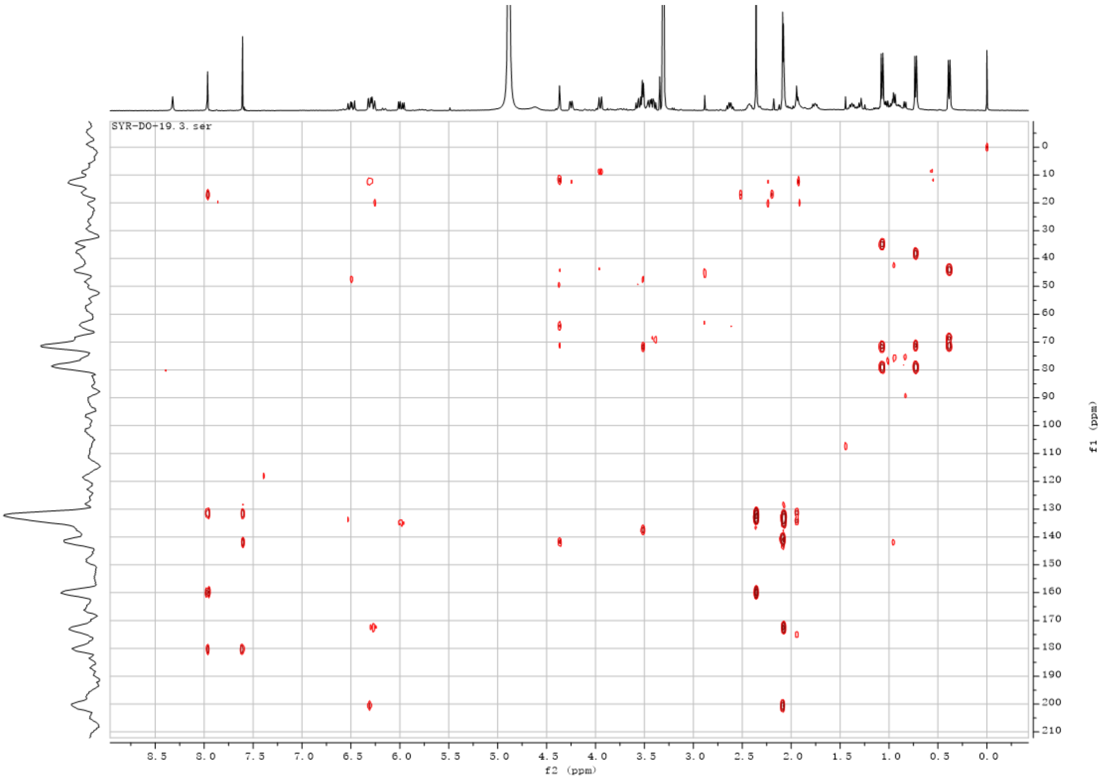

Figure S54. HMBC spectrum of **7** in CD_3OD.

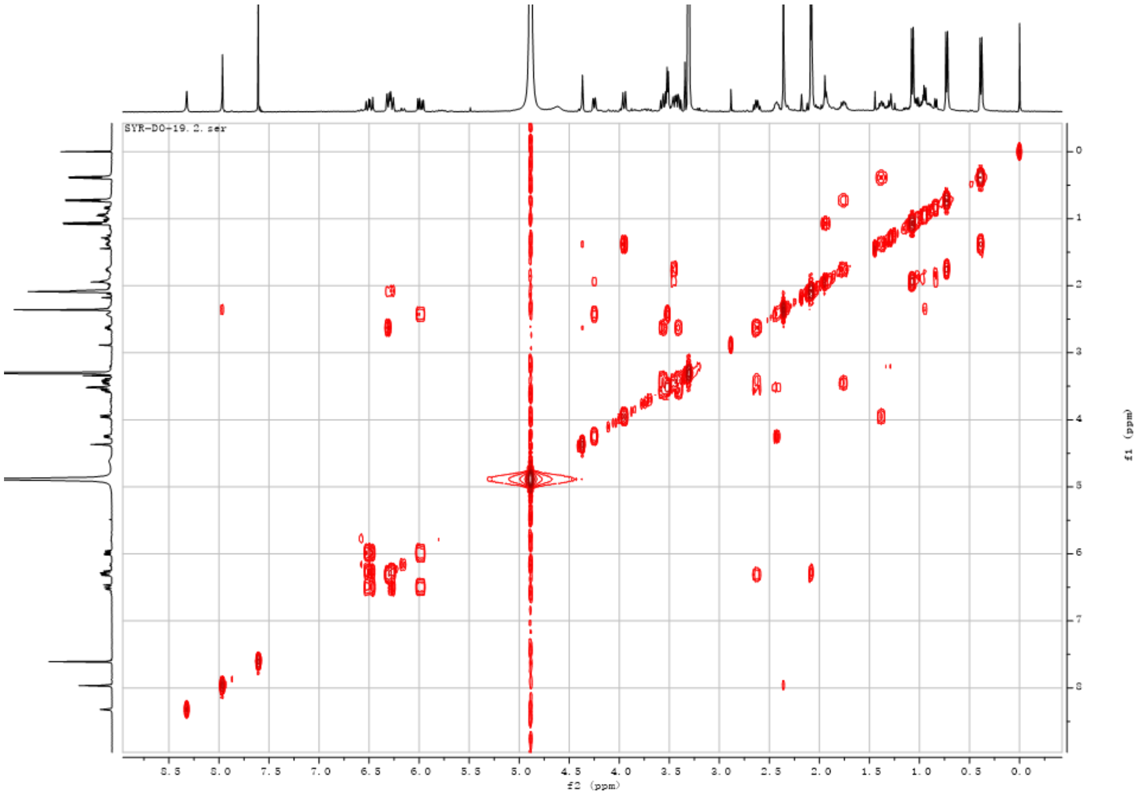

Figure S55. ^1H-^1H COSY spectrum of **7** in CD_3OD.

Figures S56–60. NMR spectra of **8**

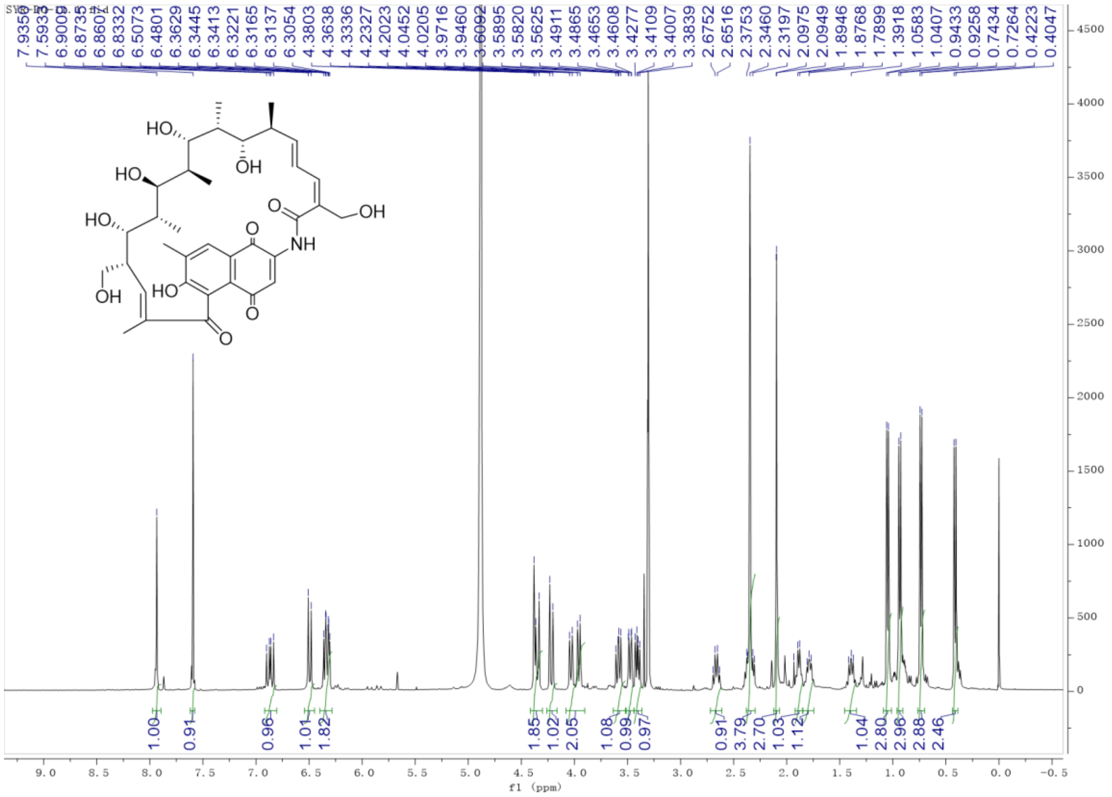

Figure S56. ^{1}H NMR spectrum of **8** in CD_3OD (400 MHz).

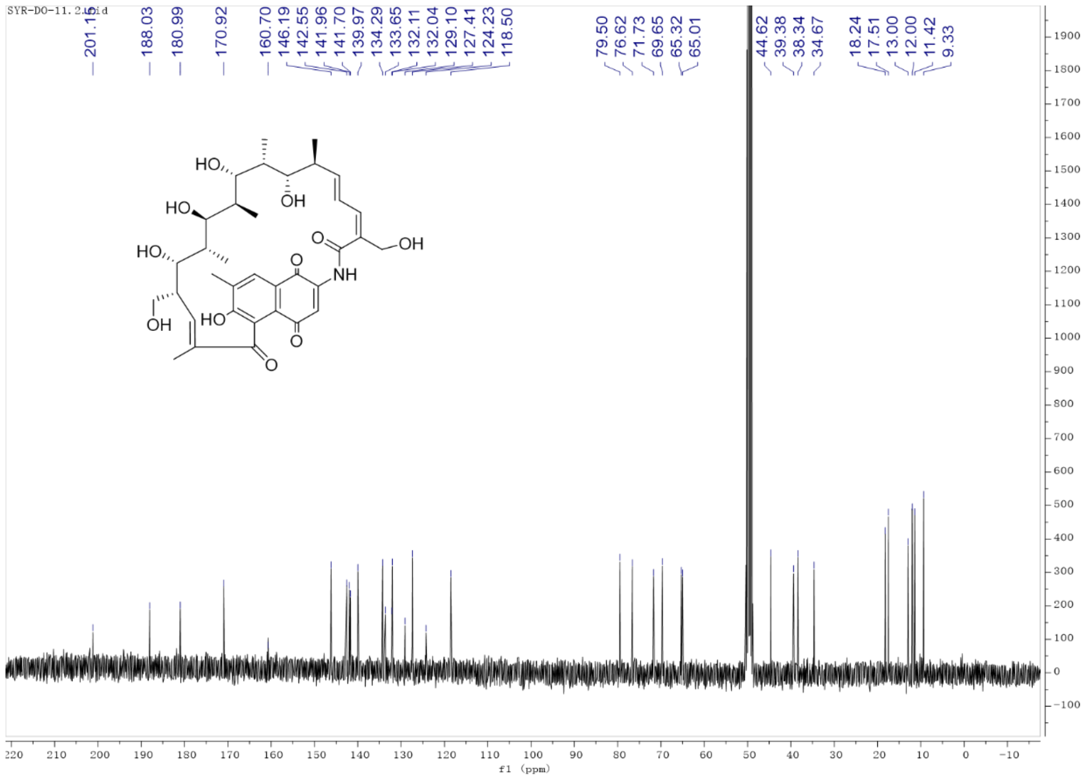

Figure S57. ^{13}C NMR spectrum of **8** in CD_3OD (100 MHz).

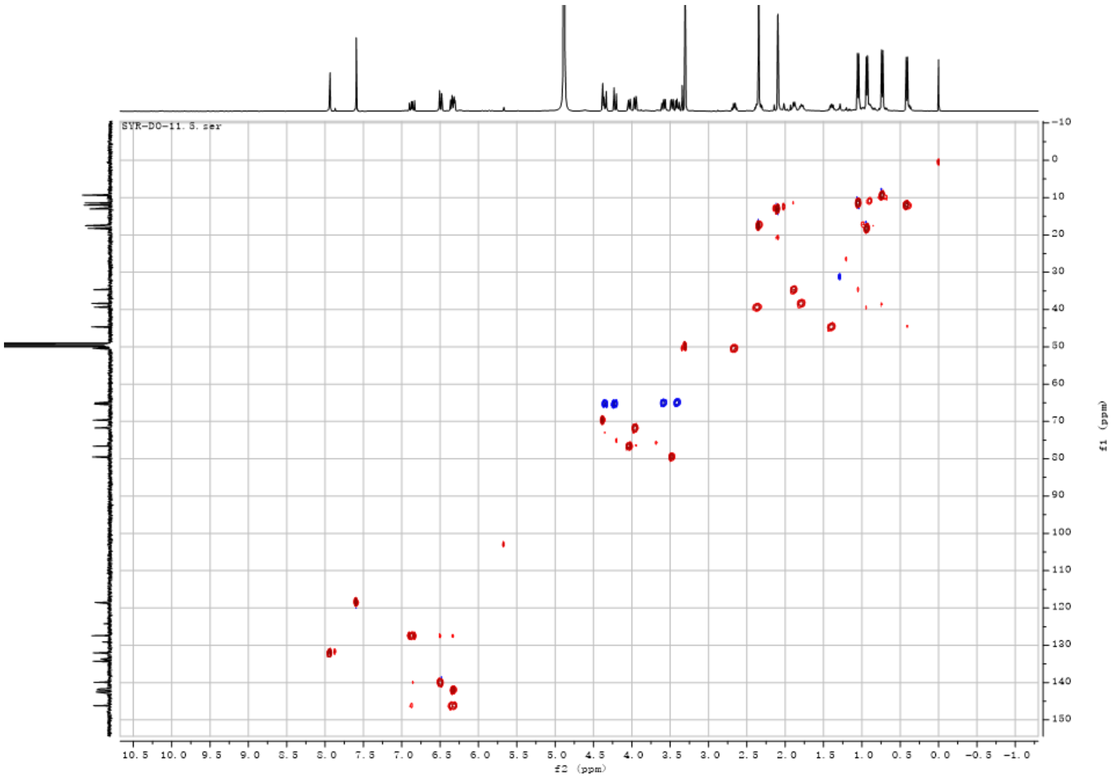

Figure S58. HSQC spectrum of **8** in CD_3OD.

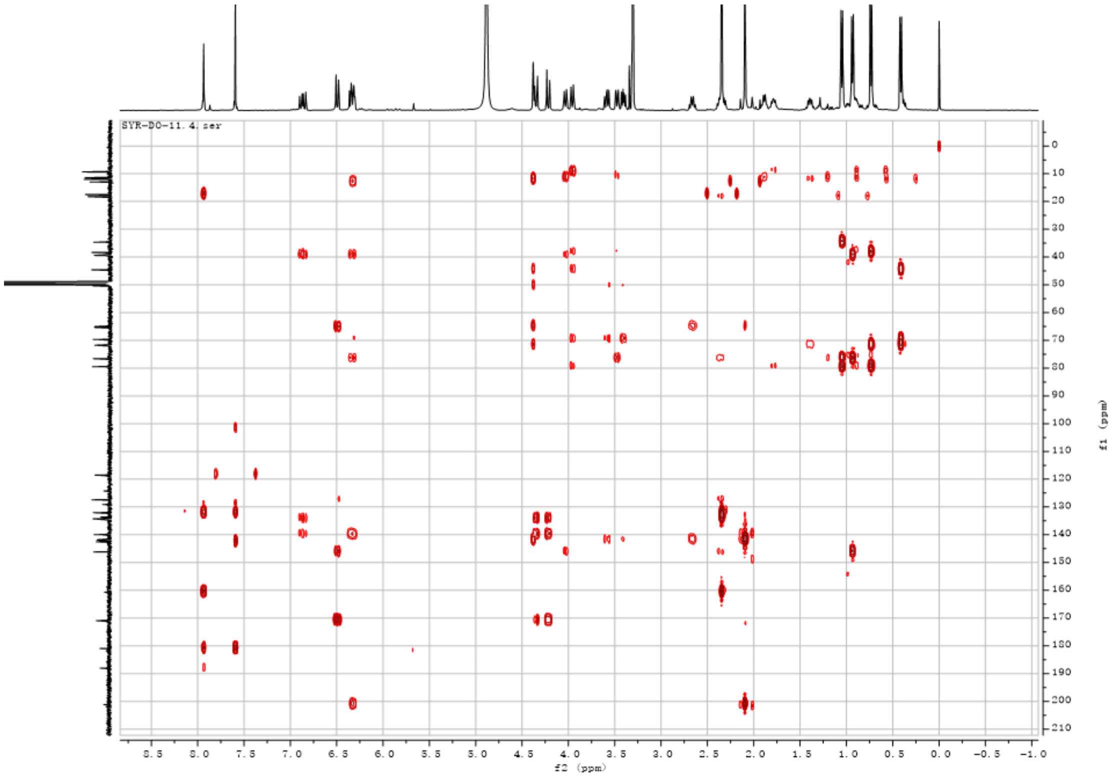

Figure S59. HMBC spectrum of **8** in CD_3OD.

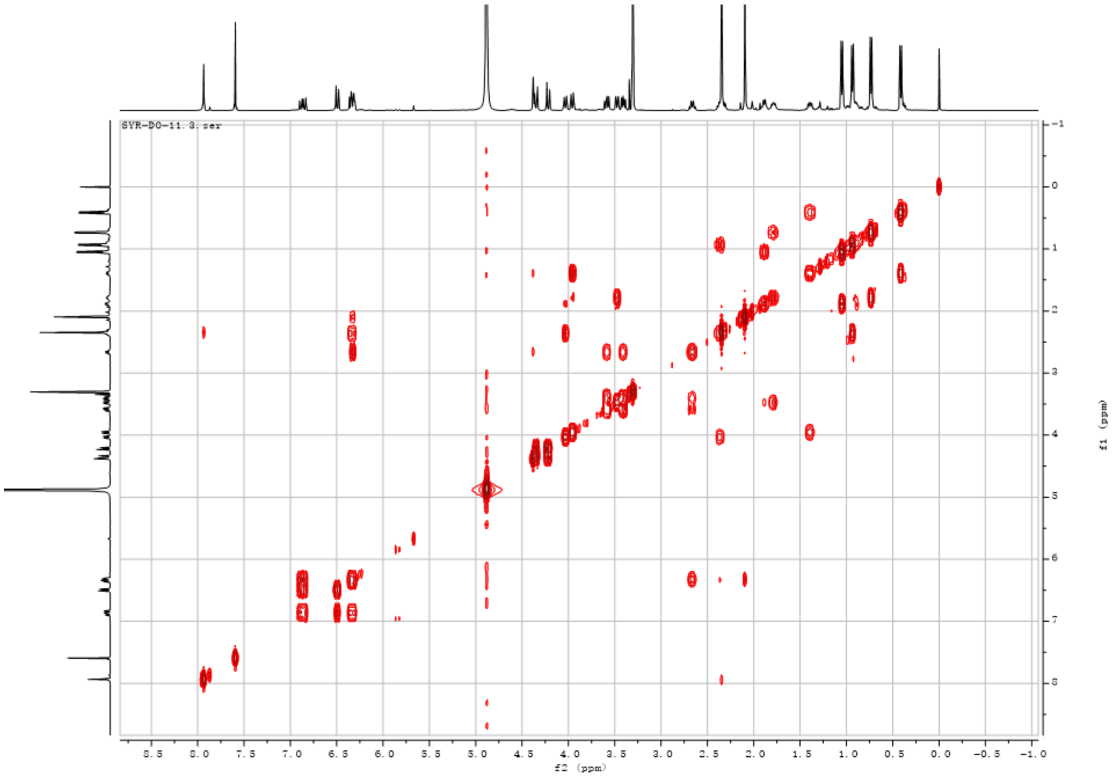

Figure S60. ¹H-¹H COSY spectrum of **8** in CD3OD.

Figures S61–65. NMR spectra of **9**

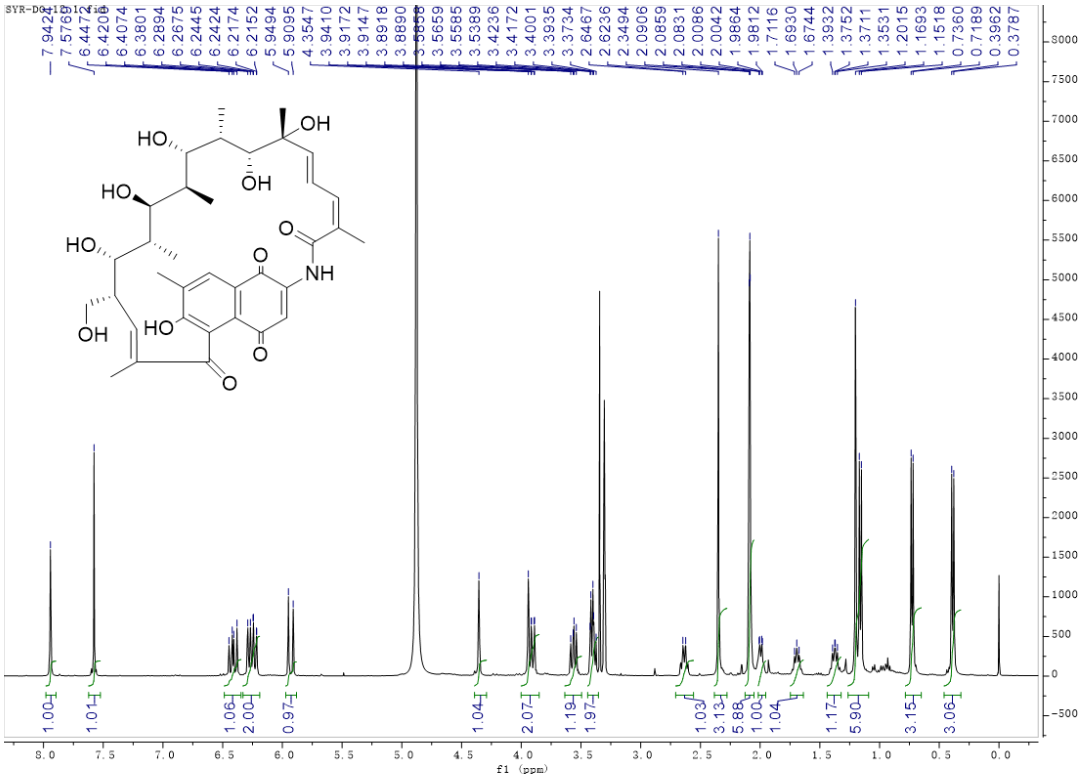

Figure S61. ¹H NMR spectrum of **9** in CD3OD (400 MHz).

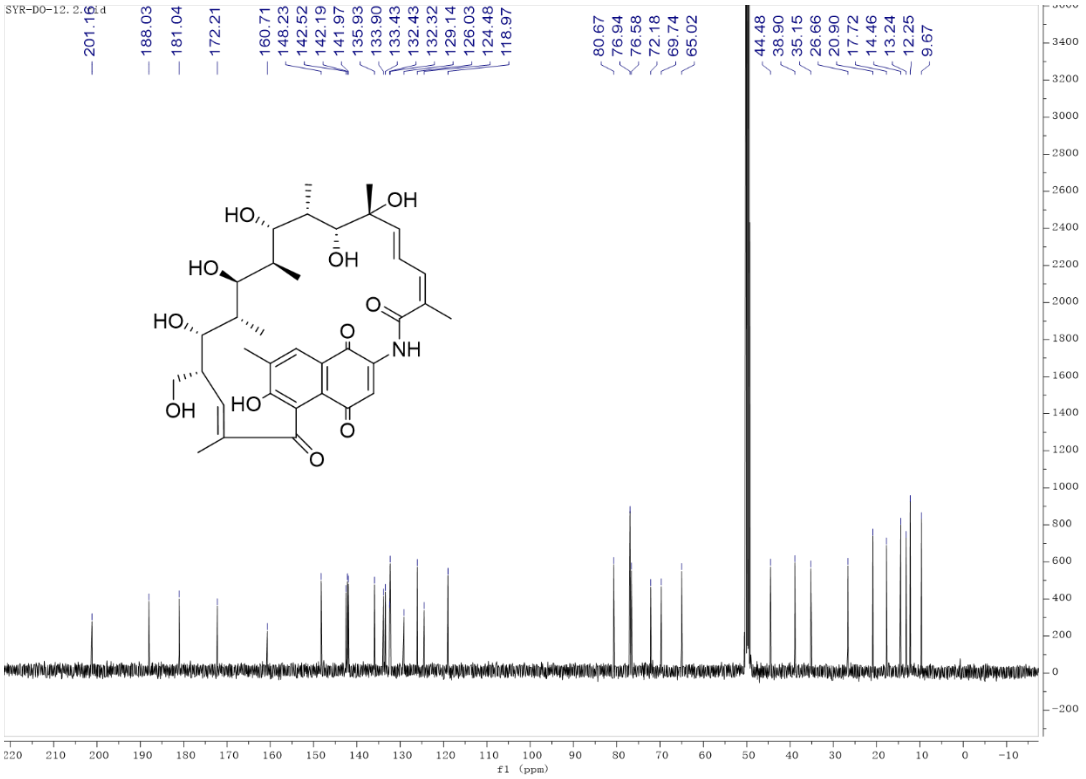

Figure S62. ^{13}C NMR spectrum of **9** in CD_3OD (100 MHz).

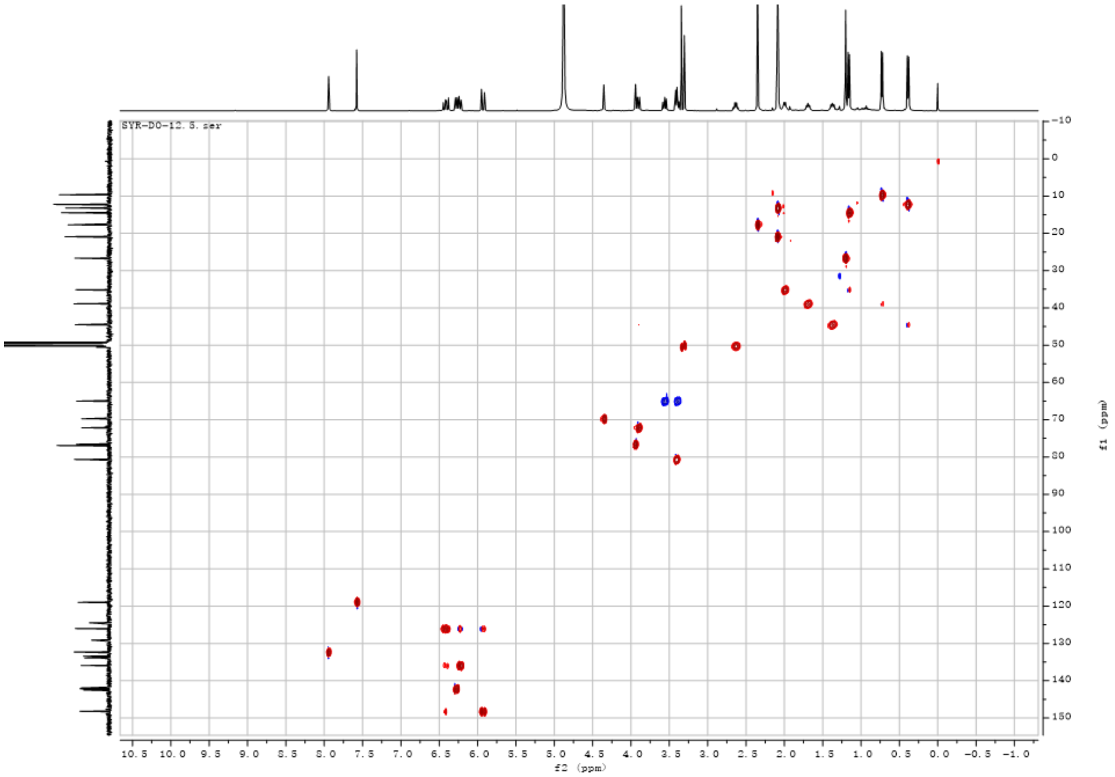

Figure S63. HSQC spectrum of **9** in CD_3OD.

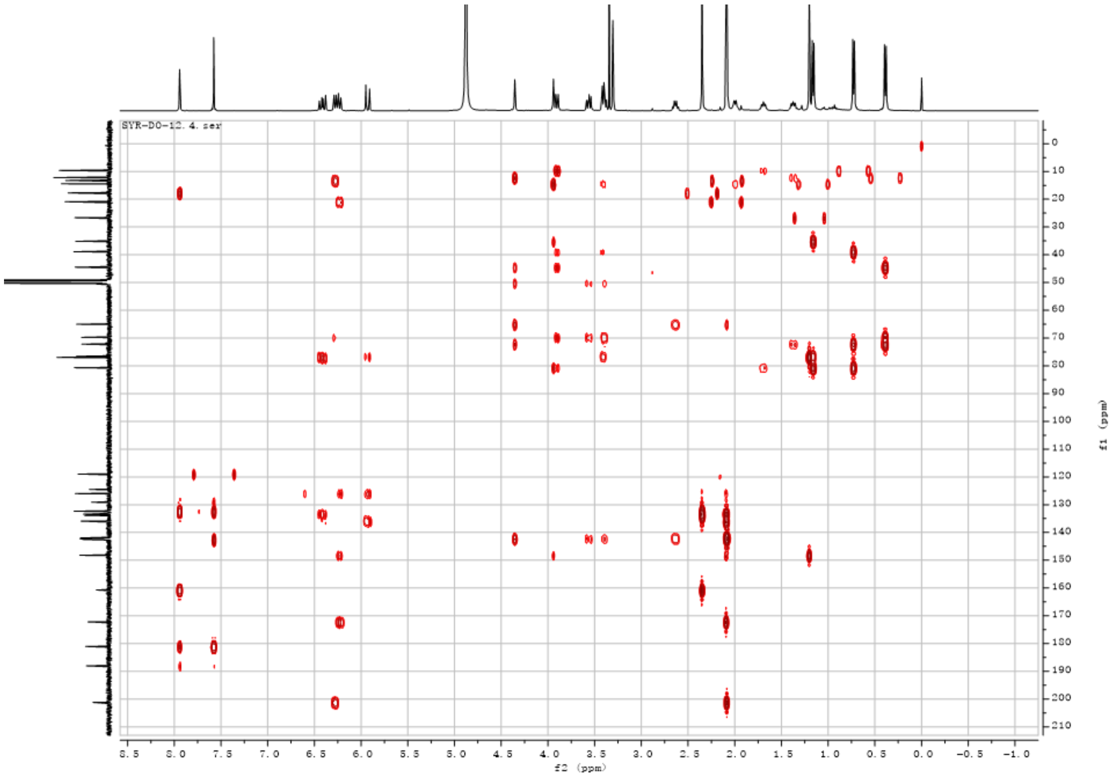

Figure S64. HMBC spectrum of **9** in CD_3OD.

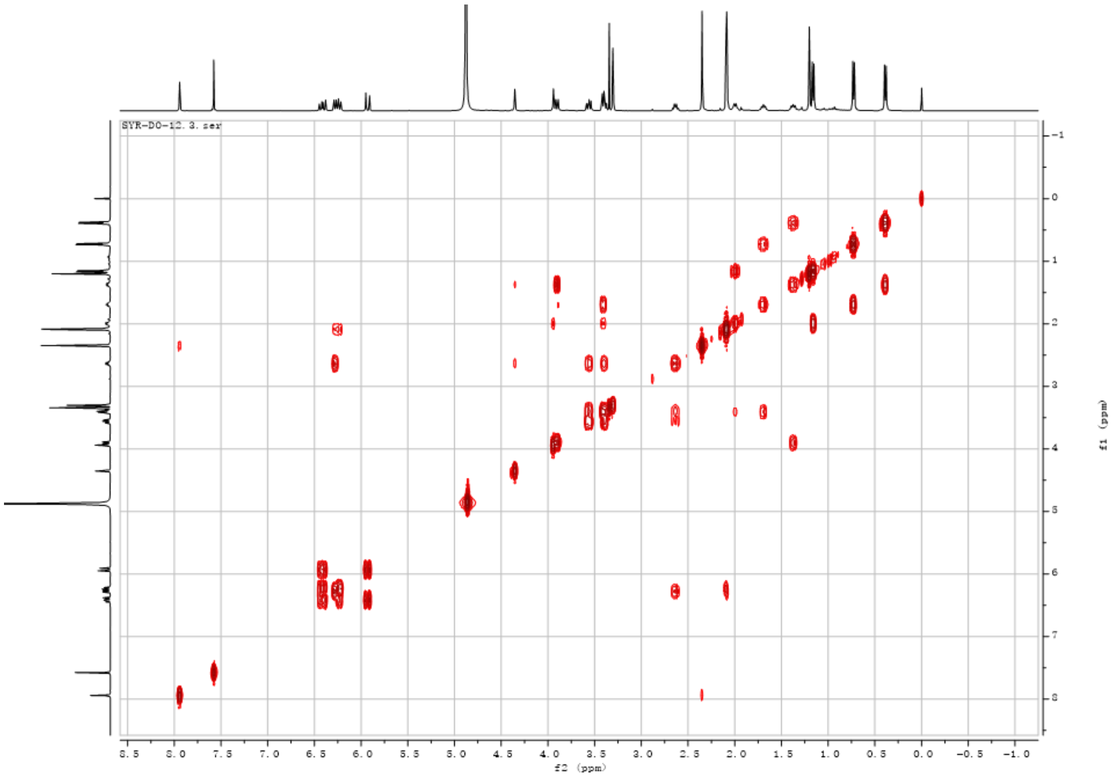

Figure S65. ^1H-^1H COSY spectrum of **9** in CD_3OD.

Figures S66 and S67. HRESIMS spectra of **10** and **11**

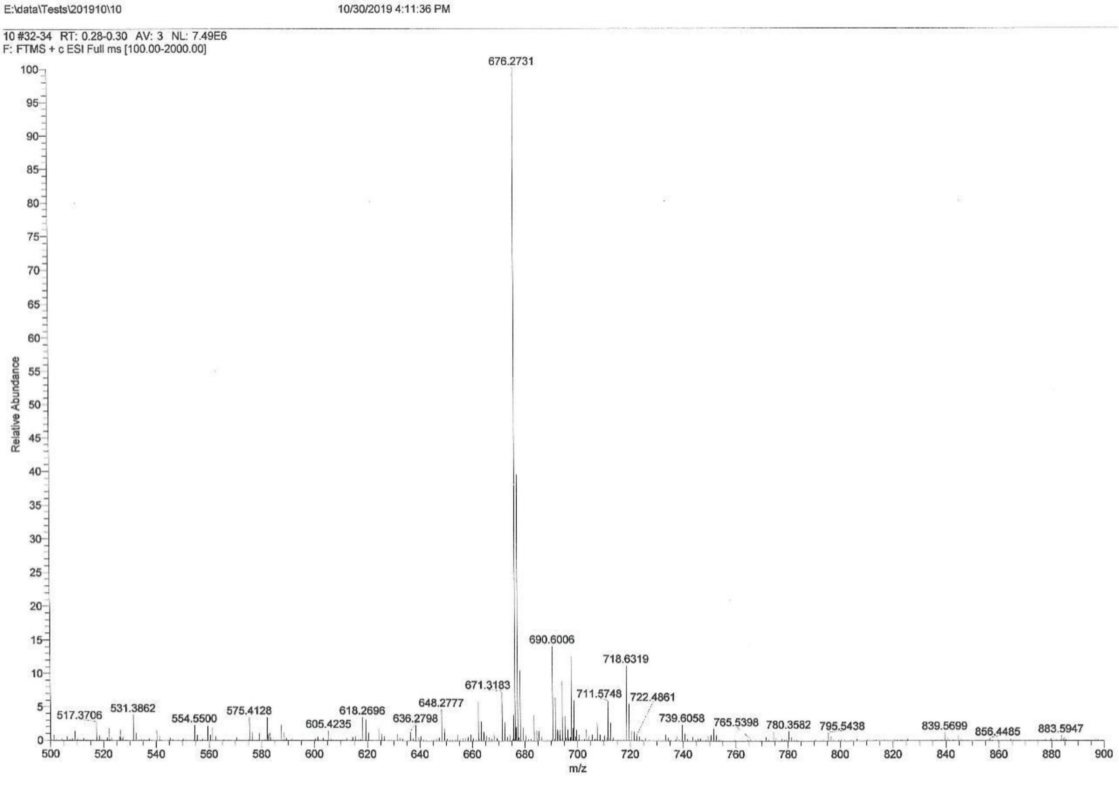

Figure S66. HRESIMS spectrum of **10**.

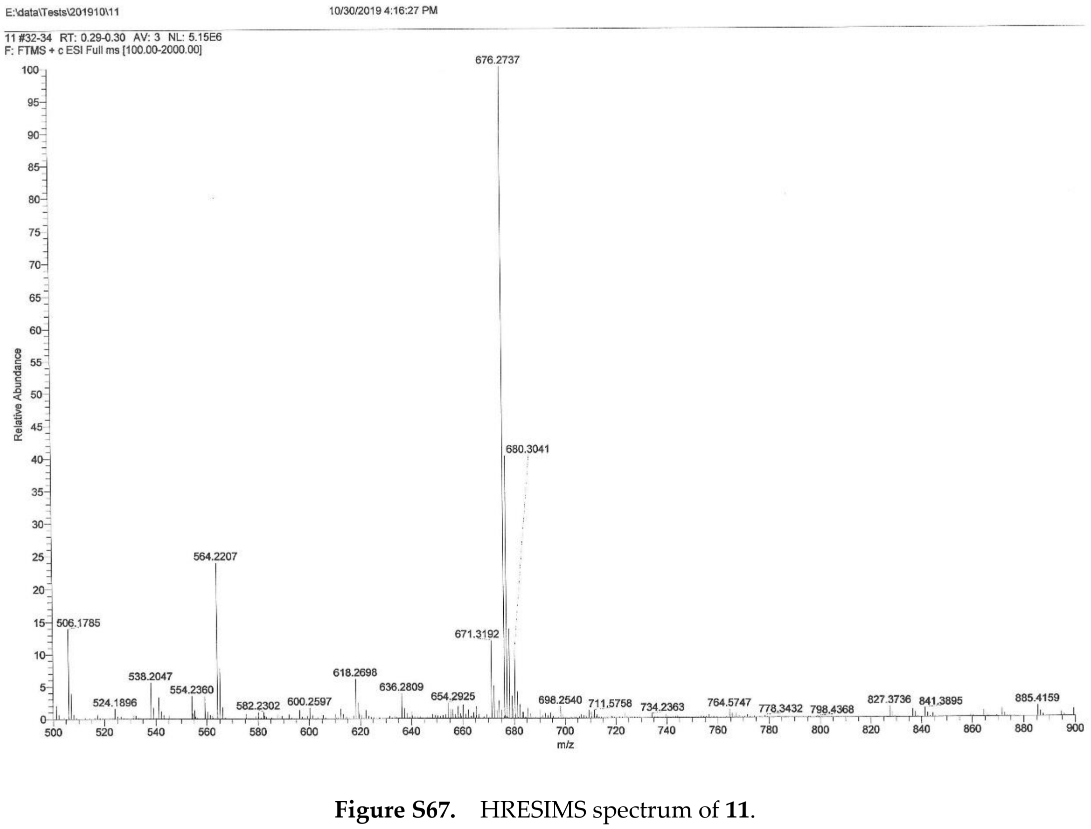

Figure S67. HRESIMS spectrum of **11**.

Figures S68–72. NMR spectra of **10**

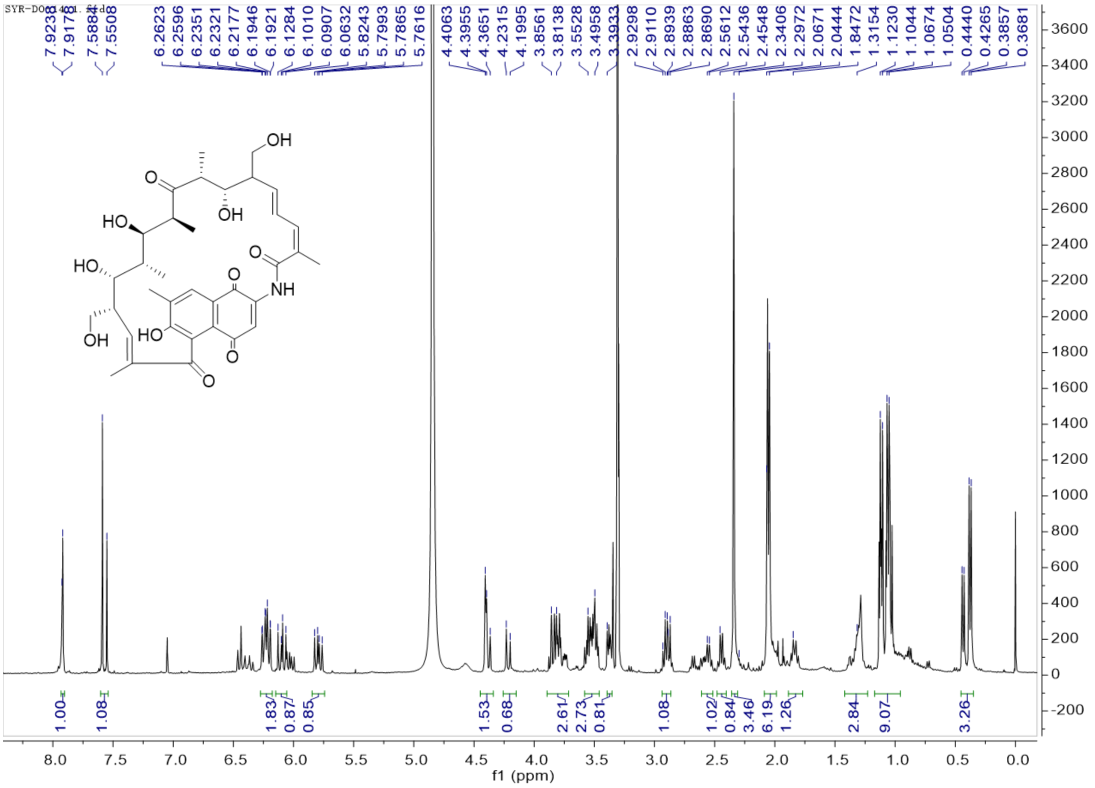

Figure S68. ^{1}H NMR spectrum of **10** in CD_3OD (400 MHz).

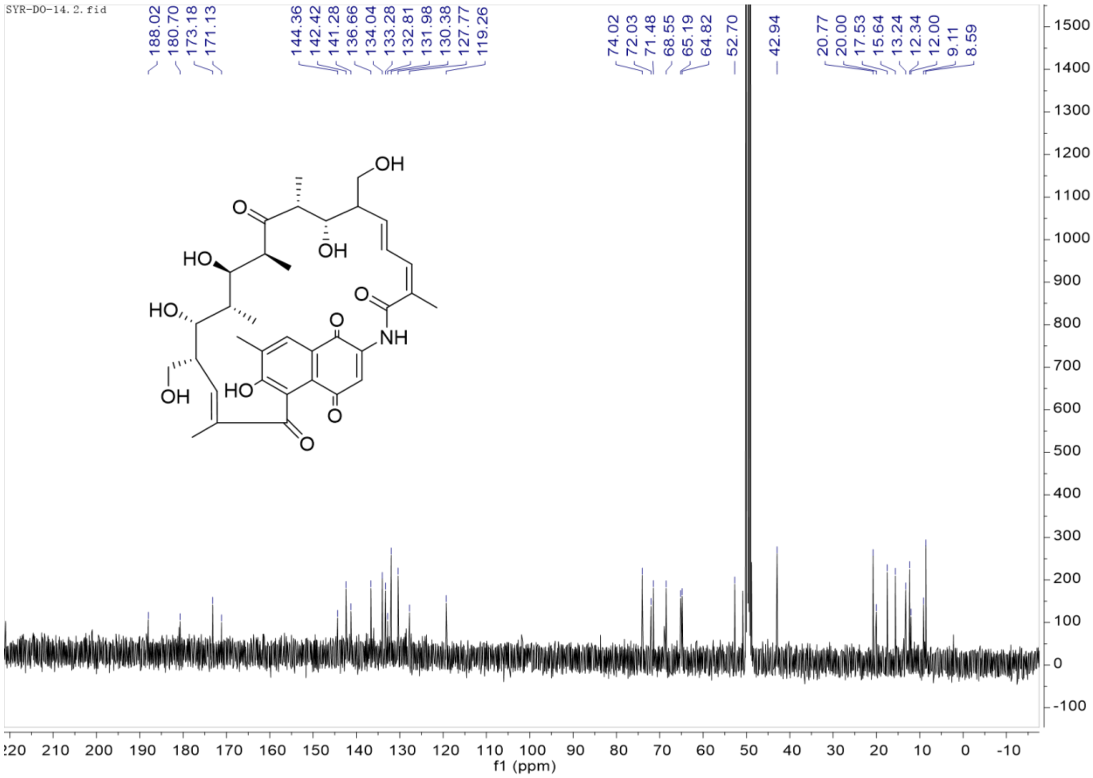

Figure S69. ^{13}C NMR spectrum of **10** in CD_3OD (100 MHz).

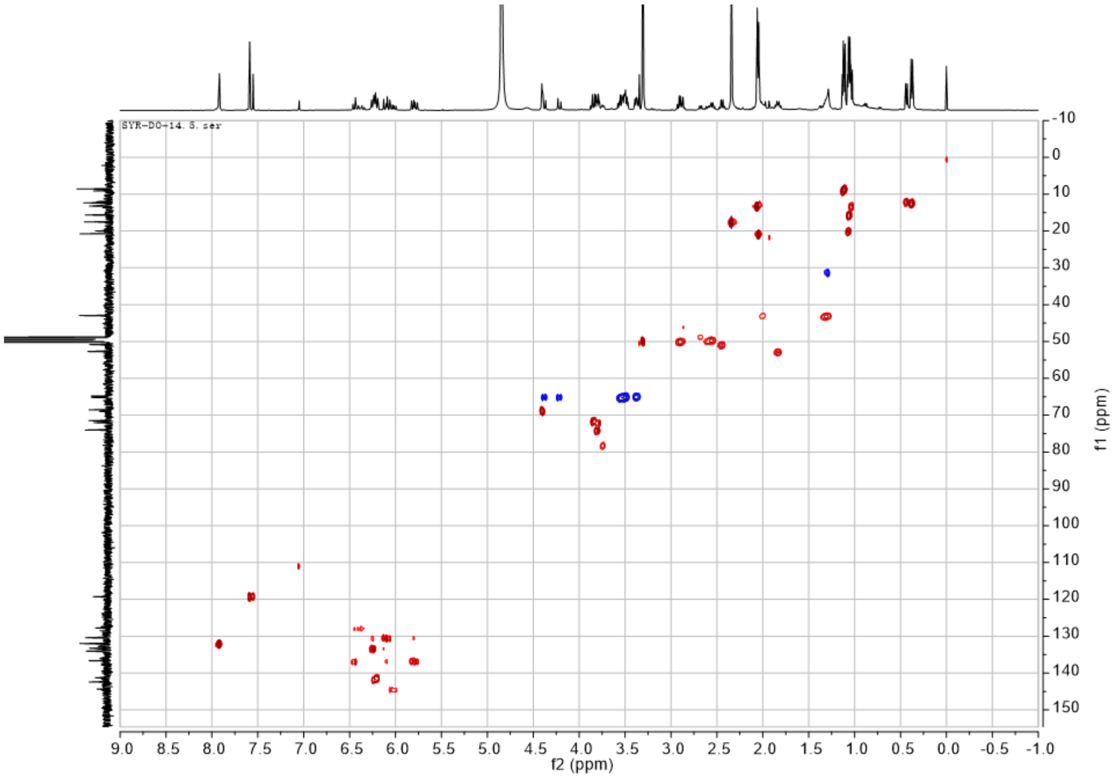

Figure S70. HSQC spectrum of **10** in CD_3OD.

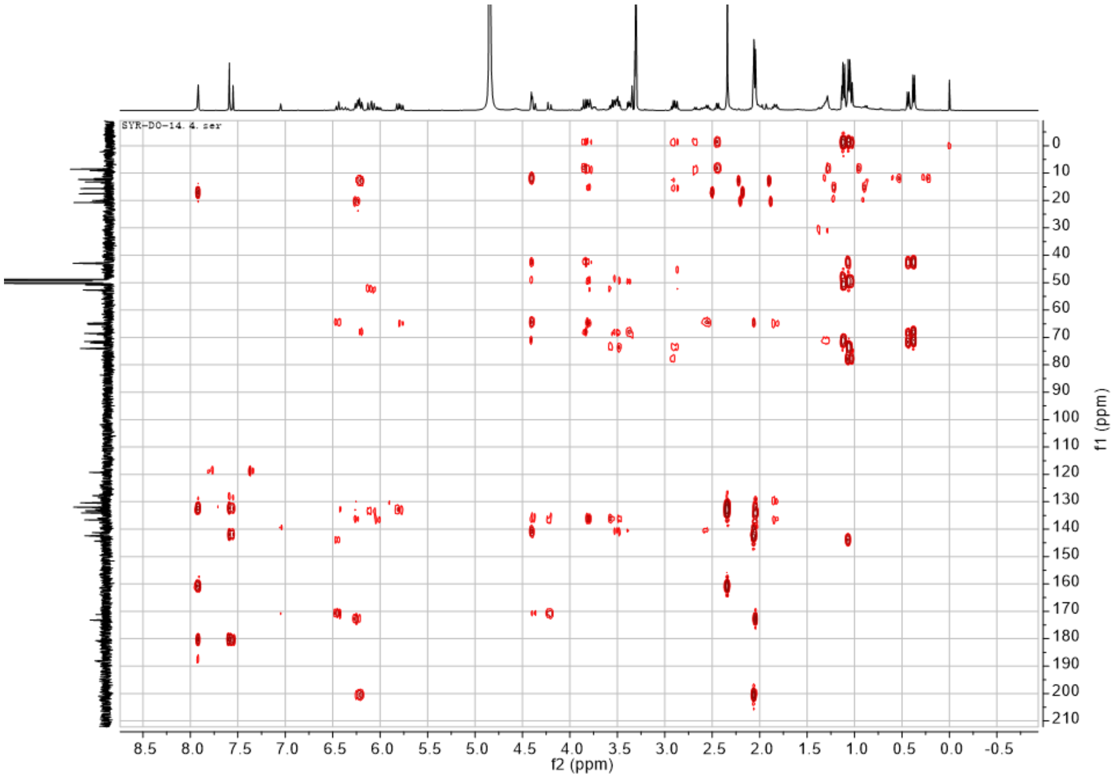

Figure S71. HMBC spectrum of **10** in CD_3OD.

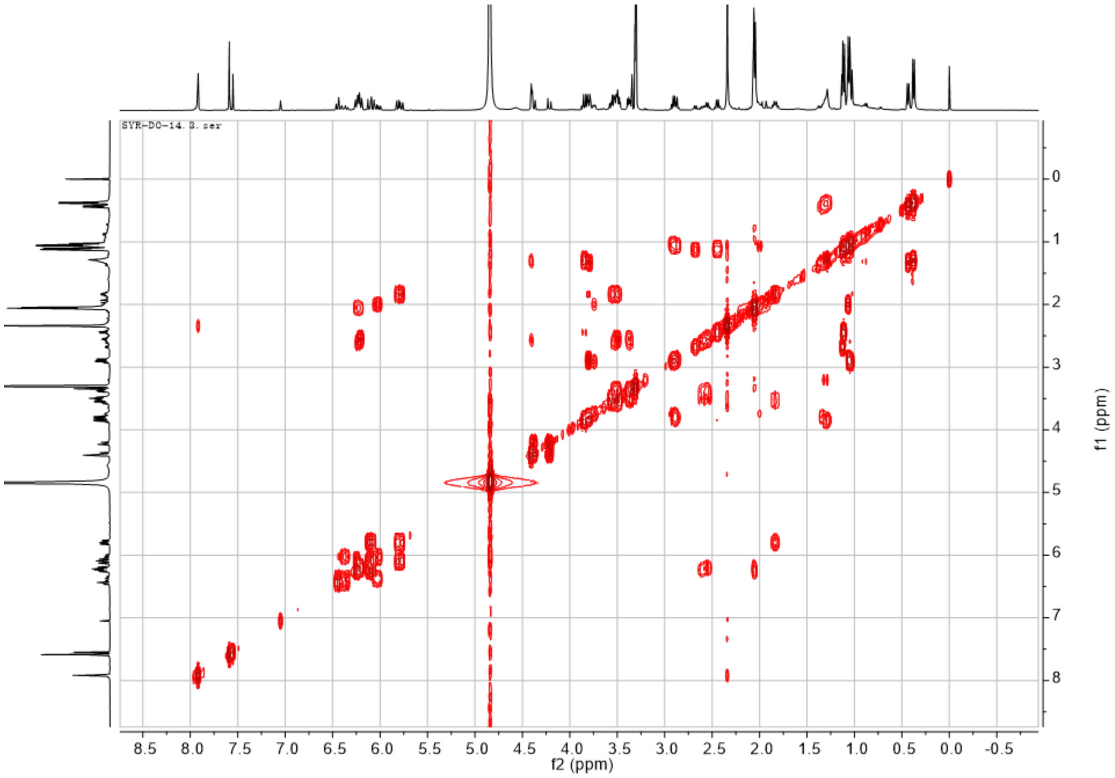

Figure S72. ^1H-^1H COSY spectrum of **10** in CD_3OD.

Figures S73–77. NMR spectra of **11**

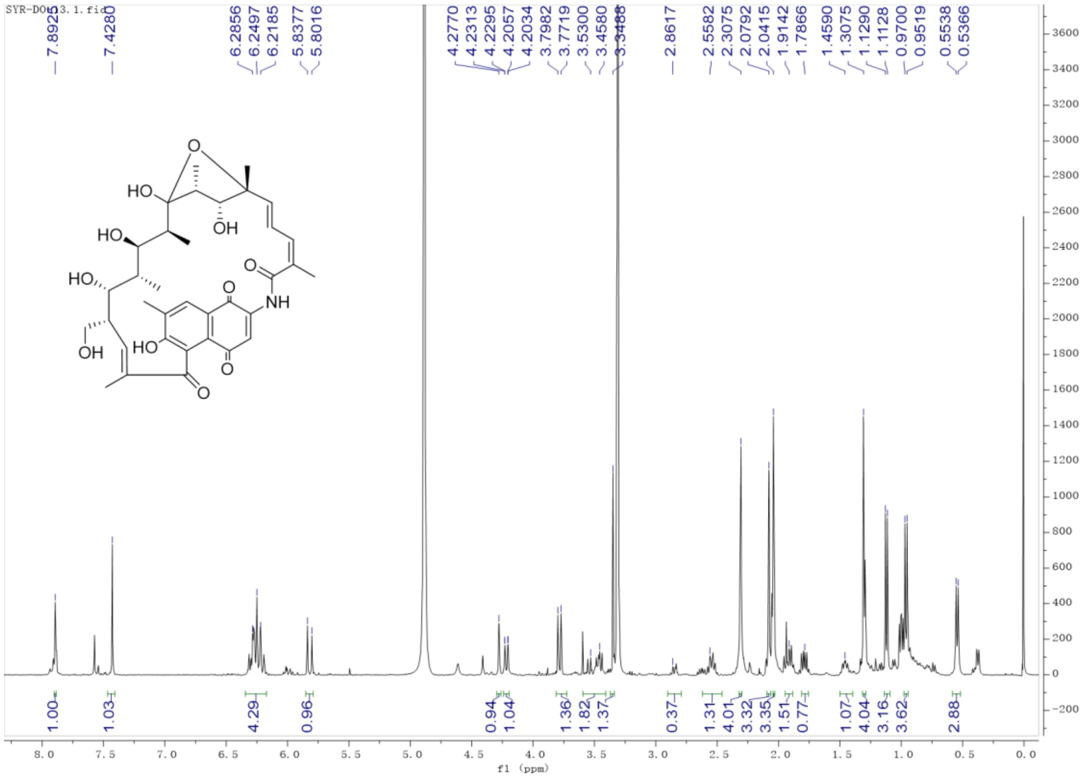

Figure S73. ^1H NMR spectrum of **11** in CD_3OD (400 MHz).

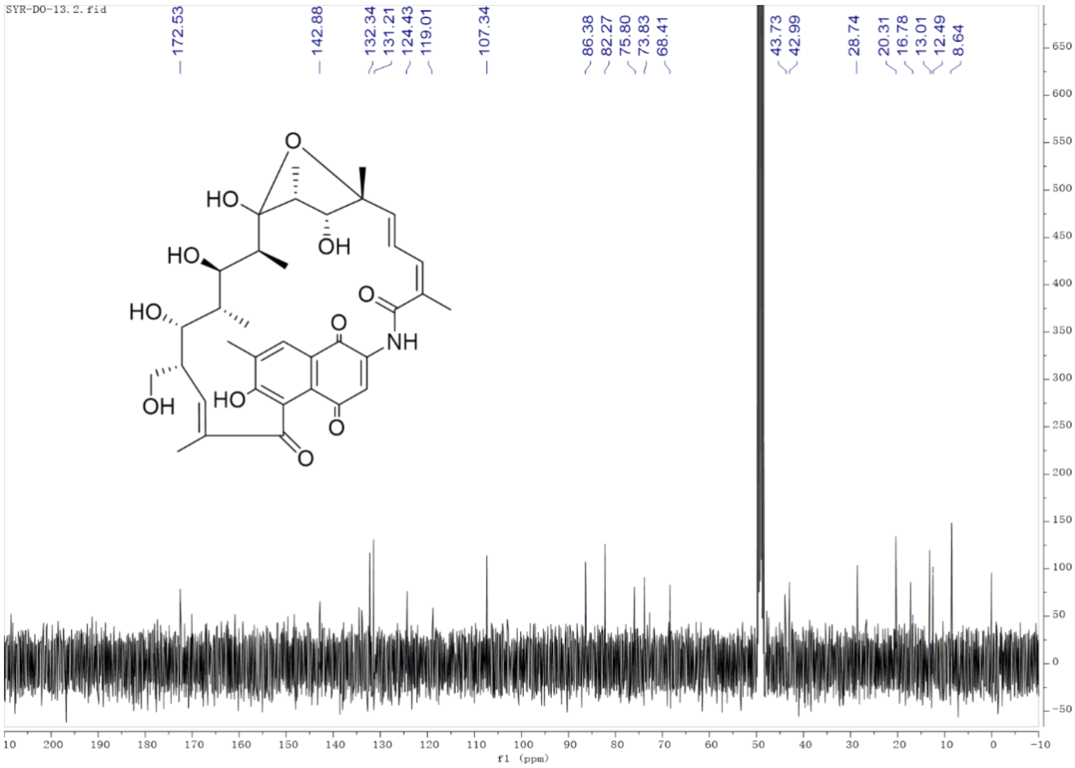

Figure S74. ^{13}C NMR spectrum of **11** in CD_3OD (100 MHz).

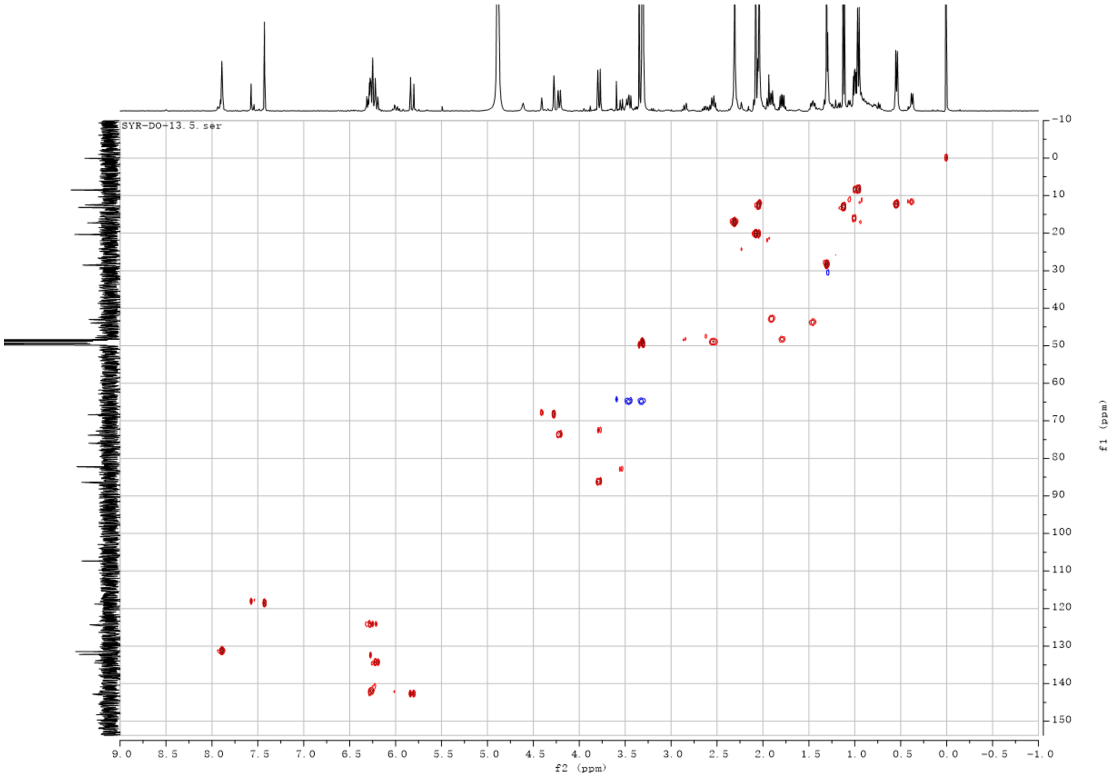

Figure S75. HSQC spectrum of **11** in CD_3OD.

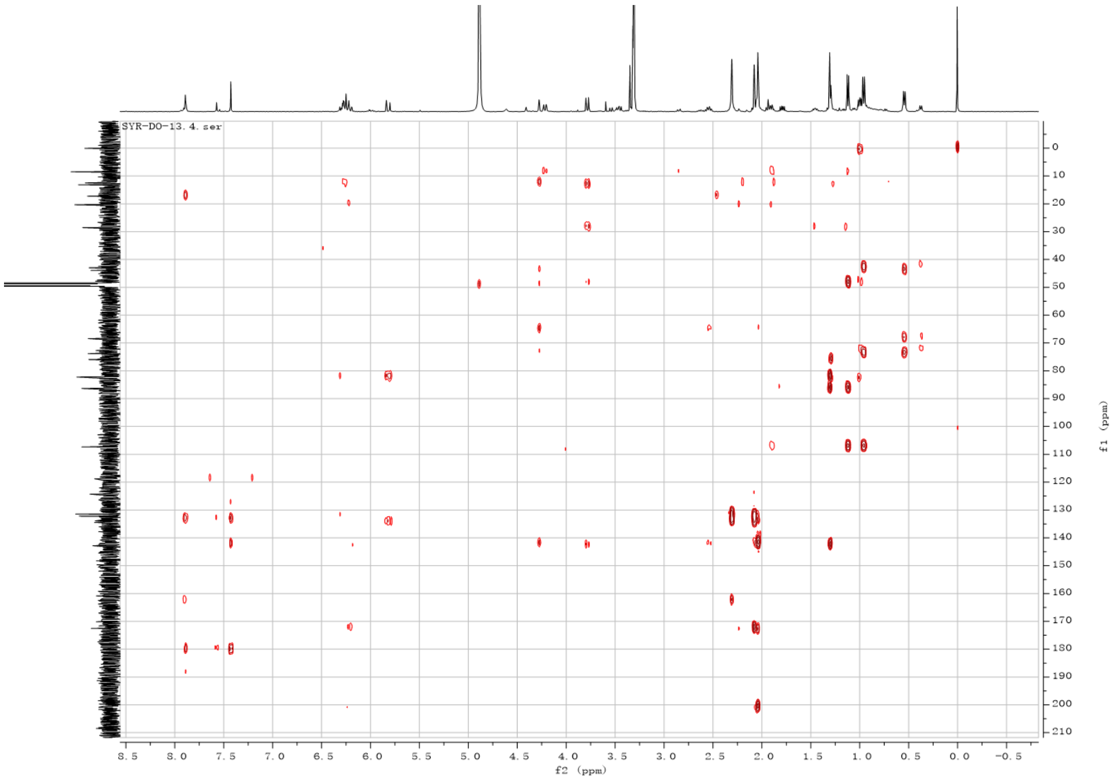

Figure S76. HMBC spectrum of **11** in CD_3OD.

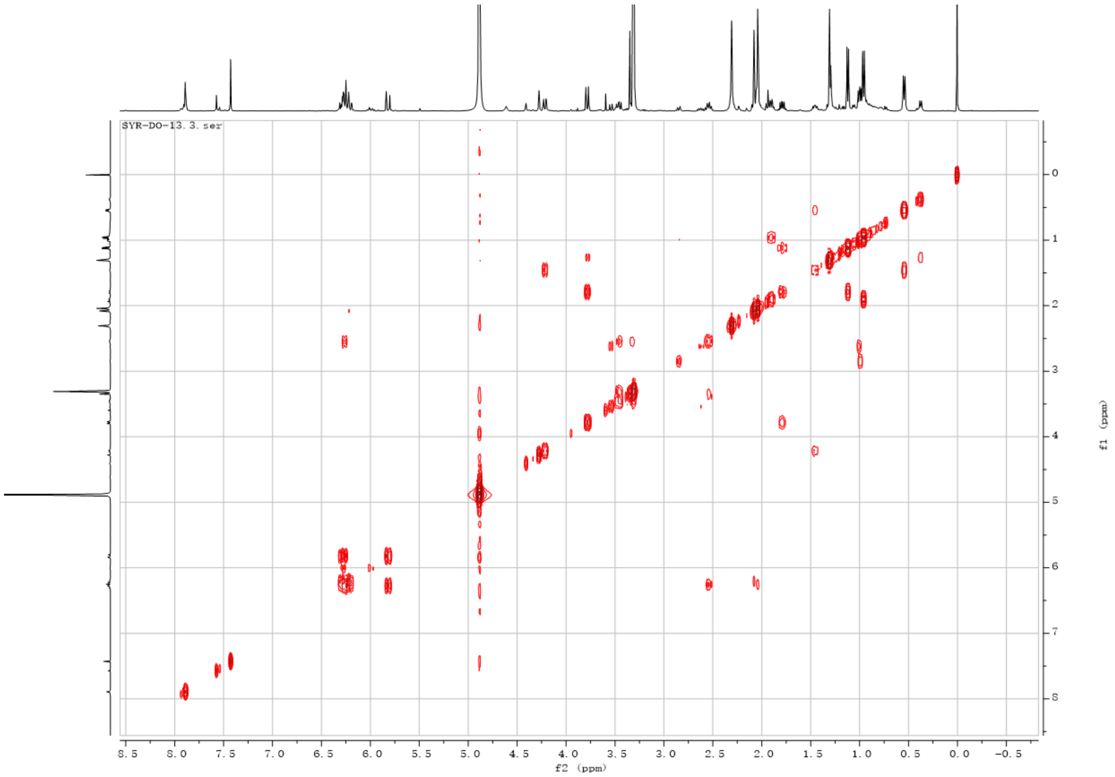

Figure S77. ^1H-^1H COSY spectrum of **11** in CD_3OD.

4. Bioactivity

*4.1 Antimicrobial activity of compounds **1–18** (Figure S78)*

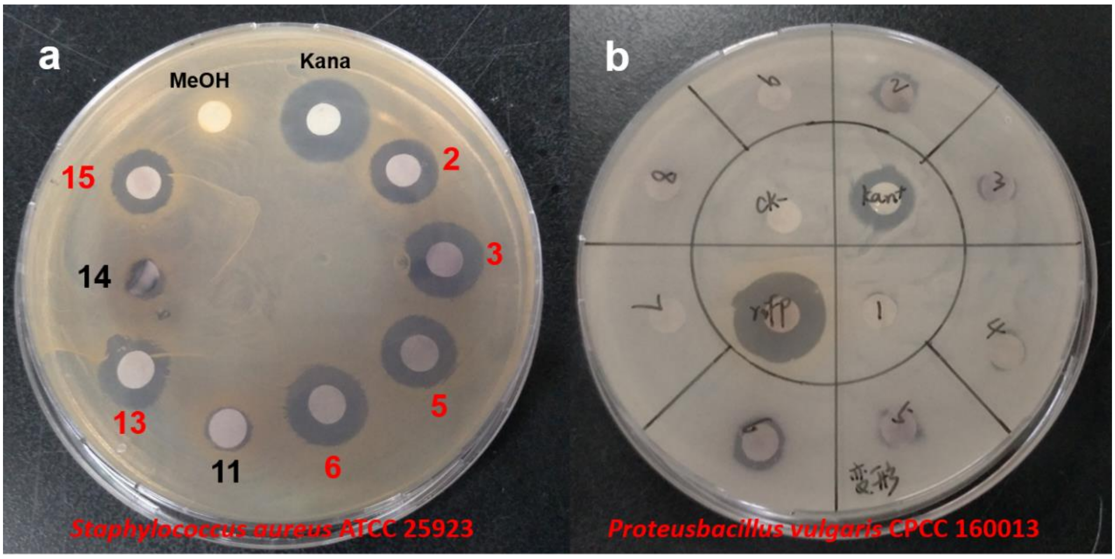

Figure S78. Antimicrobial activity of compounds **1–18.** (a) The inhibitory activity of compounds **1–18** (20 μg each) against *Staphylococcus aureus* ATCC 25923; (b) The inhibitory activity of compounds **1–18** (20 μg each) against the *Proteusbacillus vulgaris* CPCC 160013. In the assays against bacteria, rifampicin and kanamycin were used as the positive control. Methanol was used as the blank control for antimicrobial experiments.

Table S12. Diameter of the inhibition zones and MIC of active compounds against Staphylococcus aureus ATCC 25923.

Test compounds	2	3	5	6	13	15
diameter of the inhibition zones (mm)	12	14	13	15	12	10
MIC (μg/mL)	10	20	20	20	40	20

Table 13. The OD_{600} value of Staphylococcus aureus ATCC 25923 bacterial solution in different concentration gradients of active compounds.

Concentration (μg/mL)	320	160	80	40	20	10	5	2.5	1.25	0.625
**2**	0.0487	0.0473	0.0483	0.0485	0.0665	0.0685	0.4328	0.7804	0.7513	0.8952
**3**	0.0458	0.0446	0.0465	0.0453	0.0923	0.26	0.7743	0.7798	0.8095	0.9738
**5**	0.0497	0.0467	0.0473	0.0483	0.0846	0.1611	0.6428	0.8907	0.9731	1.1691
**6**	0.0428	0.0417	0.046	0.0454	0.0763	0.4787	0.6645	0.867	0.9723	1.2392
**13**	0.0474	0.0459	0.057	0.0873	0.6734	0.9208	1.0208	1.2659	1.3653	1.3515
**15**	0.0504	0.045	0.0509	0.0639	0.0566	0.1278	0.1972	0.5912	0.704	0.9647

Table 14. Antiproliferative activity against KG1 cells of compounds 14-18.

compounds	OD_{480}-1	OD_{480}-2	OD_{480}-3	OD_{480} average	calculated value	growth rate	inhibition rate
**14**	0.295	0.2878	0.3018	0.294866667	0.108436667	9.273877433	90.72612257
	0.2716	0.271	0.2784	0.273666667	0.087236667	7.460780373	92.53921963
	0.8105	0.8656	0.8472	0.8411	0.65467	55.98963456	44.01036544
	1.4018	1.3477	1.3153	1.354933333	1.168503333	99.93443202	0.065567975
	1.4089	1.4328	1.3265	1.3894	1.20297	102.8821401	-2.882140139
**15**	0.6377	0.5611	0.538	0.578933333	0.392503333	33.56823773	66.43176227
	1.0721	1.0962	0.8926	1.0203	0.83387	71.31543613	28.68456387
	1.2205	1.219	1.1652	1.201566667	1.015136667	86.81798615	13.18201385
	1.2933	1.2337	1.1951	1.2407	1.05427	90.16480368	9.835196319
	1.2729	1.3385	1.246	1.2858	1.09937	94.02191111	5.978088893
**16**	0.2741	0.2705	0.2735	0.2727	0.08627	7.378107708	92.62189229
	0.2341	0.2325	0.2329	0.233166667	0.046736667	3.9970808	96.0029192
	0.2188	0.2214	0.2243	0.2215	0.03507	2.99930726	97.00069274
	0.2267	0.2039	0.2379	0.222833333	0.036403333	3.113338522	96.88666148
	0.963	0.9325	0.9556	0.950366667	0.763936667	65.33449645	34.66550355
**17**	0.236	0.2416	0.2404	0.239333333	0.057116333	5.491955128	94.50804487
	0.234	0.2313	0.2377	0.234333333	0.052116333	5.011185897	94.9888141
	0.8773	0.8335	0.8595	0.856766667	0.674549667	64.86054487	35.13945513
	1.4527	1.4084	1.2972	1.3861	1.203883	115.7579808	-15.75798077
	1.5162	1.4173	1.4725	1.468666667	1.286449667	123.6970833	-23.69708333
**18**	0.2406	0.2441	0.244	0.2429	0.060683	5.834903846	94.16509615
	0.2155	0.215	0.2157	0.2154	0.033183	3.190673077	96.80932692
	0.2068	0.2075	0.2584	0.224233333	0.042016333	4.040032051	95.95996795
	0.9891	0.9755	1.0742	1.012933333	0.830716333	79.87657051	20.12342949
	1.2731	1.2964	1.3848	1.3181	1.135883	109.2195192	-9.219519231

4.2 Anti-Type III secretion system (T3SS) activity of compounds ***1-18*** *(Figure S79)*

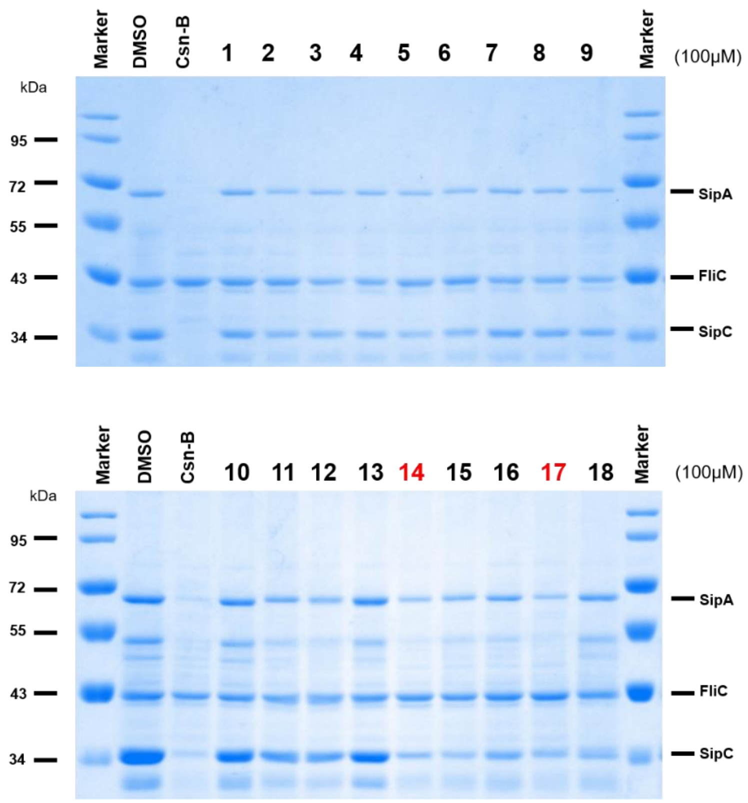

Figure S79. SDS-PAGE analysis of the inhibitory activity of compounds **1–18** (100 μM, respectively) against the T3SS of *S. enterica* Typhimurium UK-1 χ8956. Cytosporone B (Csn-B), an octaketide, isolated from the endophytic fungus *Dothiorella* sp. HTF3 strain, exhibited a strong inhibitory effect on the secretion of SPI-1 effectors (SipA, SipB, SipC, SipD) without evident effects on the flagellar protein FliC. Thus it was used as the positive control. DMSO: vehicle control; SipC: SPI-1 effector protein; FliC: flagellin protein.
